# Identification of treatment elements for adolescents with callous unemotional traits: a systematic narrative review

**DOI:** 10.1186/s13034-024-00792-2

**Published:** 2024-09-03

**Authors:** Pamela M. Waaler, Josefine Bergseth, Linda Vaskinn, Kristin Espenes, Thale Holtan, John Kjøbli, Gunnar Bjørnebekk

**Affiliations:** 1 Department of Special Needs Education, University of Oslo, Oslo, Norway; 2 Center for Child and Adolescent Mental Health, Eastern and Southern Norway, Oslo, Norway; 3 Norwegian Directorate for Children, Youth and Family Affairs (Bufdir), Oslo, Norway; 4 Department of Education, University of Oslo, Oslo, Norway; 5 Norwegian Office for Children, Youth and Family Affairs (Bufetat), Oslo, Norway

**Keywords:** Callous unemotional traits, Common elements, Youth, Adolescents, Review, Treatment, Intervention

## Abstract

Adolescents with callous unemotional (CU) traits are at risk for poor quality-of-life outcomes such as incarceration, suicide, and psychopathy. It is currently unknown which treatments are implemented with CU adolescents specifically and which elements make up these interventions. A narrative systematic review was used to identify the treatments and common elements used with CU adolescents (12–18 years). Eligible studies were randomized controlled trials and quasi-experimental studies evaluating psychosocial interventions, delivered within a clinical context, and directed towards the adolescent or their family. Eight studies with 1291 participants were included. Significant decreases in CU traits were demonstrated only in a minority of studies. The most utilized practice elements were set goals for treatment, practice interpersonal/communication skills, prepare for termination, and teach parents skills and strategies; the most utilized process elements were formal therapy, practice exercises, important others, and flexible/adaptive. A majority of the studies did not report an implementation element. While theoretical frameworks for CU children are employed with CU adolescents, there is still uncertainty regarding their suitability for this age group. Given the limited empirical evidence, there is a critical need for further exploration.

**Supplementary Information:**

The online version contains supplementary material available at 10.1186/s13034-024-00792-2.

## Introduction

Serious antisocial behavior during adolescence is a catalyst for unfavorable developmental outcomes including poor school performance and social interactions, engagement in delinquent behaviors, such as substance abuse, and future antisocial adult outcomes, including arrests and personality disorder diagnosis [49, 56, 63]. This is especially true when these behaviors are exacerbated by callous unemotional (CU) traits [15].

Adolescents with CU traits “are characterized by a lack of guilt and remorse, a lack of concern for the feelings of others, shallow or superficial expression of emotions, and a lack of concern regarding performance in important activities” [25], p. 533). In addition, they use more extreme methods of aggression, are insensitive to punishment cues, and emphasize dominance and revenge when compared to those with serious antisocial behavior alone [23–25, 57]. Even though these traits are not immutable [46, 72], CU traits are relatively stable from adolescence to adulthood (*r* = 0.43–0.60; [25]), and without intervention, CU adolescents are at risk for complications later on in life, such as trouble with the law, substance abuse, homelessness, risky sexual behaviors, and suicide [15]. Furthermore, CU traits “constitute the core affective facets of adult psychopathy” ([15] p. 4), and adolescents who emanate these traits are at an increased risk for psychopathy [15, 27, 46] and committing serious violent crimes as adults [61, 62]. Despite this, systematic empirical evidence is lacking, and this is especially true when it comes to adolescents.

### Treating children and adolescents with CU traits

Considering CU trait stability, the negative quality of life outcomes associated with these traits, as well as the general pessimism that surrounds treating adult psychopathy, treatment is paramount. However, treating CU traits is a monumental task bearing in mind the heterogeneous qualities that characterize this group, such as behavior severity and causal processes, as well as higher rates of treatment dropout, poor participation, treatment non-compliance, and low motivation to change [15, 71].

This does not mean however that treatment is futile, in fact, the treatment of CU traits has been the subject of extensive research since their initial identification in the 1990s. Nevertheless, previous literature has mainly examined treatments for children under the age of 12 [3, 14, 28, 35, 38, 39]. The few studies that do include adolescents often do not incorporate rigorous experimental research designs [7, 52, 58, 64, 67, 70, 71], and/or they examine more general conduct problems such as “disruptive behavior” or “externalizing symptoms” [8, 9, 12, 66].

Meta-analyses that have included both children and adolescent samples with general conduct problems suggest that treatments should include parent management training (PMT) [66] in addition to anger control, problem-solving skills, social skills, assertiveness training, and cognitive–behavioral, family therapy or multisystemic therapy interventions [8, 9]. However, again, these studies do not necessarily examine CU traits specifically, nor do they look exclusively at adolescents.

One systematic review that has examined PMT exclusively with CU adolescents [68] found that those who begin treatment with more severe levels of CU traits often maintain higher CU levels post treatment than adolescents without CU traits, suggesting that certain PMT techniques may yield unequal outcomes for CU adolescents versus regular conduct disordered adolescents. This may be especially true for treatments that emphasize parental discipline as individuals with CU traits tend to be insensitive to punishment and discipline strategies [4, 28].

More recently, Perlstein and colleagues [59] conducted a meta-analysis to determine if treatments for disruptive behavior disorders reduce CU traits (mean age = 10.04). While they did not find an overall treatment effect for CU traits, they did find that CU traits were significantly reduced when treatments incorporated PMT, even after controlling for age, suggesting that PMT is necessary for treating CU traits. However, consistent with the findings from multiple systematic reviews [29, 68, 72], they also found that participants with CU traits began and ended treatment with elevated conduct problems when compared to those with lower levels of CU traits. This does not mean that children with CU traits do not respond to treatment, but rather, CU traits are associated with more severe antisocial behaviors post treatment [59].

Due to the differences in treatment response as well as unequal outcomes for CU adolescents (e.g., punishment vs. reward), one can argue that the “one-size-fits-all” treatment packages currently used may not be optimal. Consequently, there is a knowledge gap in the literature when it comes to treating CU adolescents, and before treatment can be tailored, empirical inquiry must make up for lost time. Therefore, an investigation into CU trait treatment elements is pivotal.

### Common elements

Common elements are approach-specific, model-free, “active ingredients” used in evidence-based treatments to treat specific clinical disorders [33], and include three classifications: practice, process and implementation elements. Practice elements are specific practices or actions (e.g., practice problem solving skills), process elements are the how, when, why, where, for whom, and by whom (e.g., group discussion), and implementation elements are the training and delivery techniques applied to practice and process elements (e.g., supervision).

Uncovering common elements is a blossoming research discipline that shows great promise as their extraction promotes program optimization and enhances an intervention’s efficiency, feasibility, appropriateness, acceptability, and usability, without compromising effectiveness [8, 9, 17, 45]. To date, common element research has focused on distilling intervention elements that address a wide variety of issues including children’s conduct problems [37], parenting behaviors that shape child compliance [40], child abuse [50], academic achievement [17], emotion regulation [31] and child mental health services [16]. Distilling these elements helps identify candidates for further experimental testing and optimize treatment by highlighting effective components. Testing elements experimentally, rather than entire programs, may reveal what works across symptom dimensions, allowing therapists to tailor treatments to clients’ unique needs and avoid harm through the use of ineffective or unsuitable elements.

While element research has not been conducted on CU traits alone, Leijten and colleagues [41] examined the most effective parenting elements for children with disruptive behavior problems. They found that disruptive behaviors are treated best with intervention elements that promote behavior management (e.g., praise), and parental self-management (e.g., emotion regulation). Still, other researchers have augmented PMT with other behavioral treatments such as emotion recognition training and have found improvements in empathy and conduct issues in CU children when compared to PMT alone, suggesting that these may also be essential treatment elements [14]. However, it is still not clear the extent to which these treatment elements are utilized with adolescents.

Furthermore, we do not know if the treatment effects found with CU children are applicable to adolescents as few experimental studies have been conducted with adolescents specifically. In addition, systematic reviews and meta-analyses have all included children under the age of 12 in their results. Thus, we cannot conclude with certainty that these findings are applicable to CU adolescents, leaving many unanswered questions: what treatments are used with CU adolescents specifically, which elements make up these treatments, and are they effective?

### Purpose of the current review

The aim of this systematic review is to review randomized controlled and quasi-experimental studies that have examined changes in adolescent CU traits after they have received psychological treatments. Our aims are three-fold: (1) to examine which psychological treatments are used with CU adolescents, (2) to determine whether these treatments result in significant changes in CU traits, and ultimately (3) to identify the elements that make up these treatments.

We have chosen to examine CU youth specifically for a number of reasons. First, CU traits may manifest differently at various developmental stages, with more serious antisocial behaviors occurring in adolescence (e.g., substance abuse, criminal acts) versus childhood (e.g., temper tantrums, defiance). In addition, the elements applied with young children may not be appropriate for adolescents (e.g., token-based rewards), nor are children necessarily directly involved in treatment (e.g., parent-focused versus youth-focused treatments). Therefore, adolescent interventions may differ from those for children with regard to treatment targets as well as content and delivery. Second, while CU traits may be stable, they also have the potential to decrease or increase across the life span [34] as a result, the malleability of these traits may also vary across different developmental stages. In order to get closer to understanding how the stability and malleability of these traits may influence treatment during adolescence, it is important to examine CU treatment effects on adolescents specifically. Finally, to our knowledge, this is the first empirical attempt to investigate common treatment elements for CU adolescents. As evidenced above, there is an imminent need for further exploration considering the negative quality-of-life outcomes associated with CU traits as well as the absence of information concerning this group.

## Methods

### Protocol registration

The study protocol was registered in the PROSPERO International Prospective Register of Systematic Reviews (identifier CRD42021256143) in May 2021.

### Inclusion criteria (PICO)

The inclusion criteria for this study were:

*Population:* adolescents between 12 and 18 years old with clinical or subclinical levels of CU traits, as determined by psychological assessment (e.g., diagnosis and/or psychometric measure).

*Intervention:* all psychosocial interventions delivered within a clinical context directed towards adolescents or the adolescent’s family. Studies that included pharmacological interventions were still eligible for inclusion if they also included a psychosocial intervention within the clinical context.

*Control:* all types of controls and comparisons (e.g., treatment as usual, waitlist, other active intervention, or no intervention).

*Outcome:* at least one measure for both CU traits and antisocial problem behaviors (e.g., aggression, delinquency, criminal behavior). Self, residential staff, parental, teacher, and clinician reported outcomes were all eligible for inclusion. Both specific CU trait instruments and global measures that measured other CU trait dimensions (e.g., psychopathic traits, narcissism) were eligible for inclusion.

*Study design:* randomized controlled trials (RCTs) and quasi-experimental studies.

### Exclusion criteria

Studies that otherwise met the inclusion criteria were excluded if: (1) adolescents had physical handicaps, developmental disorders (e.g. autism), mental deficiencies, and/or chronic or serious somatic diseases (e.g., asthma, cancer, diabetes, and HIV), (2) the study did not include a control or comparison group (e.g. qualitative or observational studies, pretest–posttest designs, cohort studies, case study), (3) the study did not include post measures for both adolescent CU traits and antisocial behavior(s), (4) the intervention did not include a psychosocial treatment (e.g. medication only, task performance tests), (5) the sample was based on risk without indication of treatment (e.g., “at-risk”, child receiving intervention due to parental incarceration), (6) interventions delivered outside of a clinical setting (e.g. an entire third grade class, population-based community interventions), (7) the work was not published (e.g., abstract or symposia), and (8) the work was published prior to 1990.

Studies were not excluded based on language. While CU traits overlap somewhat with psychopathic traits (e.g., empathy, shallow emotions), psychopathic traits encompass other characteristics (e.g., glibness, superficial charm) that are not associated with CU traits [34], therefore, we chose not to include ‘psychopathic traits’ as a search term in this study.

### Information sources and search strategy

The original search was conducted in June 2021. Three research librarians searched PsychINFO, MEDLINE, Embase, Cochrane Central, ERIC, Web of Science, Sociological Abstracts, Social Care Online, Web of Science, clinicaltrials.gov, WHO International Clinical Trials Registry Platform, and Open Grey databases.

An additional search was conducted in February 2023 due to a delay in the project. Two research librarians searched the same databases as listed above. See Supplementary Material E for an overview of the search strategy employed.

### Study selection

PW, LV, KE, TH, GB, and JK screened the eligible abstracts with Covidence (Covidence Systematic Review Software [13]). All relevant systematic reviews were included in the full-text assessment to determine if any other relevant articles could be identified. Relevant articles identified with this method were already accounted for in the original search, thus no additional resources were found. The full texts of all relevant abstracts were reviewed in duplicate by the authors. Final inclusion decisions were made by PW, KE, JK, GB, and TH. Disagreements were discussed and resolved until the authors reached consensus.

### Data extraction for narrative analysis

During the planning phase of this study, we originally sought to conduct a meta-analysis to determine the most and least effective treatment elements for CU adolescents while moderating for other antisocial behaviors. When it became evident that a meta-analysis was not possible due to the heterogeneity between studies, we decided to uphold these stringent inclusion criteria, nonetheless. This decision was supported by previous findings that children with high CU traits almost invariably display high levels of antisocial behavior, indicating that stable high CU traits rarely occur without concurrent stable high levels of antisocial behavior [15, 22]. Therefore, to be eligible for inclusion, we decided that articles must include both a CU and an antisocial behavior measure at pre- and post-treatment.

We reviewed data using a systematic qualitative synthesis and extracted: (a) study characteristics (author, publication year and type, country of origin, service setting, adolescent age and gender, sample size randomized, inclusion and exclusion criteria, control condition, and length of follow-up—if included), (b) intervention characteristics (name of intervention, duration and intensity, delivery mode, and who delivered the intervention), (c) measurement characteristics (CU trait and antisocial behavior measure used, informant source, pre, post, and if available, follow-up scores, direction of data), and (d) implementation characteristics (acceptance, appropriateness, feasibility, fidelity, and sustainment). Original authors were contacted for more information when outcome measure data was lacking in the original publication. PW and JB extracted the data from each included reference, checked for accuracy, and discussed discrepancies until a consensus was reached.

### Data extraction for element codebook

To extract elements and construct the element codebook, PW and JB read the included study’s methods section for clues on each intervention’s content to create the “coding interface.” Each study had varying amounts of information regarding their respective interventions, therefore, in instances where a thorough description of intervention elements was insufficient [5, 20, 48, 65], intervention manuals were consulted to further inform the coding process.

The coding interface was created in IBM SPSS Statistics (Version 29) [36] through a “consensus mapping procedure” (see supplementary material 2 from [17] for a detailed how-to): PW and JB independently coded each intervention’s *practice* (a specific practice or action: e.g., psychoeducation), *process* (describe the how, when, why, where, for whom, and by whom: e.g., role play), and *implementation* elements (training techniques/delivery of practice and process elements: e.g., supervision) in separate matrices. The coders then reviewed all elements together, discussed, and revised until consensus was reached for each element. All elements and their characteristics were defined in detail as to avoid ambiguity during the coding process. In addition, we chose to define our elements in a highly discrete manner, meaning we were careful to preserve the original definitions provided in the included publications to avoid introducing our own interpretations and understandings to the codebook. Elements that shared similar themes were then grouped together under main categories in a Microsoft Excel spreadsheet (e.g., ‘skill acquisition’ and ‘develop skills’ were grouped under “problem solving skills”) and were given a unique number, resulting in the final element codebook. See Supplementary Material F for the final codebook.

Once the final codebook was completed, PW and JB coded the practice, process, and implementation elements that were described in each original study. Each resource was coded independently. After each coder had completed their task, coding conflicts were resolved through discussion and the independent coding sheets were combined, resulting in one main coding file for analysis.

### Methodological appraisal

The Cochrane Collaboration’s Risk of Bias Tool (Version 2.0) [10] was used to measure the methodological quality of the included RCTs and quasi-experimental designs. PW, LV, and JB preformed the Risk of bias (RoB) assessments. Each included reference was separately assessed as low (unlikely to weaken the effect estimate), high (seriously weakens the effect estimate), or unclear. The raters collaborated together to reach a final RoB rating.

## Results

### Results of the literature search

Our original search in 2021 yielded 23,761 abstracts. Six-thousand eight hundred and seventy duplicates were removed, leaving 16,890 abstracts to be screened; 16,333 were irrelevant. Our supplemental search in February 2023 identified an additional 1392 abstracts. One-hundred and sixty-four duplicates were removed, leaving 1,229 abstracts to be screened; 1197 were irrelevant.

Five-hundred and thirty-nine full texts were retrieved in the original search and 51 more were added after the supplemental search, resulting in a total of 590 full-text articles. In all, 582 studies were excluded, many of which had the wrong outcomes (205 studies) or were conducted with children under the age of 12 (121 studies). Three study protocols may have been relevant for the current review [2, 19, 21], however, the authors had not yet published their findings and an inclusion decision could not be made.

**Fig. 1:**
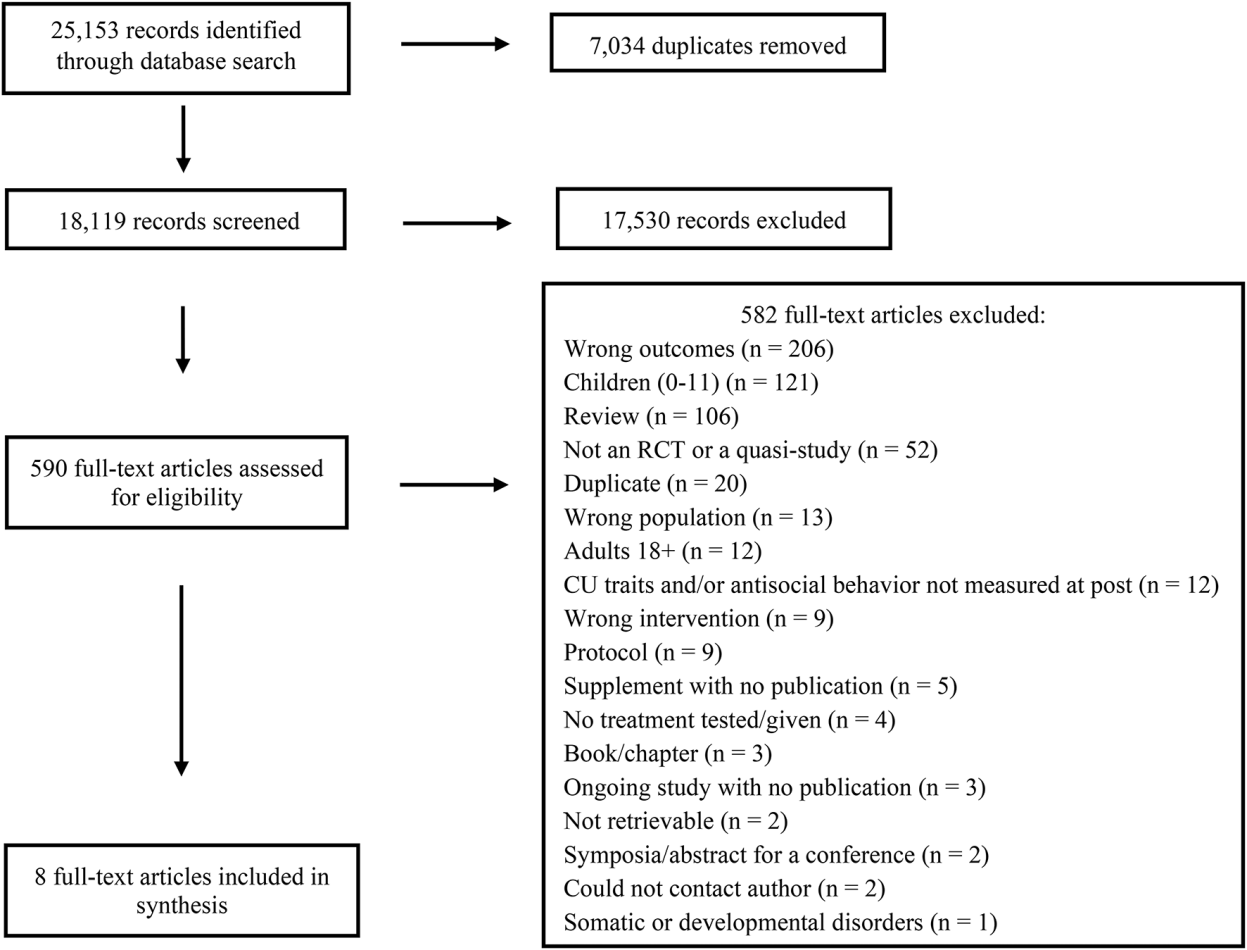
PRISMA flowchart. PsychINFO, MEDLINE, Embase, Cochrane Central, ERIC, Web of Science, Sociological Abstracts, Social Care Online, Web of Science, clinicaltrials.gov, WHO International Clinical Trials Registry Platform, and Open Grey databases were searched

In total, eight studies met criteria for final inclusion (see the PRISMA diagram in Fig. 1). Two of the resources in our search were not retrievable. The first [30] was a dissertation. Both EBSCO and ProQuest were searched as well as a general Google search to no avail. The second [43] was a withdrawn protocol. A complete list of excluded texts and details regarding reasons for exclusion is available in Supplementary Material D.

### Methodological quality

The Cochrane Collaboration’s Risk of Bias Tool (Version 2.0) [10] was used to measure the methodological quality of the included articles. All eight studies were assessed for their sequence generation, allocation concealment, blinding of patients, personnel, and outcome assessors, incomplete outcome data, and selective outcome reporting. A summary of the overall RoB is presented in Table 1. Most of the studies received an unclear risk of bias score. However, a higher proportion of studies received a high risk of bias score under the incomplete outcome data for all outcomes category (50% of the studies). See Supplementary Material A for the RoB score deviations for each included study.

**Table 1:**
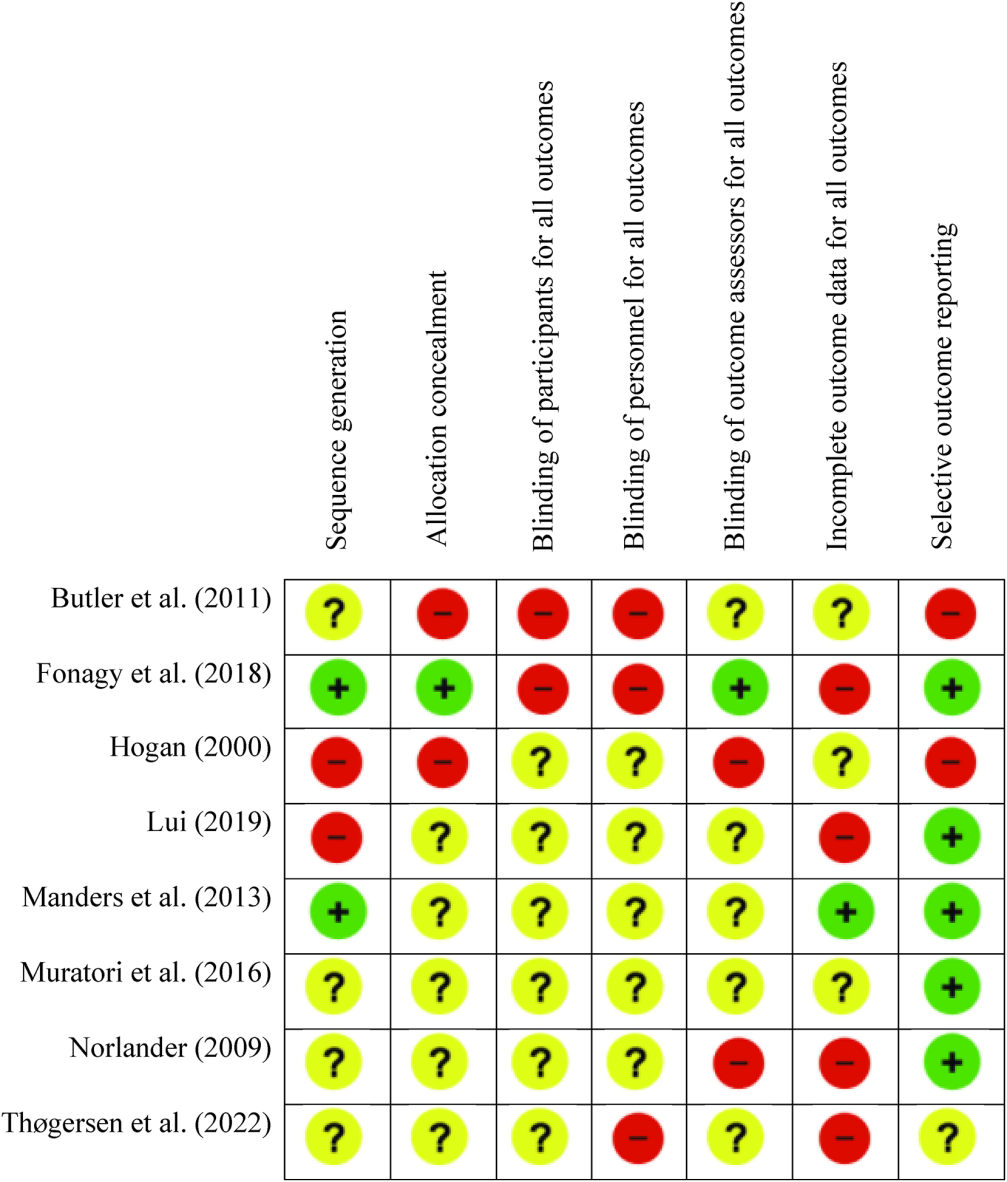
Cochrane Risk of Bias scores for included studies

### Overview of included studies

Eight interventions from eight studies with 1,290 participants in total (694 randomized to a treatment group) were identified. The included studies were published between 2000 and 2022; A majority of the interventions (*n* = 5) utilized some form of therapy (Multisystemic Therapy, Cognitive Behavioral Therapy, Family Focused Therapy), one study incorporated training (Emotion-Processing Skills Training), and two studies ran their own intervention adaptations (Coping Power and a structured intervention based off of Goldberg’s theory of malevolence). Three studies [20, 44, 65] included a follow-up period after post measures, while five did not.

All of the primary study interventions were aimed at adolescents, however 50% of the studies [5, 20, 48, 65] involved both the adolescent and their family. The duration of treatment ranged from eight to 52 weeks. Five studies were conducted via an outpatient service setting. Seven studies incorporated an active control group for intervention comparison (e.g., TAU, another intervention), and one study [32] used a passive waitlist-control group. See Table 2 for an in-depth description of each primary study.

**Table 2:** Included study characteristics and outcome measures

Study	Country	Sample characteristics				Treatment			CU measure	Follow-up
		*n*_*T*_	*n*_*C*_	Gender	Age range (mean)	Intervention & treatment group target	Duration	Service setting		
Butler et al. [5]	England	56	52	82.4% Male	(15.1)	(1) MST (2) TAU Family therapy	11–30 weeks (M = 20.4)	Outpatient Clinical Care	Psychopathic traits: APSD (CR);YAPSD (SR)	n/a
Fonagy et al. [20]	England	305	279	63.4% Male	(13.8)	(1) MST (2) TAU Family therapy	12–20 weeks (M = 19.86)	Outpatient Clinical Care	Callous unemotional traits: ICU (CR & SR)	52 weeks 78 weeks
Hogan [32]	USA	8	8	100% Male	12–18 (15.4)	(1) Goldberg’s theory of malevolence (2) Waitlist Group format	8 weeks	Residential Facility	Psychopathic traits: PSD-Y (SR)	n/a
Lui [44]	USA	27	19	83.9% Male	16–18 (16.5)	1. EPST 2. TAU Group format	8 weeks	Quasi Military Camp	Callous unemotional traits: ICU (CR & SR)	6 weeks 12 weeks
Manders et al. [48]	Netherlands	147	109	73.4% Male	(16.0)	(1) MST (2) TAU Family therapy	16–24 weeks	Outpatient Clinical Care	Callous unemotional traits: ICU (CR); Psychopathic traits: APSD narcissism & impulsiveness subscales (CR)	n/a
Muratori et al. [53]	Italy	28	27	92.7% Male	13–15	(1) Coping Power (2) Beyond the Clouds Group format	52 weeks	Outpatient Clinical Care	Callous unemotional traits: ICU (SR)	n/a
Norlander [55]	USA	36	30	85% Male	14–18 (15.4)	1. CBT 2. TAU Group therapy	8 weeks	Secure Setting	Psychopathic traits: PCL: YV interpersonal, affective, lifestyle, & antisocial features subscales and total score (SR); APSD callous-unemotional, impulsivity & conduct problems subscales and total score (SR); SALE (SR)	n/a
Thøgersen et al. [65]	Norway	87	72	54.1% Male	10–17 (14.7)	1. FFT 2. TAU Family therapy	12–24 weeks	Outpatient Clinical Care	Callous unemotional traits: ICU (CR, SR, & TR)	78 weeks

Length of follow-up indicates length of time after baseline measures were administered

*CBT* Cognitive Behavioral Therapy, *CR* Caregiver-report, *CU* Callous Unemotional, *EPST* Emotion-Processing Skills Training, *FFT* Functional Family Therapy, *ICU* Inventory of Callous Unemotional Traits, *n/a* not applicable, *MST* Multisystemic Therapy, *PCL-YV* Psychopathy Checklist– Youth Version, *PSD-Y* Psychopathic Screening Device– Youth Version, *SALE* Survey of Attitudes and Life Experiences, *SR* Self-report, *TAU* treatment as usual, *TR* Teacher-report, *YAPSD* Youth Antisocial Screening Device

### Description of populations

All studies were aimed at adolescents between 12 to 18 years old, however Thøgersen et al. [65] included children as young as 11. While the age range of this study fell outside of the inclusion criteria for the current review, it was still eligible because the sample mean age for the study fell between 12 and 18 years old (*M* = 14.7). Most of the studies also included both male and female adolescent participants (*n* = 7), while one study (13%) included male-only participants [32].

Three publications (38%) included participants living in the United States, while five were conducted in Europe: two in England [5, 20], one in the Netherlands [48], one in Italy [53], and one in Norway [65]. Five studies (63% [5, 20, 48, 53, 65]) were conducted with adolescents receiving outpatient services, one study [44] took place within a quasi-military camp setting, one study [55] involved adolescents in a secure setting, and one study [32] took place within a child welfare residential facility. A description of the included primary studies’ populations is available in Table 2. Please see Table 3 for an overview of each studies’ antisocial behavior inclusion criteria as well the descriptive statistics reported for their respective intervention groups at baseline.

**Table 3:** Inclusion criteria and baseline statistics for antisocial behavior in the included studies’ intervention groups

Study	Antisocial behavior inclusion criteria	Youth self-report antisocial measure	Mean	SD	Caregiver youth-report antisocial measure	Mean	SD
Butler et al. [5]	Court referral order to treatment, under supervision order for at least 3 months, or following imprisonment	YSR– Externalizing behavior	53.80	10.70	CBCL– Externalizing behavior	67.70	8.40
		YSR– Aggression	59.10	10.40	CBCL - Aggression	69.40	12.90
		YSR– Delinquency	65.10	8.80	CBCL - Delinquency	73.40	8.30
		SRYB– Delinquent behavior BAS - Antisocial thinking BAS - Tolerance for aggression BAS - Criminal sentiments Delinquent Peers	83.30 67.50 8.90 30.90 28.20	4.60 19.60 5.00 8.60 9.20			
Fonagy et al. [20]	Moderate-to-severe antisocial behavior indicated by at least three severity criteria (e.g., a history of offending or at risk of offending, placed outside of the home, truancy, excluded from school) across multiple settings. At least one of the following general inclusion criteria for antisocial behavior: persistent and enduring violent and aggressive behavior, at least one conviction plus three additional convictions or warnings, conduct disorder diagnosis and not responsive to treatment, permanent school exclusion due to antisocial behaviors, and/or significant risk of harm to self or others	SDQ– Conduct problems	5.00	2.10	SDQ– Conduct problems	6.60	2.40
		Self-reported Delinquency Measure– Variety of delinquent acts Self-reported Delinquency Measure– Volume of delinquent acts Self-reported Delinquency Measure– Variety of substance misuse Self-reported Delinquency Measure– Volume of substance misuse Self-reported Delinquency Measure– Peer delinquency ABAS– Antisocial beliefs and attitudes	4.80 19.70 0.80 1.60 5.00 60.80	3.60 18.30 1.70 3.70 4.70 23.10			
Hogan [32]	Resident at a center for teens with behavioral problems	YSR– Total score YSR– Externalizing behavior	65.90 65.80	12.00 10.30			
Lui [44]	Adolescents placed in a voluntary quasi-military style program who either dropped out of school or were at risk of dropping out	YSR– Social problems	5.71	3.53	CBCL– Social problems	5.14	4.19
		YSR– Externalizing behavior	11.16	4.72	CBCL– Externalizing behavior	11.47	7.56
Manders et al. [48]	Severe and violent antisocial behavior	YSR– Externalizing behavior	12.40	9.25	CBCL– Externalizing behavior	23.32	12.60
Muratori et al. [53]	Oppositional defiant disorder or conduct disorder diagnosis determined by K-SADS				CBCL– Externalizing behavior	72.59	5.91
					CBCL– Rule breaking behavior	67.80	5.76
Norlander [55]	Placement at a juvenile justice alternative education setting due to misconduct	STAXI-2– Trait anger STAXI-2– Angry temperament STAXI-2– Angry reaction STAXI-2– Anger expression index STAXI-2– Anger expression in STAXI-2– Anger expression out Incident reports^a^ Arrests and criminal charges^a^ Problems worksheet– Number of problems Problems worksheet– Problem frequency Problems worksheet– Problem severity	22.44 8.72 9.17 49.97 17.65 18.97 0.25 0.03 6.91 8.94 5.37	6.36 2.96 2.76 10.90 3.86 4.15 0.55 0.17 3.97 1.71 1.44			
Thøgersen et al. [65]	Adolescents who engage in or were at risk for: delinquency, aggressive or violent behavior, verbal aggression or threats, truancy, school-related problem behavior, and/or drug use				CBCL– Aggression	13.92	8.66
					CBCL– Rule breaking behavior	9.20	5.03

*ABAS* Antisocial Beliefs and Attitudes Scale, *BAS* Basic Assumptions Scale, *CBCL* Child Behavior Checklist ASEBA, *K-SADS* Kiddie Schedule for Affective Disorders and Schizophrenia, *SDQ* Strength and Difficulties Questionnaire, *SRYB* Self-report of Youth Behavior, *STAXI-2* State-Trait Anger Expression Inventory 2, *YSR* Youth Self-report ASEBA

^a^Data collected from official records

### Description of intervention implementation

Treatment adherence was reported in five [20, 44, 48, 55, 65] of the included studies. Adherence was measured via supervision, training, boosters, and consultations. Therapist fidelity was reported in three studies [5, 20, 44] via fidelity measures, implementation reviews, and therapist questionnaires. Acceptability was measured in four of the included studies [32, 44, 55, 65] via participant (adolescent and/or parent) rated satisfaction, level of enjoyment, and quality of treatment.

Other important implementation indicators such as adoption (e.g., intention to employ the intervention), appropriateness (e.g., the perceived fit or relevance), cost–benefit, feasibility (e.g., resources to carry out the intervention), penetration (e.g., integration of an intervention within the service setting), and sustainability (e.g., the maintenance or sustained use over time) were not measured. See Table 5 under “implementation elements” for more details.

### Treatment outcomes

Callous Unemotional traits were measured with a variety of measures, six measures in all, including measures for CU traits specifically, measures for psychopathic traits, as well as full scales (e.g., Inventory of Callous Unemotional Traits) and subscales of full-scale psychopathic trait measures (e.g., narcissism, impulsiveness, antisocial features, CU traits) In addition, studies included both adolescent self-report and caregiver adolescent-report (see Table 2).

Effect sizes (ES) were reported by authors in a variety of formats, or not reported at all, therefore, we calculated ES (Cohen’s *d*) using the raw data reported for each pre-post outcome in each individual study (i.e., CU trait measures). Comparing changes across groups from pre to post treatment was chosen since it includes all the study information available versus comparing post group means alone. Due to the heterogenous nature of the studies, we determined using a pooled pretest standard deviation (*SD)* was not appropriate. As such, ES were calculated based on the pre-post mean change in the treatment group divided by the pretreatment *SD* minus the pre-post mean change in the control group divided by the pre control *SD* [51]. Effect sizes were interpreted according to Cohen’s [11] recommendations: small effect = 0.20, medium effect 0.50, large effect = 0.80.

Due to the nature of the outcome measures (lower score equals improvement), a negative ES is indicative of improvement. Therefore, in our case, a negative ES indicates that the intervention group fared better than the control group on the given outcome measure. Associations between treatment and CU trait symptom changes were reported in all eight studies. See Table 4 for a narrative synthesis of each study’s outcomes and their limitations.

**Table 4:** Callous unemotional trait outcomes for adolescent self-report measures, Caregiver adolescent-report measures, and Study limitations

Study	Adolescent self-report CU outcomes	Caregiver adolescent-report CU outcomes	Post-hoc analyses	Limitations
Butler et al. [5]	Psychopathic traits decreased pre-post treatment (*d* = -0.05), however these decreases were not statistically significant between MST and TAU	Psychopathic traits decreased in the MST group pre-post treatment* (*d* = -0.44)		Unclear process of change Insufficient power Sample included fewer chronic and violent offenders than in the US Merits of the TAU condition not described
Fonagy et al. [20]	Callous unemotional traits decreased pre-post treatment (*d* =– 0.12), at 52– week follow-up (*d* =– 0.11), and at 78– week follow-up** (*d* =– 0.27). Statistically significant decreases were only found at 78-week follow-up for MST	Compared to TAU, MST resulted in decreases in callous unemotional traits pre-post treatment*** (*d =*– 0.37), pre-52-week follow-up (*d* =– 0.06), and pre-78-week follow up (*d* =– 0.07). These differences were only statistically significant pre-post.	For adolescents with low CU traits at baseline: MST was detrimental compared to TAU For adolescents with high CU traits at baseline: High CU scores at baseline did not moderate the effect of MST	Heterogenous TAU group MST not as flexible as TAU Some scales were not internally consistent
Hogan [32]	Psychopathic traits did not significantly change pre-post treatment (*d* = 0.90)			Treatment developed from theory based on a case study Exercises not suitable for residential treatment facility Small sample size (*n* = 16) No long-term follow-up Teacher reports not completed No random sampling Group heterogeneity Findings not generalizable
Lui [44]	Callous unemotional traits decreased in the EPST group pre-post treatment (*d* =– 0.44), pre-6-week follow-up* (*d* =– 0.84) and pre-12-week follow-up (*d* =– 0.49). These decreases were only statistically significant at 6-week follow-up.	Callous unemotional traits did not decrease pre-12-week follow-up* (*d* = 0.86)^a^	Higher self-reported CU traits at baseline were positively correlated with higher self-reported externalizing problems (*r =* 0.38) and poorer emotional recognition (*r =* 0.43). Higher self-reported CU traits at baseline were negatively correlated with peer-reported isolation (*r = −* 0.30), suggesting higher CU traits are associated with greater isolation. Parent reported CU traits were not significantly related to any outcomes. CU traits did not moderate changes in affective perspective taking, empathy, or externalizing problems.	Small magnitude of change Small sample size (*n* = 56) Predominantly male sample High attrition No random assignment
Manders et al. [48]		Callous unemotional traits (*d* =– 0.21), narcissism (*d* =– 0.09), and impulsiveness (*d* =– 0.19) decreased pre-post treatment in the MST group, but none were statistically significant	For adolescents with lower psychopathic traits: MST was more effective than TAU in reducing post-treatment externalizing problems. This finding was consistent across both adolescent self-report and parent report. For adolescents with higher psychopathic traits: there was no significant differences in the effectiveness of MST versus TAU in reducing externalizing problems. This finding was consistent across both adolescent self-report and parent report.	No adolescent self-report for psychopathic traits US scale norms cannot be generalized with the sample Low power No follow-up data
Muratori et al. [53]	Callous unemotional traits decreased in the treatment group pre-post treatment* (*d* =– 0.86)			No random assignment Small sample size (*n* = 55) Heterogenous sample
Norlander [55]	Psychopathic traits (PCL: YV) (*d* =– 0.24), and interpersonal (*d* =– 0.22), affective (*d* =– 0.34), lifestyle (*d* =– 0.32), and antisocial features (*d* =– 0.10) decreased in the CBT group pre-post treatment, but none were statistically significant. There was a decrease in callous unemotional traits (*d* =– 0.24), impulsivity conduct problems (*d* =– 0.12), and psychopathic traits (APSD) (*d* =– 0.26) in the CBT group, but none were statistically significant. There was a decrease in psychopathy in the CBT group (SALE), pre-post treatment (*d* =– 0.12), but it was not statistically significant		Adolescents in the higher psychopathy group showed positive changes in their attitudes towards treatment with moderate increase in Readiness to Change Index scores (*d* = 0.67). Changes in readiness to change scores were less pronounced in the lower psychopathy group. Adolescents with higher psychopathy scores had a significant reduction in their psychopathy scores across all PCL-YV subscales (*d* = 0.35 to 0.69). PCL-YV scores increased among participants in the lower psychopathy group. Despite these increases, the treatment group showed smaller increases compared to the comparison group.	Post-test PCL: YV was not masked to group membership Adolescents unexpectedly released Short post-assessment (8 weeks after treatment start) No follow-up Small sample size (*n* = 72) Sample had lower levels of psychopathy than other populations Limited generalizability
Thøgersen et al. [65]		The FFT group did not result in larger decreases in callous unemotional traits from pre-post treatment (*d* = 0.07), but FFT did have a larger decrease pre-78-week follow-up (*d* =– 0.13). *Note: Statistical significance not reported on separate groups (full-sample analyses)*	There was a significant short-term decrease (mean change of– 3.45 scale points; SE = 1.31, *p* = 0.008) in CU traits for youth who scored above the normative cutoff score. However, the long-term change was not statistically significant, suggesting an immediate reduction in CU traits after treatment, but it was not sustained over the long-term.	No adolescent self-report Did not include measures for other psychopathy dimensions (narcissism and impulsivity) Did not take into consideration CU trait typologies Behavioral problem heterogeneity

*p < 0.05; **p < 0.01; ***p < 0.001 Blank cells indicate that no measures or records were administered, used, or indicated in the included articles

*APSD* Antisocial Process Screening Device, *CBT* Cognitive Behavioral Therapy, *CU* Callous Unemotional, *EPST* Emotion-Processing Skills Training, *FFT* Functional Family Therapy, *MST* Multisystemic Therapy, *PCL-YV* Psychopathy Checklist– Youth Version, *SALE* Survey of Attitudes and Life Experiences, *TAU* treatment as usual

^a^Caregiver ICU measure was not administered at post or 6-week follow-up

#### CU trait pre-post measures

Six out of eight studies (all except [48] and [65]) utilized at least one total score from an adolescent -self report CU measure. Five of them [5, 20, 44, 53, 55] saw decreases in CU traits for their respective treatment groups from pre-post treatment, however, these decreases were only statistically significant in Muratori et al. [53], *d* = − 0.86, *p* < 0.05). Psychopathic traits did not decrease from pre to post treatment in Hogan [32].

When it comes to caregivers, four studies utilized total scores from caregiver respondents during the pre-post phase [5, 20, 48, 65], out of these four studies, two reported a statistically significant decrease in CU traits from pre-post treatment (Butler et al. [5]: *d* = − 0.44, *p* < 0.05; Fonagy et al. [20]: *d* = − 0.37, *p* < 0.001). In Thøgersen et al. [65], the treatment group did not experience decreases in CU traits from pre to post (*d* = 0.07).

Two studies collected data from CU measure subscales: Manders et al. [48] included narcissism and impulsiveness subscales (parent child-report) while Norlander [55] examined interpersonal, affective, lifestyle, antisocial features, callous-unemotional, impulsivity and conduct problem subscales (adolescent self-report). There were decreases in all subscales for the treatment groups in each respective study, however, none were statistically significant.

#### CU follow-up measures

Adolescent follow-up data was collected in two studies: Fonagy et al. [20] and Lui [44]. Decreases in CU traits were found in Fonagy et al. [20] at both 52-week (*d* = − 0.11) and 78-week (*d* = − 0.27) follow-up but was only statistically significant at 78 weeks. Lui [44] also found decreases in CU traits at 6- (*d* = − 0.84) and 12-week (*d* = − 0.49) follow-up, however these decreases were statistically significant only at 6-week follow-up (*p* < 0.05).

Three studies, Fonagy et al. [20], Lui [44], and Thøgersen et al. [65] collected caregiver adolescent-report data during follow-up. Fonagy et al. [20] reported decreases in adolescent CU traits at 52- (*d* = − 0.06) and 78-week (*d* = − 0.07) follow-up, but they were not statistically significant. Lui [44] found a significant increase in CU traits at 12-week follow up (*d* = 0.86, *p* < 0.05). Finally, Thøgersen et al. [65] found a decrease in CU traits at 78-week follow-up (*d* = − 0.13), however it is important to note that significance could not be determined by the information provided by the authors. None of the included studies utilized subscale data from either adolescents or caregivers during follow-up.

Taken together, all studies, except Hogan [32], adolescent report) and Lui [44] at 12-week follow-up (caregiver report), reported a decrease in their treatment groups’ CU traits either post treatment or at follow-up. However, it is important to note that these decreases were only statistically significant from pre to post treatment in Muratori et al. ([53], adolescent measure), Fonagy et al., ([20], caregiver measure), and Butler et al., ([5], caregiver measure), and only at 78-week follow up in Fonagy et al. ([20], adolescent measure) and 6-week follow-up in Lui ([44], adolescent measure). Overall, there appears to be limited statistically significant evidence regarding decreases in CU traits after psychosocial treatment.

#### CU post-hoc analyses

While none of the included studies originally set out to distinguish how adolescents with high versus low CU traits respond to treatment, five out of eight conducted post-hoc analyses [20, 44, 48, 55, 65] to examine how those who scored high on CU trait measures faired after treatment versus those who scored low (see Table 4). Fonagy et al., [20] found that MST was detrimental for participants who scored low on CU traits whereas high CU trait scores did not moderate effect. While higher self-reported CU traits at baseline were positively correlated with higher self-reported externalizing problems and poorer emotional recognition in Lui [44], no significant moderations by any subgroups were found within this study. Manders et al. [48] on the other hand found that MST was more effective than TAU in reducing post treatment externalizing problems for adolescents with lower psychopathic traits, and this finding was consistent across both adolescent self-reports and parent reports. For adolescents with higher psychopathic traits, no significant differences were found. In Norlander [55], participants with higher psychopathy scores showed positive changes in their attitudes toward treatment and a reduction in their psychopathy scores, whereas changes in readiness were less pronounced and overall psychopathy scores increased among participants with lower psychopathy scores. Finally, Thøgersen et al. [65] examined a subgroup of adolescents with elevated CU traits. They found a significant short-term decrease in CU traits immediately following FFT, however, long-term changes were not statistically significant, suggesting that changes were not sustained over time.

All in all, these studies vary in their evidence regarding treatment outcomes in adolescents with high levels of CU traits versus low. On the one hand, high CU adolescents may experience a reduction in psychopathy scores and an increase in attitude towards treatment, however a majority of the included studies either did not find any significant changes or found contradicting evidence in favor of those with lower CU scores. Therefore, as it stands, the evidence remains inconclusive.

### Common treatment elements for adolescents with CU traits

In the eight included studies, 64 practice, 36 process, and nine implementation elements were found (see Supplementary Material B and C). The total coding agreement between coders was 76.6%. The mean number of coding inputs per intervention was 33.25 (*SD* = 16.41).

The 64 practice elements were categorized under 11 main common practice element categories (see the bolded elements in Supplementary Material B). The most common practice elements (more than 50% of the studies) were: set goals for treatment (6 studies: [5, 20, 32, 48, 53, 65]) practice interpersonal/communication skills (6 studies: [5, 20, 32, 48, 55, 65]), prepare for termination of intervention (5 studies: [5, 20, 32, 48, 65]), and teach parents skills and strategies to effect change in relevant domains (5 studies: [5, 20, 48, 53, 65]). See Supplementary Material B for a complete overview of how many studies employed each element and their definitions, and Table 5 for each specific study.

**Table 5:** Common practice elements and discrete practice elements in each study and their frequencies

Study	Frequencies	Practice elements	Process elements	Implementation elements
Butler et al. [5]	Common practice elements *N* = 6 Discrete practice elements *N* = 15 Process elements *N* = 12 Implementation elements *N* = 1 Coding agreement = 81.5%	*Organization* Set goals for treatment Prepare for termination of intervention Increase contact quality with the community Identify risk/protective factors in the community *Training in Emotional Recognition and Differentiation* Learn to identify triggers for different types of emotions *Training in Preventing Maladaptive Behavioral Response to Emotional Distress* Reduce substance use *Parent Skills Training* Teach parents skills and strategies to effect change in relevant domains Increase parental supervision/monitoring Enhance interpersonal support *Cognitive Skills* Focus on the present Accepting responsibility *Social Skills Training* Practice interpersonal/communication skills Increase/decrease contact with peers Enhance involvement in prosocial activities Improve family relationships	Formal therapy Location of treatment Important others Support on demand Regular support Family influence Flexible/adaptive Individualized Engagement Social-ecological Strengths based Refer to additional support	Therapist fidelity
Fonagy et al. [20]	Common practice elements *N* = 5 Discrete practice elements *N* = 13 Process elements *N* = 11 Implementation elements *N* = 3 Coding agreement = 88.9%	*Organization* Set goals for treatment Prepare for termination of intervention Increase contact quality with the community Identify risk/protective factors in the community *Training in Preventing Maladaptive Behavioral Response to Emotional Distress* Reduce substance use *Parent Skills Training* Teach parents skills and strategies to effect change in relevant domains Increase parental supervision/monitoring Enhance interpersonal support *Cognitive Skills* Focus on the present Accepting responsibility *Social Skills Training* Practice interpersonal/communication skills Enhance involvement in prosocial activities Improve family relationships	Formal therapy Location of treatment Important others Regular support Family influence Flexible/adaptive Individualized Engagement Social-ecological Strengths based Refer to additional support	Therapist fidelity Supervision Consultations
Hogan [32]	Common practice elements *N* = 8 Discrete practice elements *N* = 26 Process elements *N* = 17 Implementation elements *N* = 1 Coding agreement = 71.9%	*Organization* Set goals for treatment Review progress and/or celebrate change Session review/integration of information Prepare for termination of intervention Alliance with facilitator, group members, or caregivers *Training in Emotional Recognition and Differentiation* Learn to recognize basic emotions Practice expressing/communicating emotions Learn how thoughts contribute to feelings Practice emotion recognition/awareness in daily life Practice to avoid assumptions about how others might feel/their intentions *Psychoeducation* Psychoeducation on anger Psychoeducation on the connection between events, thoughts, and feelings Psychoeducation about treatment/treatment techniques *Increase Motivation* Use of positive reinforcement *Self-exploration of Thoughts and Feelings* Objects like me exercise Write a story or draw a picture of an event that was life changing Explore the feeling of anger Explore/improve self-esteem *Training in Preventing Maladaptive Behavioral Response to Emotional Distress* Anger/agression management *Cognitive Skills* Evaluate consequences of behavior Practice identifying thinking errors Give personal examples of thinking errors *Social Skills Training* Review and discuss group format and group rules Ice-breaking exercise Encourage group cohesion Practice interpersonal/communication skills	Psychoeducation Role-play Rotate role-play Homework Homework reviewed Group discussion Practice exercises Anger thermometer Index cards Feedback on performance Peer feedback Group performance Reward based Youth influence Multicomponent Flexible/adaptive Feedback from participant	Participant satisfaction
Lui [44]	Common practice elements *N* = 4 Discrete practice elements *N* = 13 Process elements *N* = 14 Implementation elements *N* = 5 Coding agreement = 82.9%	*Training in Emotional Recognition and Differentiation* Learn to recognize basic emotions Learn to identify emotions from various modalities Learn to identify triggers for different types of emotions Learn to infer the emotional states of others through hypothetical situations Practice expressing/communicating emotions Practice emotion recognition/awareness in daily life Learn to infer the emotional states of others through real life scenarios *Psychoeducation* Psychoeducation on emotion recognition Psychoeducation on emotion awareness Psychoeducation on perspective taking *Increase Motivation* Use of positive reinforcement Enhancing motivation and engagement *Self-exploration of Thoughts and Feelings* Personal benefits/self-interests related to intervention elements	Psychoeducation Role-play Rotate role-play Group discussion Modeling Practice exercises Games Clips Static stimuli Peer feedback Group performance Multicomponent Pedagogical principles Feedback from participants	Therapist fidelity Supervision Group training Participant satisfaction Participant acceptability
Manders et al. [48]	Common practice elements *N* = 5 Discrete practice elements *N* = 15 Process elements *N* = 14 Implementation elements *N* = 3 Coding agreement = 78.6%	*Organization* Set goals for treatment Review goals for treatment Assign tasks required to accomplish treatment goals Prepare for termination of intervention Increase contact quality with the community Identify risk/protective factors in the community *Training in Preventing Maladaptive Behavioral Response to Emotional Distress* Reduce substance use *Parent Skills Training* Teach parents skills and strategies to effect change in relevant domains Increase parental supervision/monitoring Enhance interpersonal support *Cognitive Skills* Focus on the present Accepting responsibility *Social Skills Training* Practice interpersonal/communication skills Enhance involvement in prosocial activities Improve family relationships	Formal therapy Location of treatment Practice exercises Important others Support on demand External monitoring Regular support Family influence Flexible/adaptive Individualized Engagement Social-ecological Strengths based Refer to additional support	Boosters Supervision Consultations
Muratori et al. [53]	Common practice elements *N* = 7 Discrete practice elements *N* = 10 Process elements *N* = 7 Coding agreement = 56.5%	*Organization* Set goals for treatment *Training in Emotional Recognition and Differentiation* Learn to infer the emotional states of others through hypothetical situations *Psychoeducation* Psychoeducation on perspective taking *Problem Solving Skills* Practice problem solving skills *Self-exploration of Thoughts and Feelings* Explore the feeling of anger *Training in Preventing Maladaptive Behavioral Response to Emotional Distress* Anger/aggression management *Parent Skills Training* Teach parents skills and strategies to effect change in relevant domains *Social Skills Training* Review and discuss group format and group rules Resisting peer pressure Increase/decrease contact with peers	Formal therapy Psychoeducation Role-play Practice exercises Important others Reward based Multicomponent	
Norlander [55]	Common practice elements *N* = 4 Discrete practice elements *N* = 4 Process elements *N* = 3 Implementation elements *N* = 2 Coding agreement = 81.8%	*Training in Preventing Maladaptive Behavioral Response to Emotional Distress* Modify contextual cues of criminal opportunity *Cognitive Skills* Teach cognitive reframing and restructuring of cognitive distortions *Stress Management* Stress inoculation training *Social Skills Training* Practice interpersonal/communication skills	Role-play Group discussion Feedback from participants	Consultations Participant involvement
Thøgersen et al. [65]	Common practice elements *N* = 8 Discrete practice elements *N* = 29 Process elements *N* = 19 Implementation elements *N* = 3 Coding agreement = 55.2%	*Organization* Set goals for treatment Review goals for treatment Assign tasks required to accomplish treatment goals Review progress and/or celebrate change Session review/integration of information Prepare for termination of intervention Discussion of experience during treatment/intervention/element Alliance with facilitator, group members, or caregivers Increase contact quality with the community *Increase Motivation* Enhancing motivation and engagement *Problem Solving Skills* Practice problem solving skills Planning for the future *Self-exploration of Thoughts and Feelings* Exploring the youth’s perspectives and opinions *Training in Preventing Maladaptive Behavioral Response to Emotional Distress* Alternative actions to maladaptive behavior Reduce negativity and blame *Parent Skills Training* Teach parents skills and strategies to effect change in relevant domains Clarify and establish parental expectations Enhance interpersonal support *Cognitive Skills* Teach cognitive reframing and restructuring of cognitive distortions Practice validation Accepting responsibility Minimize hopelessness/increase hope Change meaning *Social Skills Training* Practice interpersonal/communication skills Resisting peer pressure Increase/decrease contact with peers Identify/describe relational functions Conflict management and negotiation skills Improve family relationships	Formal therapy Homework Homework reviewed Modeling Practice exercises Important others Nonjudgmental approach Support on demand Feedback on performance External monitoring Family influence Culturally sensitive Multicomponent Flexible/adaptive Individualized Engagement Strengths based Refer to additional support Feedback from participants	Supervision Participant satisfaction Participant appropriateness

Common practice elements are italicized. Total number of common practice elements = 11; total number of discrete practice elements = 64: blank cells indicate no information was provided by the authors

The most common process elements overall (over 50% of the studies) were formal therapy (5 studies: [5, 20, 48, 53, 65]), practice exercises (5 studies: [32, 44, 48, 53, 65]), important others (5 studies: [5, 20, 48, 53, 65]), and flexible/adaptive (5 studies: [5, 20, 32, 48, 65]).

A majority of the included studies (more than 50%) did not report an implementation element, however, four studies accounted for adherence through supervision [20, 44, 48, 65]. See Supplementary Material C for a complete overview of how many studies employed each process and implementation element and their definitions, and Table 5 for specific studies.

In all, common practice elements ranged from 4–29 per study, with Norlander [55] employing the least (four) and Thøgersen et al. [65] employing the most (29). The number of process elements per study ranged from 3–19, again with Norlander [55] with the least, and Thøgersen et al. [65] with the most. Finally, seven of eight studies (all but [53]) reported at least one implementation element. Lui [44] utilized the most with five.

## Discussion

This systematic review addressed three key questions regarding treatment for adolescents with CU traits. First, it examined which psychological treatments are used with CU adolescents when both CU traits and an antisocial behavior are measured, second, it assessed whether these treatments resulted in significant changes in CU traits, and third, it revealed the components that make up these treatments. Regarding the first question, our search revealed six unique interventions. A majority of the interventions (63%) utilized formal therapy, one intervention focused on emotional training, and the last two interventions were author-developed treatments. All of the interventions were aimed at adolescents, while half of them also incorporated the adolescent’s family. Treatment duration ranged from 8 to 52 weeks, and over half of them (63%) took place in an outpatient clinical care setting. In sum, the findings show that not only are adolescents with CU traits an understudied group, but there is also variation in the types of treatment offered to CU adolescents, indicating that treatment is complex and multifaceted.

In addressing the second, we found minimal evidence regarding decreases in adolescent CU traits after treatment. While 78% of the included studies measured decreases at some point in time, either at post or if applicable at follow-up, (*d* = − 0.86–− 0.02), these decreases were only statistically significant via adolescent self-report in two studies (Muratori et al. [53], *d* = − 0.86; Lui [44], *d* = − 0.84) and via caregiver adolescent-report in another two studies (Fonagy et al. [20], *d* = − 0.37; Butler et al. [5], *d* = − 0.44). Taken all together, these findings suggest that there is limited evidence in the treatment CU adolescents, highlighting the necessity for more studies to build a more comprehensive understanding.

Finally, in regard to our third question, we identified 11 main common practice element categories, 64 practice, 36 process, and nine implementation elements, offering valuable insight into what has been implemented with CU adolescents over the past two decades. Overall, “Social Skills Training” (‘practice interpersonal/communication skills’), “Organization” (‘set goals for treatment’; ‘prepare for termination of intervention’), “Cognitive Skills” (‘accepting responsibility’), “Training in Preventing Maladaptive Behavioral Response to Emotional Distress”, and “Parent Skills Training” (‘teach parents skills and strategies to effect change in relevant domains’) were used in more than 50% of the studies. “Training in Emotional Recognition and Differentiation”, “Psychoeducation”, “Increase Motivation”, “Problem Solving Skills”, “Self-exploration of Thoughts and Feelings”, and “Stress Management” were also present but used less so. In regard to process elements, ‘formal therapy’, ‘practice exercises’, ‘important others’, and ‘flexible/adaptive’ were utilized most. Lastly, more than 50% of the included studies did not incorporate an implementation element.

### Theory versus reality

Researchers in the past have worked to establish theories regarding effective treatment approaches for children and adolescents with CU traits. These theories are built upon previous studies, reviews, and meta-analyses that have mainly examined conduct problems and disruptive behaviors in general, while fewer have examined CU traits specifically. Overall, in treatments for CU traits, the research base has endorsed PMT, anger control, problem-solving skills, social skills, assertiveness training, and interventions that involve cognitive–behavioral, family- or multisystemic therapy [8, 9, 66]. Interestingly, we see all of these elements, except assertiveness training, represented in our results, suggesting that the theoretical treatment strategies for CU children are also applied to CU adolescents.

#### Practice element implications

Looking specifically at PMT, PMT elements that promote behavior management (e.g., praise) and parental self-management (e.g., emotion regulation) have been singled out for general disruptive behavior in the past [41]. While we do not see these specific elements in our included studies, we see a few related to promoting behavior management, namely teaching parents skills and strategies and clarifying/establishing expectations. However, similar elements related to parental self-management were not present.

Nonetheless, parent skills training was well represented in five of our included studies (see Table 5). This is interesting considering Perlstein et al.’s [59] finding in their meta-analysis that PMT has a significant effect on CU traits, even after controlling for sample age. In our study, we see that PMT is also used to a larger degree with CU adolescents. While there were positive effects on adolescent CU traits at some point in time in the studies that included PMT (*d* = − 0.44–− 0.05), not all were statistically significant. One possible explanation for this finding is that parenting programs used with small children may not be suitable for adolescents, for example, certain reward-based parental strategies, such as token-based systems, may not be applicable with teens.

Despite this, there is reason to believe that parenting interventions are still necessary, even in later developmental stages: one longitudinal cohort study found that more parental physical punishment was associated with increases in CU traits from ages 13 to 24 [47], while another more recent longitudinal study with twins [60] found that genetic factors primarily influence the reciprocal relationship between negative parental discipline and CU traits during mid childhood. However, as youth reach late childhood (around age 12), shared environmental influences, such as aspects of the family environment, begin to play a more significant role, suggesting that the relationship between negative parenting and CU traits extends beyond genetic factors alone. While the evidence regarding PMT with adolescents is modest, future studies should continue to focus on identifying essential PMT elements for adolescents with CU traits.

PMT has also been examined in the past with other treatment modalities; for example*,* Dadds and colleagues [14] found that PMT augmented with emotion recognition training (ERT) for children with complex conduct problems had positive effects on conduct problems and empathy for children with CU traits, suggesting that ERT may also lead to significant improvements for CU children*.* In our study, we found four studies (see Table 5) that employed ERT as a main common element category, however, the maximum number of studies for specific common elements under this category was two (see Supplementary Material B), indicating that there has in fact been little focus on emotion recognition for CU adolescents. Interestingly, the treatment group in one study [44] received Emotion-Processing Skills Training (EPST), and the author was able to demonstrate that EPST had positive effects on adolescent CU traits at both pre-post treatment and at follow-up, indicating that ERT may also be a necessary pursuit for future research.

#### Process element implications

Previously, researchers have emphasized that CU individuals are a heterogenous group, and that trait severity and stability are dependent on many factors including genetic typology, psychopathology variants, and environmental differences [71]. Therefore, a one-size-fits-all treatment approach may not be advisable, and in light of this, it seems important that treatments for CU adolescents are not only multimodal, but also flexible and individualized.

In our review, we found some evidence suggesting that treatments are multimodal (4 studies), flexible (5 studies), and individualized (4 studies). However, a notable gap exists regarding whether treatment is tailored to the unique needs of the adolescents themselves, with only one study [32] mentioning specifically that adolescents had the opportunity to influence treatment (note that family influence was mentioned in four studies, but the degree to which the adolescents’ opinions and preferences were considered is unknown). Moreover, all interventions were standardized with obligatory treatment phases. This contradicts the idea of a flexible treatment approach tailored to the specific needs of adolescents with CU traits [7, 35]. Currently, our findings suggest that treatment for CU adolescents appears to follow a one-size-fits-all pattern, which may not be well-suited for this target group.

While we may not be able to conclude in our study whether multimodality, flexibility, and individualization are important process elements for CU adolescents, moving forward, it is important to explore whether these process elements are essential in addressing the complexity of CU trait heterogeneity. Therefore, we suggest that testing common elements is the next logical step in the development of personalized treatments. Without these insights, it will be challenging to transition away from the prevailing idea of one-size-fits-all to a “what works best for whom” treatment perspective.

#### Moving forward: measuring motivation

When CU adolescents present to treatment, they often encounter increased dropout rates, diminished motivation, and lower levels of participation and treatment compliance [15, 71]. Their limited tendency to engage in social interactions, build relationships, or connect with others detrimentally impacts their willingness to engage in treatment [15]. To address these challenges, researchers not only propose that treatment should be flexible and personalized due to CU trait heterogeneity, as discussed above, but it should also promote treatment engagement. Enhanced treatment engagement, in turn, contributes to improvements in treatment participation, motivation, and compliance among CU adolescents [15, 35]. However, the inherent nature of CU traits may hinder not only one’s willingness to engage in treatment but also negatively influence their motivation to comply and complete the therapeutic process. Therefore, it is essential that research studies with CU adolescents take their degree of participation and compliance into account.

None of the included articles in our study incorporated measures for participation or compliance, nor degree of motivation. While one study did measure adolescent involvement [55] and another measured adolescent acceptability [44], none provided insight into whether adolescents (and their families) actively participated or adhered to treatment. This is an interesting observation considering the relationship between these elements and treatment outcomes. Previous studies with CU children and adolescents have found that motivation focused treatment tactics, such as reward-oriented contingency management and positive reinforcement, reduce recidivism and conduct problems and increase treatment outcomes [6, 7, 28, 54].

Reflecting on the current review, ‘positive reinforcement’ was identified as a practice element in just two of our included studies, ‘increase motivation’ was identified in three, and two studies employed ‘rewards-based’ process elements, however, none examined whether these elements were directly related to degree of motivation or its variation over time. Therefore, there is no direct indication of motivation among CU adolescents during treatment in the included studies. While attrition in the included studies may offer some indication, it falls short of providing a clear understanding of adolescent motivation as an important implementation element. Ultimately, we cannot determine with certainty whether CU adolescents are actively attending, willing to participate, complying to treatment, or motivated to change.

### Suggestions for future research

Due to the nature of this review, the exact effects these treatment elements have on adolescent CU traits must remain speculative. However, this does not limit us in regard to suggestions for future research.

First, while previous studies have examined conduct problems and disruptive behaviors in general, a limited number of empirical studies have examined CU traits specifically, with even fewer targeting CU adolescents. This was evident in our review as only eight aligned with our criteria. It is evident that empirical inquiry directed towards CU traits has not been prioritized, and even less so for adolescents. Given the substantial personal and societal costs associated with CU traits, prioritizing treatment during adolescence should be a fundamental public health concern. While there is a growing call for CU trait prevention and treatment endeavors to be initiated in early childhood [15, 59, 69], this is not always feasible due to challenges such as limited access, limited resources, lack of awareness, stigma, denial, and insufficient parental involvement. Therefore, establishing a focus on adolescents and advocating for timely interventions is imperative.

In addition, because CU adolescents by nature are less willing to engage in treatment, future studies should also include measures of adolescent participation, compliance, and motivation to determine how these factors influence outcomes. Finally, as evidenced by our strict inclusion criteria, few studies have examined antisocial behavior alongside CU traits. This is interesting considering those with high levels of CU traits also display high levels of conduct problems [22]. Therefore, in order to develop a more comprehensive understanding of how CU traits moderate treatment outcomes for various antisocial behaviors, future studies should include antisocial behavior measures.

Second, based off of previous positions [28], one might conclude that the absence of evidence in our study regarding a decrease in CU traits may be attributable to the belief that higher CU traits limit responsiveness to intervention. However, rather than this absence of effect being a reflection of CU trait immutability, it may be a reflection of rigidity in regard to how treatment benefits are currently defined. Indeed, there is a growing perspective that instead of examining whether participants reach normalization at the end of treatment, perhaps magnitude of change should also be taken into consideration [1, 18]. Changing our position from regarding treatment *outcome* as an indicator of effect to treatment *response* may challenge the notion that having these traits consistently predicts poorer treatment responses.

Third, based on our findings, there are numerous elements available for further testing, but perhaps a logical place to start is with PMT and emotion recognition. While the current evidence does not indicate whether both are essential for treating CU traits, previous literature indicates that treatments should incorporate parent management skills and conscience development, however, these elements have primarily been applied in studies involving children under the age of 12. This could pose a challenge as adolescents may require different treatment components compared to younger children; for instance, adolescents might benefit from more multisystemic interventions that encompass their friends and school. Nevertheless, the landscape is multifaceted, and these elements are not a means to an end. Treating adolescents with CU traits is an intricate affair that requires nuanced interventions to address the unique challenges each individual adolescent presents. However, to move closer to an answer, it is vital that the effectiveness of the specific elements found in our study are empirically tested with adolescents.

Hence, our final suggestion: the effectiveness of specific CU trait treatment elements must be evaluated. There are various strategies one can employ for this evaluation, such as identifying shared components among interventions, examining the impact of element presence on therapy outcomes, and conducting microtrials and factorial experiments [42]. In this vein, we originally sought to test the impact of element presence on therapy outcomes with a meta-analysis. However, this was challenging due to the included studies’ heterogeneity. As a result, we focused solely on identifying shared elements among interventions, which was valuable as it serves as a groundbreaking step towards testing these elements with microtrials (testing the effect of single elements) and factorial experiments (randomly assigning participants to single components or a combination of components).

Empirically testing these elements in research trials is important as it goes beyond simply revealing correlations like systematic reviews and meta-analyses do. We need to start asking which treatments, if any, have documented effectiveness. Once we gain a clearer understanding of the effectiveness of these elements, we move one step closer towards developing new innovative approaches for CU adolescents. This is particularly important considering the indication that a one-size-fits-all approach with standalone treatments may not be optimal for individuals with CU traits due to the complex interplay between CU trait mechanisms (e.g., genetic factors, environmental factors, and neurocognitive issues) and treatment outcomes. Identifying specific treatment elements that contribute to improvements in CU traits could enhance intervention efficiency, ensuring that CU adolescents receive targeted care tailored to their needs.

### Strengths and limitations

This study has a number of strengths. To our knowledge, this is the first study that summarizes the existing literature for CU adolescents exclusively. Many studies have examined children under the age of 12 in the past, but very few have examined adolescents specifically. Therefore, there is a need for more experimental studies targeting adolescents. Another strength of ours was our decision to keep the coding categories broad. We did this to help us gather a clear picture of the literature and ensure transparency. We were extra mindful of this given the previous lack of knowledge regarding interventions used with CU adolescents. We thought this would be more beneficial than relying on our own interpretations and categorizations as it would help us steer clear of introducing our own personal biases to the results.

With strengths, come limitations. First, the included studies utilized different measures for CU traits, some used total score scales while others used subscales, indicating uncertainty of what construct is being measured. Furthermore, the included articles used both CU trait specific instruments as well as more global measures of psychopathic trait dimensions (e.g., psychopathic traits, impulsiveness, narcissism). CU traits are distinct from other psychopathy dimensions and including global measures may blur the specific outcomes related to CU traits alone. This mixing of measures may potentially bias the results, particularly when assessing interventions aimed specifically at CU traits. Ultimately, this may reflect non-uniformity within the field regarding how CU traits should be measured. In addition, our study is heavily dependent on the treatment elements that are prevalent in the research literature, a phenomenon called “popularity bias” [17]. As a result, elements that have not been extensively studied in controlled research are not reflected in our findings.

Another limitation of ours is uncertainty around the onset of CU traits—whether they emerged during adolescence or if they have been present since childhood. When CU traits emerge during childhood, which they typically do, they are more enduring and more challenging to treat [26]. Distinguishing whether they appear during childhood or adolescence is important in understanding whether treatment elements should differ for individuals based on when the traits first emerged.

A third limitation in our study is that we included all eight studies, regardless of their methodological quality. Attrition was observed in the majority of studies, which is unfortunate as those who drop out may be the ones who need the intervention the most. In addition, a number of included studies were underpowered. Low power undermines our ability to confidently assume that these interventions are effective, posing a risk of making both type 1 and type 2 errors. These factors, when taken together, may likely have limited our ability to draw reliable conclusions.

Another limitation of ours is that we were constrained by what was included in the articles and the manuals we consulted. For example, “teaching parents skills and strategies” is in itself not traditionally a common element since it can be viewed as a “package” that can be further unpacked (e.g., which skills?). Therefore, we are unable to determine which specific skills were taught due to the level of detailed reporting in the original studies. In the same light, while positive reinforcement or establishing parental expectations may occur during MST, they were not explicitly mentioned in the articles or treatment manual. As a result, studies that utilized MST did not receive codes for these treatment elements. The opposite can also be true: the presence of an element in an article or manual does not guarantee its implementation, so critical questions linger such as who received which elements, how frequently, and at what intensity? While therapist fidelity could offer insights, it was only assessed in three studies. Another similar limitation is linked to adolescent effort; just because an element was taught does not mean it was practiced. Therefore, we are limited in our ability to say with certainty that each individual treatment element was accounted for, that they were applied appropriately, and that they were practiced by the adolescent.

Our final limitation stems from our decision to only include studies that assessed both CU traits and an antisocial behavior at both pre- and post-treatment. This strict criterion may have limited the pool of eligible studies and hindered our ability to construct a more comprehensive overview of treatments used with CU adolescents.

## Conclusion

Adolescence is a sensitive developmental age ripe with new windows of opportunity to improve positive life trajectories. Therefore, it is crucial that treatments for adolescents with CU traits are explored further. Our findings serve as an intervention map; however, it is important to note that this map does not offer an indication of the success or failure of these elements, and the use of many elements does not guarantee success, nor does the use of few elements negate it. Nonetheless, the landscape remains nuanced, and the lack of experimental studies with CU adolescents raises doubts about whether the theoretical frameworks designed for younger children are suitable for this age group.

Given the limited empirical evidence, there is a critical need for further exploration. Our project made a significant stride towards filling this gap by systematically evaluating the treatment elements used with CU adolescents. This endeavor aimed to bridge the divide between theory and reality by offering a more comprehensive understanding of the interventions currently utilized with CU adolescents. Through our inquiry, we have opened the black box of branded treatments for CU adolescents, which we hope will help pave the way for future researchers to empirically test treatment elements.

## Electronic supplementary material

Supplementary Material 1

Supplementary Material 2

Supplementary Material 3

Supplementary Material 4

Supplementary Material 5

Supplementary Material 6

## Data Availability

All relevant data and material have been included in the appendices available in the supplementary material.

## Code availability

Please contact the first author for a copy of the common element coding file.
