# Supplementary material for: Identification of treatment elements for adolescents with callous unemotional traits: a systematic narrative review
Source: Child Adolesc Psychiatry Ment Health. 2024 Sep 3;18:110. doi: 10.1186/s13034-024-00792-2 (PMC11373131; doi:10.1186/s13034-024-00792-2)
Supplement: Supplementary file 1 — Supplementary Material 1 [file 13034_2024_792_MOESM1_ESM.pdf]

Title: Identification of Treatment Elements for Adolescents with Callous Unemotional Traits: A Systematic Narrative Review
Journal: Child and Adolescent Psychiatry and Mental Health
Authors: Pamela M. Waaler, Josefine Bergseth, Linda Vaskinn, Kristin Espenes, Thale Holtan, John Kjøbli, and Gunnar Bjørnebekk
Correspondence author: Pamela M. Waaler, Ph.D. candidate Department of Special Needs Education, University of Oslo; E-mail: p.m.waaler@isp.uio.no

Supplementary Material A

Risk of Bias Score Deviations for each Included Study

Reference	Risk of Bias Score Deviations
Butler et al. (2011)	High score for allocation concealment: MST supervisors informed patients of their allocation.<br>High score for blinding of participants and personnel: blinding was not possible<br>High score for selective outcome reporting: SFIT scale outcomes not reported<br>Not enough information was provided to determine: sequence generation, allocation concealment, blinding of outcome assessors, and incomplete outcome data. Unclear score
Fonagy et al. (2018)	High score for blinding of participants and personnel: masking was not possible<br>High score for incomplete outcome data: high attrition rates in both groups
Hogan (2000)	High score for sequence generation: participants were assigned in order of their completion of the assessment battery<br>High score for allocation concealment: participants knew which group they belonged to<br>High score for blinding of outcome assessors: measures were completed under the direct supervision of the principal investigator<br>High score for selective outcome reporting: No report was provided on post-group evaluation<br>Not enough information was provided to determine: blinding of participants and personnel and incomplete outcome data. Unclear score
Lui (2019)	High score for sequence generation: participants were not truly randomly assigned due to residential facility restricitons<br>High score for incomplete outcome data: high attrition<br>Not enough information was provided to determine: allocation concealment, blinding of participants and personnel, and blinding of outcome assessors. Unclear score
Manders et al. (2013)	Not enough information was provided to determine: allocation concealment, blinding of participants and personnel, and blinding of outcome assessors. Unclear score
Muratori et al. (2016)	Not enough information was provided to determine: sequence generation, allocation concealment, blinding of participants and personnel, blinding of outcome assessors, and incomplete outcome data. Unclear score

Norlander (2009)	High score for blinding of outcome assessors: the post-test PCL:YV administration was not masked to group membership<br>High score for incomplete outcome data: high attrition<br>Not enough information was provided to determine: sequence generation, allocation concealment, and blinding of participants and personnel. Unclear score
Thøgersen et al. (2022)	High score for blinding of outcome assessors: questionnaires will filled out with a research assistant present in the room<br>High score for selective outcome reporting: impulsivity (SNAP) not included in descriptive statistics (table 1), but in table 2 for estimated parameters<br>Not enough information was provided to determine: sequence generation, allocation concealment, blinding of participants and personnel, and incomplete outcome data. Unclear score
