# Supplementary material for: Identification of treatment elements for adolescents with callous unemotional traits: a systematic narrative review
Source: Child Adolesc Psychiatry Ment Health. 2024 Sep 3;18:110. doi: 10.1186/s13034-024-00792-2 (PMC11373131; doi:10.1186/s13034-024-00792-2)
Supplement: Supplementary file 2 — Supplementary Material 2 [file 13034_2024_792_MOESM2_ESM.pdf]

Title: Identification of Treatment Elements for Adolescents with Callous Unemotional Traits: A Systematic Narrative Review
Journal: Child and Adolescent Psychiatry and Mental Health
Authors: Pamela M. Waaler, Josefine Bergseth, Linda Vaskinn, Kristin Espenes, Thale Holtan, John Kjøbli, and Gunnar Bjørnebekk
Correspondence author: Pamela M. Waaler, Ph.D. candidate Department of Special Needs Education, University of Oslo; E-mail: p.m.waaler@isp.uio.no

Supplementary Material B

Common Practice Elements, Definitions and Frequencies

Practice element	Definition	N
**Organization**		**6**
Set goals for treatment	Review or discuss the aims of the interventions, review goal attainment or set goals for treatment	6
Review goals for treatment	Discuss expectations, nature of intervention	2
Assign tasks required to accomplish treatment goals	Assign homework, assigned tasks for youth/family to do either during session or in between sessions	2
Review progress and/or celebrate change	Therapist validation, reflections, deferring to previous sessions	2
Session review/integration of information	Summarizing current session, connecting session to previous sessions	2
Prepare for termination of intervention	Generalize learned skills, extend positive changes into new situations/contexts	5
Discussion of experience during treatment/intervention/element	Discuss positive or negative experiences with the treatment	1
Alliance with facilitator, group members, or caregivers	Focus on developing/nurturing alliance	2
Increase contact quality with the community	Increase contact with school, other services	4
Identify risk/protective factors in the community	Discuss factors in the youth's everyday life that can be harmful or helpful to treatment/improvement	3
**Training in Emotional Recognition and Differentiation**		**4**
Learn to recognize basic emotions	Recognize expressions in self and others: happy, sad, anger, fear, disgust, surprise	1
Learn to identify emotions from various modalities	Facial, auditory, postural	1
Learn to identify triggers for different types of emotions	Discussion of the relation between emotions and social contexts, providing real-life examples	2
Learn to infer the emotional states of others through hypothetical situations	Practice understanding another's emotion with the use of hypothetical situation	2
Practice expressing/communicating emotions	Practice appropriate responses, learn how to communicate emotion in a constructive way	2
Learn how thoughts contribute to feelings	Explain how thoughts occur with emotional experiences; how thoughts and emotions are connected	1
Practice emotion recognition/awareness in daily life	Be aware of incidents that raise feelings of anger, sadness, joy, or fear	2
Practice to avoid assumptions about how others might feel/their intentions	Learn that people have different perceptions of similar situations	1
Learn to infer the emotional states of others through real life scenarios	Youth discuss how they believe others felt during a given situation	1
**Psychoeducation**		**3**
Psychoeducation on emotion recognition	Importance of emotion recognition (ER) for daily functioning; learn cues to attend to improve own ER	1
Psychoeducation on emotion awareness	Focus on increasing awareness of emotions in self and others	1
Psychoeducation on perspective taking	Focus on the importance and personal benefits of understanding others' perspectives	2

Psychoeducation on anger	How anger can build if feelings are not addressed, escalation of anger	1
Psychoeducation on the connection between events, thoughts, and feelings	Understand that events, thoughts, and feelings are interconnected	1
About treatment/treatment techniques	General information about treatment; teach benefits/explain the reason for using specific techniques	1
**Increase Motivation**		**3**
Use of positive reinforcement	Reward desirable outcomes (e.g., praise or tangible benefit)	2
Enhancing motivation and engagement	Motivation for change, adopt collaborative stance, respect youths' autonomy, encourage clients	2
**Problem Solving Skills**		**2**
Practice problem solving skills	Use role-play or discussions to practice skills	2
Planning for the future	Create prevention-relapse plan, plan for futures stresses	1
**Self-exploration of Thoughts and Feelings**		**4**
Exploring the youths perspectives and opinions	Understand youth's perspective about emotions and empathy; challenge youth's inconsistent beliefs	1
Objects like me exercise	Youth compare themselves to an inanimate object and discuss how they are similar	1
Write a story or draw a picture of an event that was lifechanging	Designed to increase self-awareness and understand own values and how these have affected their beliefs	1
Explore the feeling of anger	Connect how people and events contribute to anger; discuss how angry a youth gets in a given situation	2
Explore/improve self-esteem	Help youth recognize what others perceive as their positive characteristics	1
Personal benefits/self-interests related to intervention elements	See the link between specific treatment elements and the youth's own best interest	1
**Training in Preventing Maladaptive Behavioral Response to Emotional Distress**		**7**
Alternative actions to maladaptive behavior	Alternatives to choose when presented with triggers for maladaptive behavior	1
Anger/aggression management	Modification of contextual threat cues	2
Modify contextual cues of criminal opportunity	Limit access to social contexts that create negative opportunities	1
Reduce negativity and blame	Between family members, own actions, therapist interrupts negative interactions	1
Reduce substance use	Limit/eliminate youth substance misuse	3
**Parent Skills Training**		**5**
Teach parents skills and strategies to effect change in relevant domains	Address communication problems, encourage school attendance and achievement	5
Increase parental supervision/monitoring	Caregivers know where their youth is, reduce youth's association with delinquent peers	3
Clarify and establish parental expectations	Caregivers explain and maintain expectations for their youth	1
Enhance interpersonal support	Enhance outside family relationships, increase support from social networks	4
**Cognitive Skills**		**6**
Teach cognitive reframing and restructuring of cognitive distortions	Think about thoughts differently e.g., overvaluing the self at the expense of others	2
Evaluate consequences of behavior	The positive and negative consequences of utilizing specific actions e.g., physical aggression	1
Practice identifying thinking errors	Learn that thoughts can be inconsistent with behaviors	1
Give personal examples of thinking errors	Youth gives examples of how their thoughts can be inconsistent with their behaviors	1
Focus on the present	Emphasize here-and-now	3
Practice validation	Recognize others' feelings, situations, "truths"	1
Accepting responsibility	Taking responsibility for self (thoughts, actions, behaviors)	4
Minimize hopelessness/increase hope	Emphasize hopeful experiences, normalize, problems can be solved	1
Change meaning	Re-labeling and reframing of attributions and experiences, create a narrative	1
**Stress Management**		**1**
Stress inoculation training	Exposure to stress in a controlled way	1
**Social Skills Training**		**7**
Review and discuss group format and group rules	Collaborate to decide rules that should apply to the group	2
Ice-breaking exercise	Answering questions from a list to share with the group (e.g., "what was the best day of your life")	1
Encourage group cohesion	Promote group solidarity, foster team unity	1
Practice interpersonal/communication skills	Enhance communicative proficiency and social competency	6
Resisting peer pressure	Rejecting social pressure and negative peer influence	2

Increase/decrease contact with peers	Increase contact with positive peers, decrease contact with negative peers	3
Enhance involvement in prosocial activities	Encourage youth to participate in positive extracurricular activities	3
Identify/describe relational functions	Discuss risk and protective factors in the family; the family's dynamics, resources and limitations	1
Conflict management and negotiation skills	Develop skills to use between family members or with other social peers	1
Improve family relationships	Enhance familial bonds, strengthen family ties, foster better connections between family members	4

Note. Common practice elements are bolded and underlined. The under categories are discrete practice elements. Total number of main common practice element categories = 11; total number of practice elements = 64; N = number of included studies that employ the given practice element
