# Supplementary material for: Identification of treatment elements for adolescents with callous unemotional traits: a systematic narrative review
Source: Child Adolesc Psychiatry Ment Health. 2024 Sep 3;18:110. doi: 10.1186/s13034-024-00792-2 (PMC11373131; doi:10.1186/s13034-024-00792-2)
Supplement: Supplementary file 3 — Supplementary Material 3 [file 13034_2024_792_MOESM3_ESM.pdf]

Title: Identification of Treatment Elements for Adolescents with Callous Unemotional Traits: A Systematic Narrative Review
Journal: Child and Adolescent Psychiatry and Mental Health
Authors: Pamela M. Waaler, Josefine Bergseth, Linda Vaskinn, Kristin Espenes, Thale Holtan, John Kjøbli, and Gunnar Bjørnebekk
Correspondence author: Pamela M. Waaler, Ph.D. candidate Department of Special Needs Education, University of Oslo; E-mail: p.m.waaler@isp.uio.no

Supplementary Material C

Process & Implementation Elements, Definitions and Frequencies

Process elements	Definition	N
Formal therapy	e.g., cognitive therapy, structural family therapy, multisystemic therapy	5
Treatment location	Home (family-based), school, neighborhood (community-based)	3
Psychoeducation	Intervention included diadic learning and/or teaching	3
Role-play	Role-play youth, parent, or intervention deliverer	4
Rotate role-play roles	Clients switch role-play roles; take on different roles	2
Homework	Homework assigned	2
Homework reviewed	Group discussion/individual review of homework	2
Group discussion	Discussions in a group setting	3
Modeling	Modeling by intervention deliverer, caregivers, peers	2
Practice exercises	Try out tasks in session	5
Important others	Caregivers receive intervention elements	5
Non-judgmental approach	Deliverers use a non-judgmental approach	1
Games	Board or computer game	1
Clips	Visual or auditory clips	1
Static stimuli	Photos of facial expressions, auditory clips, photos of static body postures	1
Anger thermometer	Pictorial representation of anger. Thermometers range from 10 degrees “mildly agitated” to “as angry as you have been in your life”	1
Index cards	Written scenarios, emotions, ambiguous situations on index cards	1
Support on demand	The individual receiving the intervention has to opportunity to request support if necessary	3
Feedback on performance	Individual receives feedback on performance and/or development	2
Peer feedback	Individual receives feedback form the other group members	2
Group performance	Perform scenarios for the group	2
Reward based	Group members receive some form of reinforcement for participation (e.g., point system)	2
External monitoring	Deliverer, researchers, teachers, peers, or others monitor (log, register, check) the subject’s performance or usage/dosage of elements	2
Regular support	The subject receives relevant support multiple times during the intervention without have to request it (e.g., follow-up calls, training)	3
Youth influence	Explicitly state that the youth’s personal opinions/preferences influence the delivery of the intervention	1
Family influence	Explicitly state that the family’s personal opinions/preferences influence the delivery of the intervention	4
Culturally sensitive	Clear indication that the intervention is culturally sensitive (e.g., using translators, adapt intervention to fit minority groups)	1
Multicomponent	Explicitly state that the intervention consists of different elements, themes, or core topics	4

Flexible/adaptive	Explicitly state that flexibility or the use of adaptations was allowed or encouraged	5
Individualized	Explicitly state that the intervention was tailored, adjusted, or adapted to individual needs or preferences	4
Pedagogical principles	Application of pedagogical theories and guidelines for teaching skills/elements	1
Engagement	Encouraging family/youth to engage in treatment	4
Social ecological	Intervention based on social-ecological principles (e.g., focus on interconnectedness, humans behave within context of system)	3
Strengths-based	Intervention focuses on the positive/client strengths	4
Refer to additional support	Put clients in contact appropriate services and other supports	4
Feedback from participants	Participants give feedback on how they experience the intervention	4
Implementation elements		
Therapist fidelity	Specific measurements, audio recordings, questionnaire to measure therapist fidelity to treatment/program	3
Boosters	Booster sessions provided after original training to enhance adherence	1
Supervision	Supervision of the delivery of the intervention to ensure adherence	4
Consultations	Consultations to discuss the implementation of the treatment protocol for adherence	3
Group training	Group training/coaching on the delivery of the intervention to ensure adherence	1
Participant satisfaction	Ratings of youth and/or caregiver's satisfaction with intervention as a measure of acceptability	3
Participant involvement	Participants rate level of information learned, enjoyment, and involvement as an indicator of acceptability	1
Participant acceptability	Participants rate their acceptability of the intervention	1
Participant appropriateness	Participants rate the quality of treatment, change, and effectiveness as an indicator of acceptability	1

Note. Total number of process elements = 36; total number of implementation elements = 9; N = number of included studies that employ the given process and implementation elements
