# Supplementary material for: Identification of treatment elements for adolescents with callous unemotional traits: a systematic narrative review
Source: Child Adolesc Psychiatry Ment Health. 2024 Sep 3;18:110. doi: 10.1186/s13034-024-00792-2 (PMC11373131; doi:10.1186/s13034-024-00792-2)
Supplement: Supplementary file 4 — Supplementary Material 4 [file 13034_2024_792_MOESM4_ESM.pdf]

Title: Identification of Treatment Elements for Adolescents with Callous Unemotional Traits: A Systematic Narrative Review
Journal: Child and Adolescent Psychiatry and Mental Health
Authors: Pamela M. Waaler, Josefine Bergseth, Linda Vaskinn, Kristin Espenes, Thale Holtan, John Kjøbli, and Gunnar Bjørnebekk
Correspondence author: Pamela M. Waaler, Ph.D. candidate Department of Special Needs Education, University of Oslo; E-mail: p.m.waaler@isp.uio.no

Supplementary Material D

Excluded Full Texts

Reference	Reason for Exclusion
Abell, M.L., Fraser, M. W., & Galinsky, M. J. (2001). Early intervention for aggressive behavior in childhood: A pilot study of a multi-component intervention with elementary school children and their families. *Journal of Family Social Work, 6*(4), 19-37	Children (0-11)
Abikoff, H., & Klein, R. G. (1992). Attention-deficit hyperactivity and conduct disorder: Comorbidity and implications for treatment. *Journal of Consulting and Clinical Psychology, 60*(6), 881-892.	Review
Acetti, L. & Nicolo, A. M (2012). Introduction. In R. E Piggle (Ed.) *Studi Psicoanalitici del Bambine e dell'Adolexcente*	Book/chapter
Adams, A. L., & Meaden, P. (2014). A 12-week comparison regarding symptom improvement in an urban university-based outpatient child psychiatry clinic. *American Journal of Therapeutics, 21*(1), 10-4	Not an RCT or quasi study
Adhikari, R. P., Upadhaya, N., Satinsky, E. N., Burkey, M. D., Kohrt, B. A., & Jordans, M. J. (2018). *Child and Adolescent Psychiatry and Mental Health, 12*(20), https://doi.org/10.1186/s13034-018-0226-3	Children (0-11)
Akin, B. A., McDonald, T. P., Yan, Y., Little, T., & Lang, K. (2019). Randomized Trial of PMTO in Foster Care: Six-Month Child Well-Being Outcomes. *Research on Social Work Practice, 29*(2), 206-222.	Children (0-11)
Alperin, A., Reddy, L. A., Glover, T. A., Bronstein, B., Wiggs, N. B., & Dudek, C. M. (2021). School-based interventions for middle school students with disruptive behaviors: A systematic review of components and methodology. *School Psychology Review*, DOI: 10.1080/2372966X.2021.1883996	Review
Alvarez, F. L., Chochol, M., Letelier, C. M., & Franco, I. N. (2019). Video-intervention therapy for caregivers of children hospitalized on an inpatient psychiatric unit: Results from a small randomized controlled trial. *Journal of the American Academy of Child and Adolescent Psychiatry, 58*(10), S265	Wrong outcomes
Anderson, V. R., Rubino, L. L., & McKenna, N. C. (2021). Family-based intervention for legal system-involved girls: A mixed methods evaluation. *American Journal of Community*, 67(1-2), 35-49.	Wrong outcomes

Anicama-Gomez, J. (1996). Effects of an aggression replacement training (ART) program for the prevention of violence in high risk minors. *Psicologia Contemporanea, 3*(2), 12-21.	Wrong outcomes
Ansari, A. A., Gouthro, S., Ahmad, K., & Steele, C. (1996). Hospital-based behavior modification program for adolescents: Evaluation and predictors of outcome. *Adolescence, 31*(122), 469-476.	Not an RCT or quasi study
Apsche, J. A., Bass, C. K., & Houston, M. (2008). Family mode deactivation therapy as a manualized cognitive behavioral therapy treatment. *International Journal of Behavioral Consultation & Therapy, 4*(2), 264-277.	Wrong population
Apsche, J. A., Bass, C. K., & Christopher, K. (2006). A treatment study of Mode Deactivation Therapy in an out patient community setting. *International Journal of Behavioral Consultation and Therapy, 2*(1), 85-93.	Wrong outcomes
Apsche, J. A., Bass, C. K., & Christopher, K. (2006). A review and empirical comparison of three treatments for adolescent males with conduct and personality disorder: Mode Deactivation Therapy, Cognitive Behavior Therapy and Social Skills Training. *International Journal of Behavioral Consultation and Therapy, 2*(3), 382-398.	Wrong outcomes
Apsche, J. A., Bass, C. K., Christopher, K., & Houston, M. A. (2006). A one year study of adolescent males with aggression and problems of conduct and personality: A comparison of MDT and DBT. *International Journal of Behavioral Consultation and Therapy, 2*(4), 544-552.	Wrong outcomes
Apsche, J. A., Bass, C. K., Jennings, J. L., Murphy, C. J., Hunter, L. A., & Siv, A. M. (2005). Empirical comparison of three treatments for adolescent males with physical and sexual aggression: Mode deactivation therapy, cognitive behavior therapy and social skills training. *International Journal of Behavioral Consultation and Therapy, 1*(2), 101-113.	Wrong outcomes
Apsche, J. A., Bass, C. K., Jennings, J. L., & Siv, A. M. (2005). A review and empirical comparison of two treatments for adolescent males with conduct and personality disorder: Mode Deactivation Therapy and Cognitive Behavior Therapy. *International Journal of Behavioral Consultation and Therapy, 1*(1), 27-45.	Wrong outcomes
Apsche, J. A., Bass, C. K., & Murphy, C. J. (2004). An empirical comparison of cognitive behavior therapy (CBT) and mode deactivation therapy (MDT) with adolescent males with conduct disorder and/or personality traits and sexually reactive behaviors. *The Behavior Analyst Today, 5*(4), 359-371.	Wrong outcomes
Apsche, J. A., Bass, C. K., & Murphy, C. J. (2004). A comparison of two treatment studies: CBT and MDT with adolescent male sex offenders with reactive conduct disorder and/or personality traits. *Journal of Early and Intensive Behavior, 1*(2), 179-190.	Wrong outcomes
Apsche, J. A., Bass, C. K., & Siv, A. M. (2005). A review and empirical comparison of three treatments for adolescent males with conduct and personality disorder: Mode Deactivation Therapy, Cognitive Behavior Therapy and Social Skills Training. *International Journal of Behavioral Consultation and Therapy, 1*(4), 371-381.	Wrong outcomes
Apsche, J. A., Bass, C. K., Siv, A. M., & Matteson, S. C. (2005). An empirical "real world" comparison of two treatments with aggressive adolescent males. *International Journal of Behavioral Consultation and Therapy, 1*(3), 239-251.	Wrong outcomes

Apsche, J. A., Bass, C. K., Zeiter, J., & Houston, M. A. (2008). Family mode deactivation therapy in a residential setting: Treating adolescents with conduct disorder and multi-axial diagnosis. *International Journal of Behavioral Consultation and Therapy, 4*(4), 328-339.	Wrong outcomes
Arkan, B., Güvenir, T., Ralph, A., & Day, J. (2020). The efficacy and acceptability of the Triple P: Positive Parenting Program with Turkish parents. *Journal of Child & Adolescent Psychiatric Nursing, 33*(3), 148-156.	Wrong outcomes
Armelius, B. A. & Andreassen, T. H. (2007). Cognitive-behavioral treatment for antisocial behavior in youth in residential treatment. *Cochrane Database of Systematic Reviews*. DOI: 10.1002/14651858.CD005650.pub2.	Review
Armstrong, T. A. (2002). The effect of environment on the behavior of youthful offenders: A randomized experiment. *Journal of Criminal Justice, 30*(1), 19-28.	Adults (19+)
Arnold, L., Gadow, K. D., Farmer, C. A., Findling, R. L., Bukstein, O., Molina, B. S., Brown, N. V., Li, X., Rundberg-Rivera, E., Bangalore, S., Buchan-Page, K., Hurt, E. A., Rice, R., McNamara, N. K., & Aman, M. G. (2015). Comorbid anxiety and social avoidance in treatment of severe childhood aggression: Response to adding risperidone to stimulant and parent training; mediation of disruptive symptom response. *Journal of Child and Adolscent Psychopharmacology, 25*(3), 203-212.	Children (0-11)
Asscher, J. J., Dekovic, M., Manders, W. A., van der Laan, P. H., Prins, P. J. M., & Dutch M. S. T. Cost-Effectiveness Study (2013). A randomized controlled trial of the effectiveness of multisystemic therapy in the Netherlands: post-treatment changes and moderator effects. *Journal of Experimental Criminology, 9*(2), 169-187.	Wrong outcomes
Asscher, J. J., Dekovic, M., Van den Akker, A. L., Prins, P. J. M., & Van der Laan, P. H. (2018). Do extremely violent juveniles respond differently to treatment? *International Journal of Offender Therapy and Comparative Criminology, 62*(4), 958-977.	Wrong outcomes
Atashak, S. (2014). Efficacy of management training in conduct disorder in adolescents. *World Health Organization International Clinical Trials Registry Platform.* https://trialsearch.who.int/Trial2.aspx?TrialID=IRCT2013121110512N3	Wrong outcomes
Azrin, N. H., Donohue, B., Teichner, G. A., Crum, T., Howell, J., & DeCato, L. A. (2001). A controlled evaluation and description of individual-cognitive problem solving and family-behavior therapies in dually-diagnosed conduct-disordered and substance-dependent youth. *Journal of Child & Adolescent Substance Abuse, 11*(1), 1-43.	Wrong outcomes
Backer, H. S., Miller, A. L., & Van Den Bosch, L. M. C. (2009). Dialectical behaviour therapy for adolescents. *Tijdschrift voor Psychiatrie, 51*(1), 31-41.	Review
Bakker, M., Greven, C., Buitelaar, J., & Glennon, J. (2017). Practitioner review: Psychological treatments for children and adolescents with conduct disorder problems-A systematic review and meta-analysis. *Journal of Child Psychology and Psychiatry, 58*(1), 4-18.	Review
Bamelis, L. L., Evers, S. M., & Arntz, A. (2012). Design of a multicentered randomized controlled trial on the clinical and cost effectiveness of schema therapy for personality disorders. *BMC Public Health, 12*(75), https://doi.org/10.1186/1471-2458-12-75	Adults (19+)
Barterian, J. A., Arnold, L., Brown, N. V., Farmer, C. A., Williams, C., Findling, R. L., Kolko, D. J., Bukstein, O. G., Molina, B. S., Townsend, L., & Aman, M. G.	Children (0-11)

(2017). Clinical implications from the Treatment of Severe Childhood Aggression (TOSCA) study: A re-analysis and integration of findings. *Journal of the American Academy of Child & Adolescent Psychiatry, 56*(12), 1026-1033.	
Baruch, G., Vrouva, I., & Wells, C. (2011). Outcome findings from a parent training programme for young people with conduct problems. *Child and Adolescent Mental Health, 16*(1), 47-54.	Not an RCT or quasi study
Battagliese, G., Caccetta, M., Luppino, O. I., Baglioni, C., Cardi, V., Mancini, F., & Buonanno, C. (2015). Cognitive-behavioral therapy for externalizing disorders: A meta-analysis of treatment effectiveness. *Behaviour Research and Therapy*, 60-71.	Review
Baumel, A., Pawar, A., Kane, J. M., & Correll, C. U. (2016). Digital parent training for children with disruptive behaviors: Systematic review and meta-analysis of randomized trials. *Journal of Child and Adolescent Psychopharmacology, 26*(8), 740-749.	Review
Bayles, C., Blossom, P., & Apsche, J. (2014). A brief review and update of mode deactivation therapy. *International Journal of Behavioral Consultation and Therapy, 9*(1), 46-48.	Review
Bazargan, Y., & Pakdaman, S. (2016). The effectiveness of art therapy in reducing internalizing and externalizing problems of female adolescents. *Archives of Iranian Medicine (AIM), 19*(1), 37-42.	Wrong outcomes
Beard, K. Y. & Sugai, G. (2004). First Step to Success: An early intervention for elementary children at risk for antisocial behavior. *Behavioral Disorders, 29*(4), 396-409.	Children (0-11)
Beaumont, C., Royer, E., Bertrand, R., & Bowen, F. (2005). The effects of an adapted peer-mediation program in 6 students with conduct disorder. *Canadian Journal of Behavioral Science, 37*(3), 198-210.	Children (0-11)
Beelmann, A., Arnold, L. S., & Hercher, J. (2023). Parent training programs for preventing and treating antisocial behavior in children and adolescents: A comprehensive meta-analysis of international studies. *Aggression and Violent Behaivor, 68,* 1-12.	Review
Bennett, D. S., & Gibbons, T. A. (2000). Efficacy of child cognitive-behavioral interventions for antisocial behavior: A meta-analysis. *Child & Family Behavior Therapy*, *22*(1), 1-15.	Review
Berg, E. L., Gooch, M., Feldmann, L., Knight, B., & Verlaine, J. (2017). Equine-assisted psychotherapy treatment in a residential childcare facility [Supplemental material]. *Journal of Equine Veterinary Science, 52,* 114-126.	Ongoing study with no publication
Berry, V., Little, M., Axford, N., & Cusick, G. R. (2009). An evaluation of youth at risk's coaching for communities programme. *Howard Journal of Criminal Justice, 48*(1), 60-75.	Wrong outcomes
Besier, T., Fegert, J. M., & Goldbeck, L. (2009). Evaluation of psychiatric liaison-services for adolescents in residential group homes. *European Psychiatry: the Journal of the Association of European, 24*(7), 483-489.	Wrong outcomes
Biehal, N., Dixon, J., Parry, E., Sinclair I., Green, N., Roberts, C., Kay, C., Rothwell, J., Kapadia, D. & Roby, A. (2012). *The care placements evaluation (CaPE) evaluation of multidimensional treatment foster care for adolescents (MTFC-A).* Department for Education.	Wrong outcomes

Bierman, K. L., Coie, J. D., Dodge, K. A., Foster, E., Greenberg, M. T., Lochman, J. E., McMahon, R. J. & Pinderhughes, E. E. (2007). Fast track randomized controlled trial to prevent externalizing psychiatric disorders: Findings from grades 3 to 9. *Journal of the American Academy of Child & Adolescent Psychiatry, 46*(10), 1250-1262.	Children (0-11)
Boege, I., Mayer, L., Muche, R., Corpus, N., Schepker, R., & Fegert, J. M. (2015). Home treatment - especially effective among boys with externalizing disorders? Age- and sex-specific effectiveness of home treatment for internalizing and externalizing disorders. *Zeitschrift fur Kinder-und Jugendpsychiatrie und Psychotherapie, 43*(3), 161-171.	Wrong outcomes
Boege, I., Reck, N., Schepker, R., & Fegert, J. M. (2014). Interval treatment instead of disciplinary discharge. Effectiveness of a structured psychotherapeutic treatment program for conduct disorder. *Psychotherapeut, 59*(5), 363-370.	Wrong outcomes
Bogels, S., Hoogstad, B., van Dun, L., de Schutter, S., & Restifo, K. (2008). Mindfulness training for adolescents with externalizing disorders and their parents. *Behavioural and Cognitive Psychotherapy, 36*(2), 193-209.	Wrong outcomes
Boldrini, T., Ghiandoni, V., Mancinelli, E., Salcuni, S. & Solmi, M. (2023). Systematic review and meta-analysis: Psychosocial treatments for disruptive behavior symptoms and disorders in adolescence. *Journal of the American Academy of Child & Asolescent Psychiatry, 62*(2), 169-189.	Review
Boland Hemmatan, K. (2020). Effects of Aggression Replacement Training (ART) on anger. *Iranian Registry of Clinical Trials.* https://www.irct.ir/trial/44006	Wrong outcomes
Borduin, C. M., Schaeffer, C. M., & Heiblum, N. (2009). A randomized clinical trial of multisystemic therapy with juvenile sexual offenders: effects on youth social ecology and criminal activity. *Journal of Consulting and Clinical Psychology, 77*(1), 26-37.	Wrong outcomes
Borduin, C. M., Mann, B. J., Cone, L. T., Henggeler, S. W., Fucci, B. R., Blaske, D. M., & Williams, R. A. (1995). Multisystemic treatment of serious juvenile offenders: Long-term prevention of criminality and violence. *Journal of Consulting and Clinical Psychology, 63*(4), 569-578.	Wrong outcomes
Bozzatello, P., & Bellino, S. (2016). Combined therapy with interpersonal psychotherapy adapted for borderline personality disorder: a two-years follow-up. *Psychiatry Research, 240,* 151-156.	Adults (19+)
Brannstrom, L., Kaunitz, C., Andershed, A. K., South, S., & Smedslund, G. (2016). Aggression replacement training (ART) for reducing antisocial behavior in adolescents and adults: A systematic review. *Aggression and Violent Behavior,* 30-41.	Review
Brathwaite, R., Ssewamala, F. M., Sensoy B. O., McKay, M. M., Neilands, T. B., Namatovu, P., Kiyingi, J., Zmachinski, L., Nabayinda, J., Huang, K. Y., Kivumbi, A., Bhana, A., Mwebembezi, A., Petersen, I., & Hoagwood, K. (2022). The longitudinal impact of an evidence-based multiple family group intervention (Amaka Amasanyufu) on oppositional defiant disorder and impaired functioning among children in Uganda: analysis of a cluster randomized trial from the SMART Africa-Uganda scale-up study (2016–2022). *Journal of Child Psychology & Psychiatry, 63*(11), 1252-1260.	Children (0-11)
Brazao, N., da Motta, C., Rijo, D., Salvador, M. D., Pinto-Gouveia, J., & Ramos, J. (2015). Clinical change in anger, shame, and paranoia after a structured cognitive-	Adults (19+)

behavioral group program: Early findings from a randomized trial with male prison inmates. *Journal of Experimental Criminology, 11*(2), 217-236.	
Breider, S., de Bildt, A., Nauta, M. H., Hoekstra, P. J., & van den Hoofdakker, B. J. (2019). Self-directed or therapist-led parent training for children with attention deficit hyperactivity disorder? A randomized controlled non-inferiority pilot trial. *Internet Interventions, 18,* doi:10.1016/j.invent.2019.100262	Children (0-11)
Brestan, E. V., & Eyberg, S. M. (1998). Effective psychosocial treatments of conduct-disordered children and adolescents: 29 years, 82 studies, and 5,272 kids. *Journal of Clinical Child Psychology, 27*(2), 180-189.	Review
Brody, G. H., Chen, Y. F., Kogan, S. M., Yu, T., Molgaard, V. K., DiClemente, R. J., & Wingood, G. M. (2012). Family-centered program deters substance use, conduct problems, and depressive symptoms in black adolescents. *Pediatrics, 129*(1), 108-115.	Wrong outcomes
Bruns, E. J., & Burchard, J. D. (2000) Impact of respite care services for families with children experiencing emotional and behavioral problems. *Children's Services: Social Policy, Research, & Practice, 3*(1), 39-61.	Children (0-11)
Burke, K., Brennan, L., & Cann, W. (2012). Promoting protective factors for young adolescents: ABCD Parenting Young Adolescents Program randomized controlled trial. *Journal of Adolescence, 35*(5), 1315-1328.	Children (0-11)
Burkey, M. D., Hosein, M., Morton, I., Purgato, M., Adi, A., Kurzrok, M., Kohrt, B. A., & Tol, W. A. (2018). Psychosocial interventions for disruptive behaviour problems in children in low- and middle-income countries: a systematic review and meta-analysis. *Journal of Child Psychology & Psychiatry & Allied Disciplines, 59*(9), 982-993.	Review
Bustamante, A. M. (2000). *Outcome of a standardized strategic family intervention for disruptive behavior disorders: A multisite randomized trial* [Unpublished doctoral dissertation]. Adelphi University.	Wrong outcomes
Bustos, A. R. (2022). *Examining the effectiveness of the parent project on callous-unemotional traits among a sample of adolescents on probation* [Unpublished doctoral dissertation].	Could not contact author
Butler, S., Baruch, G., Hickey, N., & Fonagy, P. (2012). A randomized controlled trial of multisystemic therapy and a statutory therapeutic intervention for young offenders: Corrigendum. *Journal of the American Academy of Child & Adolescent Psychiatry, 51*(3), 337.	Duplicate
Cabiya, J., Padilla-Cotto, L., Gonzalez, K., Sanchez-Cestero, J., Martinez-Taboas, A., & Sayers, S. (2008). Effectiveness of a cognitive-behavioral intervention for Puerto Rican children. *Revista Interamericana de Psicologia, 42*(2), 195-202.	Children (0-11)
Caldwell, M. F. (2011). Treatment-related changes in behavioral outcomes of psychopathy facets in adolescent offenders. *Law and Human Behavior, 35*(4), 275-287.	CU and/or antisocial behavior not measured at post
Caldwell, M. F., McCormick, D. J., Umstead, D., & Van Rybroek, G. J. (2007). Evidence of treatment progress and therapeutic outcomes among adolescents with psychopathic features. *Criminal Justice and Behavior, 34*(5), 573-587.	Not an RCT or quasi study
Caldwell, M. F., McCormick, D., Wolfe, J., & Umstead, D. (2012). Treatment-related changes in psychopathy features and behavior in adolescent offenders. *Criminal Justice and Behavior, 39*(2), 144-155.	Not an RCT or quasi study

Caldwell, M., Skeem, J., Salekin, R., & Van Rybroek, G. (2006). Treatment response of adolescent offenders with psychopathy features: A 2-year follow-up. *Criminal Justice and Behavior, 33*(5), 571-596.	CU and/or antisocial behavior not measured at post
Cardwell, S. M., Mazerolle, L., & Piquero, A. R. (2020). Parental attachment and truant rationalizations of antisocial behavior: findings from a randomized controlled trial, *Journal of Crime & Justice, 43*(3), 263-281.	Wrong outcomes
Carnerio, M. C. B., Kaio, C. H. & Doria, G. M. S. (2019). 2.9 Motivational interviewing with a Brazilian sample of adolescent inpatients with substance use disorder: Intervention and outcomes. *Journal of the American Academy of Child and Adolescent Psychiatry, 58*(10), DOI: 10.1016/j.jaac.2019.08.101	Symposia/abstract for a conference
Carr, A. (2014). The evidence base for family therapy and systemic interventions for child-focused problems. *Journal of Family Therapy, 36*(2), 107-157.	Review
Carr, A. (2009). The effectiveness of family therapy and systemic interventions for child-focused problems. *Journal of Family Therapy, 31*(1), 3-45.	Review
Carr, A., Hartnett, D., Brosnan, E., & Sharry, J. (2017).Parents plus systemic, solution-focused parent training programs: Description, review of the evidence base, and meta-analysis. *Family Process, 56*(3), 652-668.	Review
Cassells, C., Carr, A., Forrest, M., Fry, J., Beirne, F., Casey, T., & Rooney, B. (2015). Positive systemic practice: A controlled trial of family therapy for adolescent emotional and behavioural problems in Ireland. *Journal of Family Therapy, 37*(4), 429-449.	Wrong outcomes
Castellanos, N. & Conrod, P. (2006). Brief interventions targeting personality risk factors for adolescent substance misuse reduce depression, panic and risk-taking behaviours. *Journal of Mental Health (Abingdon, England, 15*(6), 645-658.	Wrong outcomes
Chacko, A., Isham, A., Cleek, A. F., & McKay, M. M. (2016). Using mobile health technology to improve behavioral skill implementation through homework in evidence-based parenting intervention for disruptive behavior disorders in youth: Study protocol for intervention development and evaluation. *Pilot & Feasibility Studies, 2*, 57.	Protocol
Chamberlain, P., Price, J., Leve, L. D., Laurent, H., Landsverk, J. A., & Reid, J. B. (2008). Prevention of behavior problems for children in foster care: outcomes and mediation effects. *Prevention Science, 9*(1), 17-27.	Children (0-11)
Chorpita, B. F., Daleiden, E. L., Park, A. L., Ward, A. M., Levy, M. C., Cromley, T., Chiu, A. W., Letamendi, A. M., Tsai, K. H., & Krull, J. L. (2017). Child STEPs in California: A cluster randomized effectiveness trial comparing modular treatment with community implemented treatment for youth with anxiety, depression, conduct problems, or traumatic stress. *Journal of Consulting and Clinical Psychology, 85(*1), 13-25.	Children (0-11)
Chorpita, B. F., Weisz, J. R., Daleiden, E. L., Schoenwald, S. K., Palinkas, L. A., Miranda, J., Higa-McMillan, C. K., Nakamura, B. J., Austin, A., Borntrager, C. F., Ward, A., Wells, K. C., & Gibbons, R. D. (2013). Long-term outcomes for the Child STEPs randomized effectiveness trial: A comparison of modular and standard treatment designs with usual care. *Journal of Consulting and Clinical Psychology, 81*(6), 999-1009.	Children (0-11)

Ciocanel, O., Power, K., Eriksen, A., & Gillings, K. (2017). Effectiveness of positive youth development interventions: A meta-analysis of randomized controlled trials. *Journal of Youth and Adolescence, 46*(3), 483-504.	Review
Clair-Michaud, M., Martin, R. A., Stein, L. A. R., Bassett, S., Lebeau, R., & Golembeske, C. (2016). The impact of motivational interviewing on delinquent behaviors in incarcerated adolescents. *Journal of Substance Abuse Treatment, 65*, 13-19.	Wrong outcomes
Clark, H. B., Prange, M. E., Lee, B., Boyd, L., McDonald, B. A., & Stewart, E. S. (1994). Improving adjustment outcomes for foster children with emotional and behavioral disorders: Early findings from a controlled study on individualized services. *Journal of Emotional and Behavioral Disorders, 2*(4), 207-218.	Wrong outcomes
Coatsworth, J. D., Santisteban, D. A., McBride, C. K., & Szapocznik, J. (2001). Brief strategic family therapy versus community control: engagement, retention, and an exploration of the moderating role of adolescent symptom severity. *Family Process, 40*(3), 313-32.	Wrong outcomes
Coleman, M., Pfeiffer, S., & Oakland, T. (1992). Aggression replacement training with behaviorally disordered adolescents. *Behavioral Disorders, 18*(1), 54-66.	CU and/or antisocial behavior not measured at post
Colins, O. F., Andershed, H., Salekin, R. T., & Fanti, K. A. (2018). Comparing different approaches for subtyping children with conduct problems: Callous-unemotional traits only versus the multidimensional psychopathy construct. *Journal of Psychopathology & Behavioral Assessment*, *40*(1), 6-15.	No treatment tested/given
Colmant, S. A. & Merta, R. J. (1999). Using the sweat lodge ceremony as group therapy for Navajo youth. *Journal for Specialists in Group Work*, *24*(1), 55-73.	Not an RCT or quasi study
Connell, A. M., Dishion, T. J., Yasui, M., & Kavanagh, K. (2007). An adaptive approach to family intervention: Linking engagement in family-centered intervention to reductions in adolescent problem behavior. *Journal of Consulting and Clinical Psychology*, *75*(4), 568-579.	Wrong population
Conniff, K. M., Scarlett, J. M., Goodman, S., & Appel, L. D. (2005). Effects of a pet visitation program on the behavior and emotional state of adjudicated female adolescents. *Anthrozoos, 18*(4), 379-395.	Wrong intervention
Connor, D. F., Carlson, G. A., Chang, K. D., Daniolos, P. T., Ferziger, R., Findling, R. L., Hutchinson, J. G., Malone, R. P., Halperin, J. M., Plattner, B., Post, R. M., Reynolds, D. L., Rogers, K. M., Saxena, K., & Steiner, H. (2006). Juvenile maladaptive aggression: A review of prevention, treatment, and service configuration and a proposed research agenda. *The Journal of Clinical Psychiatry, 67*(5), 808-820.	Review
Constantino, J. N. (2016). Supplementing early head start with parenting education. *Journal of the American Academy of Child and Adolescent Psychiatry, 55*(10.1, Suppl.), S275-S276.	Supplement with no publication
Copping, V. E., Warling, D. L., Benner, D. G. & Woodside, D. W. (2001). A child trauma treatment pilot study. *Journal of Child and Family Studies, 10*(4), 467-475.	Children (0-11)
Costin, J., Lichte, C., Hill-Smith, A., Vance, A., & Luk, E. (2004). Parent group treatments for children with Oppositional Defiant Disorder. *Australian e-Journal for the Advancement of Mental Health, 3*(1), No Pagination Specified.	Children (0-11)

Cottrell, D., & Boston, P. (2002). Practitioner review: The effectiveness of systemic family therapy for children and adolescents. *Journal of Child Psychology and Psychiatry, 43*(5), 573-586.	Review
Crombach, A. & Elbert, T. (2015). Controlling offensive behavior using narrative exposure therapy: A randomized controlled trial of former street children. *Clinical Psychological Science, 3*(2), 270-282.	Wrong outcomes
Cropp, C., Streeck-Fischer, A., & Leichsenring, F. (2012). Evaluation of inpatient psychodynamic psychotherapy for adolescents who suffer mixed disorders of conduct and emotions (F92, ICD-10). *Adolescent psychiatry, 2*(1), 101.	Wrong outcomes
Cropp, C., Taubner, S., Salzer, S., & Streeck-Fischer, A. (2019). Psychodynamic psychotherapy with severely disturbed adolescents: Changes in reflective functioning. *Journal of Infant, Child & Adolescent Psychotherapy, 18*(3), 263-273.	Wrong outcomes
D'Oosterlinck, F., Goethals, I., Boekaert, E., Schuyten, G., & De Maeyer, J. (2008). Implementation and effect of life space crisis intervention in special schools with residential treatment for students with emotional and behavioral disorders (EBD). *Psychiatric Quarterly, 79*(1), 65-79.	Wrong outcomes
Damen, H., Veerman, J. W., Vermulst, A. A., Pagée, R., Nieuwhoff, R., & Scholte, R. H. J. (2019). Parental empowerment and child behavioural problems during youth care involvement. *Child & Family Social Work, 24*(4), 467-476.	Wrong outcomes
Danko, C. M., Garbacz, L. L., & Budd, K. S. (2016). Outcomes of parent-child interaction therapy in an urban community clinic: A comparison of treatment completers and dropouts. *Children and Youth Services Review*, 42-51.	Not an RCT or quasi study
Davis, M., Sheidow, A. J., & McCart, M. R. (2015). Reducing recidivism and symptoms in emerging adults with serious mental health conditions and justice system involvement. *The Journal of Behavioral Health Services & Research, 42*(2), 172-190.	Not an RCT or quasi study
de la Cruz, L. F., Simonoff, E., McGough, J. J., Halperin, J. M., Arnold, L., & Stringaris, A. (2015). Treatment of children with attention-deficit/hyperactivity disorder (ADHD) and irritability: Results from the multimodal treatment study of children with ADHD (MTA). J*ournal of the American Academy of Child & Adolescent Psychiatry, 54*(1), 62-70.	Children (0-11)
de Vries, S. L. A., Hoeve, M., Wibbelink, C. J. M., Asscher, J. J., & Stams, G. J. J. M. (2017). A randomized controlled trial of the effectiveness of the youth crime prevention program 'New Perspectives' (NP): Post-treatment changes and moderator effects. *Children & Youth Services Review, 82*, 413-426.	Wrong outcomes
Dedousis-Wallace, A., Drysdale, S. A., McAloon, J. & Ollendick, T. H. (2020). Parental and familial predictors and moderators of parent management treatment programs for conduct problems in youth. *Clinical Child and Family Psychology Review*, No Pagination Specified.	Review
DeGarmo, D. S., Chamberlain, P., Leve, L. D., & Price, J. (2009). Foster parent intervention engagement moderating child behavior problems and placement disruption. *Research on Social Work Practice, 19*(4), 423-433.	Wrong outcomes
Dembo, R., Wareham, J., Poythress, N. G., Cook, B., & Schmeidler, J. (2006). The impact of arbitration intervention services on youth recidivism: One-year follow-up *Journal of Offender Rehabilitation, 43*(4), 95-131.	Wrong intervention

del Valle, P., Kelley, S. L., & Seoanes, J. E. (2001). The "oppositional defiant" and "conduct disorder" child: A brief review of etiology, assessment, and treatment. *Behavioral Development Bulletin, 10*(1), 36-41.	Review
Diamond, G. & Josephson, A. (2005). Family-based treatment research: A 10-year update. *Journal of the American Academy of Child & Adolescent Psychiatry, 44*(9), 872-887.	Review
Dishion, T. J., & Andrews, D. W. (1995). Preventing escalation in problem behaviors with high-risk young adolescents: Immediate and 1-year outcomes. *Journal of Consulting & Clinical Psychology, 63*(4), 538-548.	Wrong outcomes
Dodge, K. A., & McCourt, S. N. (2010). Translating models of antisocial behavioral development into efficacious intervention policy to prevent adolescent violence. *Developmental Psychobiology, 52*(3), 277-285.	Children (0-11)
Doepfner, M., Goertz-Dorten, A., Benesch, C., & Hautmann, C. (2013). Efficacy of the treatment program for children with aggressive behaviour -a randomized controlled trial with an active control group. *European Child and Adolescent Psychiatry, 1*(Suppl.), S172.	Supplement with no publication
Donohue, B., Azrin, N. H., Lawson, H., Friedlander, J., Teichner, G., & Rindsberg, J. (1998). Improving initial session attendance of substance abusing and conduct disordered adolescents: A controlled study. *Journal of Child & Adolescent Substance Abuse, 8(*1), 1-13.	Wrong outcomes
Dopp, A. R., Borduin, C. M., White, M. H., II, & Kuppens, S. (2017). Family-based treatments for serious juvenile offenders: A multilevel meta-analysis. *Journal of Consulting and Clinical Psychology, 85*(4), 335-354.	Review
Dretzke, J. Davenport, C., Frew, E., Barlow, J., Stewart-Brown, S., Bayliss, S., Taylor, R. S., Sandercock, J., & Hyde, C. (2009). The clinical effectiveness of different parenting programmes for children with conduct problems: A systematic review of randomised controlled trials. *Child and Adolescent Psychiatry and Mental Health, 3*(7)	Review
Dretzke, J. Davenport, C., Frew, E., Barlow, J., Stewart-Brown, S., Bayliss, S., Taylor, R. S., Sandercock, J., & Hyde, C. (2009). The clinical effectiveness of different parenting programmes for children with conduct problems: A systematic review of randomised controlled trials. *Child and Adolescent Psychiatry and Mental Health, 3*(7)	Duplicate
Earls, M. K. (2011). *The play factor: Effect of Social Skills Group Play Therapy on adolescent African-American males* [Unpublished doctoral dissertation]. Texas Southern University.	Wrong outcomes
Earls, M. K. (2011). *The play factor: Effect of Social Skills Group Play Therapy on adolescent African-American males* [Unpublished doctoral dissertation]. Texas Southern University.	Duplicate
Eber, L., Osuch, R., & Redditt, C. A. (1996). School-based applications of the wraparound process: Early results on service provision and student outcomes. *Journal of Child and Family Studies, 5*(1), 83-99.	Wrong outcomes
Edalati, H., Afzali, M. H., Castellanos-Ryan, N., & Conrod, P. J. (2019). The effect of contextual risk factors on the effectiveness of brief personality-targeted interventions for adolescent alcohol use and misuse: A cluster-randomized trial. *Alcoholism: Clinical & Experimental Research, 43*(5), 997-1006.	Wrong outcomes

Eddy, J., & Chamberlain, P. (2000). Family management and deviant peer association as mediators of the impact of treatment condition on youth antisocial behavior. *Journal of Consulting and Clinical Psychology, 68*(5), 857-863.	Wrong outcomes
Eiraldi, R., Power, T. J., Schwartz, B. S., Keiffer, J. N., McCurdy, B. L., Mathen, M., & Jawad, A. F. (2016). Examining effectiveness of group cognitive-behavioral therapy for externalizing and internalizing disorders in urban schools. *Behavior Modification, 40*(4), 611-639.	Wrong outcomes
Emerson Coons, S. R. (1998). *Shame-reduction group therapy with conduct disordered adolescents: A self-psychological approach to understanding aggression and its treatment* [Unpublished doctoral dissertation]. Fuller Theological Seminary School of Psychology.	Wrong outcomes
Enebrink, P. (2015). Coping power program and parent management training for families with children with conduct problems. *World Health Organization International Clinical Trials Registry Platform.* http://www.who.int/trialsearch/Trial2.aspx?TrialID=ISRCTN10834473	Children (0-11)
Ensink, K., Robertson, B. A., Zissis, C., Leger, P., & De Jager, W. (1997). Conduct disorder among children in an informal settlement. Evaluation of an intervention programme. *South African Medical Journal, 87*(11), 1533-1537.	Wrong outcomes
Epstein, R. A., Fonnesbeck, C., Potter, S., Rizzone, K. H., & McPheeters, M. (2015). Psychosocial interventions for child disruptive behaviors: A meta-analysis. *Pediatrics, 136*(5), 947-960.	Review
Ercan, E. S., Ardic, U. A., Kutlu, A., & Durak, S. (2014). No beneficial effects of adding parent training to methylphenidate treatment for ADHD + ODD/CD children: A 1-year prospective follow-up study. *Journal of Attention Disorders, 18*(2), 145-157.	Children (0-11)
Ercan, E. S., Varan, A., & Deniz, U. (2005). Effects of combined treatment on Turkish children diagnosed with attention-deficit/hyperactivity disorder: A preliminary report. *Journal of Child and Adolescent Psychopharmacology, 15*(2), 203-219.	Children (0-11)
Erford, B. T., Bardhoshi, G., Ross, M., Gunther, C., & Duncan, K. (2017). Meta-analysis of counseling outcomes for youth with conduct disorders. *Journal of Counseling & Development, 95*(1), 35-44.	Review
Erford, B. T., Paul, L. E., Oncken, C., Kress, V. E., & Erford, M. R. (2014). Counseling outcomes for youth with oppositional behavior: A meta-analysis. *Journal of Counseling & Development, 92*(1), 13-24.	Review
Etscheidt, S. (1991). Reducing aggressive behavior and improving self-control: A cognitive-behavioral training program for behaviorally disordered adolescents. *Behavioral Disorders, 16*(2), 107-115.	Wrong outcomes
Family Works Incorporated. (2013). Online Parent Training for Children With Behavior Disorders. *U.S National Library of Medicine Clinical Trials.* https://clinicaltrials.gov/ct2/show/NCT01861158	Wrong outcomes
Farmer, C. A., Arnold, L. E., Bukstein, O. G., Findling, R. L., Gadow, K. D., Li, X., Butter, E. M., & Aman, M. G. (2011). The treatment of severe child aggression (TOSCA) study: Design challenges. *Child and Adolescent Psychiatry and Mental Health, 5*(36)	Children (0-11)
Farmer, C. A., Brown, N. V., Gadow, K. D., Arnold, L., Kolko, D. G., Findling, R. L., Molina, B. S., Buchan-Page, K. A., Rice, R. R. Jr., Bangalore, S. S., Bukstein, O.,	Children (0-11)

Rundberg-Rivera, E., McNamara, N., & Aman, M. G. (2015). Comorbid symptomatology moderates response to risperidone, stimulant, and parent training in children with severe aggression, disruptive behavior disorder, and attention-deficit/hyperactivity disorder. *Journal of Child and Adolescent Psychopharmacology, 25*(3), 213-224.	
Farmer, E. M. Z., Burns, B. J., Wagner, H. R., Murray, M., & Southerland, D. G. (2010). Enhancing "usual practice" treatment foster care: Findings from a randomized trial on improving youths' outcomes. *Psychiatric Services, 61*(6), 555-561.	Wrong outcomes
Farrington, D. P. (2005). The importance of child and adolescent psychopathy. *Journal of Abnormal Child Psychology, 33*(4), 489-497.	Review
Findling, R. L., Townsend, L., Brown, N. V., Arnold, L., Gadow, K. D., Kolko, D. J., McNamara, N. K., Gary, D. S., Kaplin, D. B., Farmer, C. A., Kipp, H., Williams, C., Butter, E. M., Bukstein, O. G., Rice, R. Jr., Buchan-Page, K., Molina, B. S., & Aman, M. G. (2017). The treatment of severe childhood aggression study: 12 weeks of extended, blinded treatment in clinical responders. *Journal of Child and Adolescent Psychopharmacology, 27*(1), 52-65.	Children (0-11)
Fitzgerald, B. (1991). *An evaluation of a cognitive-behavioral intervention with severely emotionally handicapped seventh-grade boys* [Unpublished doctoral dissertation]. Pace University.	Wrong outcomes
Florean, I. S., Dobrean, A., Pasarelu, C. R., Georgescu, R. D., & Milea, I. (2020). The efficacy of internet-based parenting programs for children and adolescents with behavior problems: A meta-analysis of randomized clinical trials. *Clinical Child and Family Psychology Review, 23*(4), 510-528.	Review
Fonagy, P., Butler, S., Baruch, G., Byford, S., Seto, M. C., Wason, J., Wells, C., Greisbach, J., Ellison, R., & Simes, E. (2015). Evaluation of multisystemic therapy pilot services in Services for Teens Engaging in Problem Sexual Behaviour (STEPS-B): Study protocol for a randomized controlled trial. *Trials*, 16(492). DOI 10.1186/s13063-015-1017-2	Ongoing study with no publication
Fonagy, P., Butler, S., Cottrell, D., Scott, S., Pilling, S., Eisler, I., Fuggle, P., Kraam, A., Byford, S., Wason, J., Smith, J. A., Anokhina, A., Ellison, R., Simes, E., Ganguli, P., Allison, E., & Goodyer, I. M. (2020). The impact of different patterns of care on the long-term outcome of adolescent conduct disorder: A mixed methods study comparing multisystemic therapy (MST) and management as usual (MAU). *NIHR Journals Library. Health Services and Delivery Research*	Duplicate
Fonagy, P., Butler, S., Goodyer, I., Cottrell, D., Scott, S., Pilling, S., Eisler, I., Fuggle, P., Kraam, A., Byford, S., Wason, J., & Haley, R. Evaluation of multisystemic therapy pilot services in the Systemic Therapy for At Risk Teens (START) trial: Study protocol for a randomised controlled trial. *Trials, 14*(265). http://www.trialsjournal.com/content/14/1/265	Protocol
Fonagy, P., & Luyten, P. (2018). Conduct problems in youth and the RDoC approach: A developmental, evolutionary-based view. *Clinical Psychology Review 64*, 57-76.	Not an RCT or quasi study
Fonagy, P., Butler, S., Cottrell, D., Scott, S., Pilling, S., Eisler, I., Fuggle, P., Kraam, A., Byford, S., Wason, J., Smith, J. A., Anokhina, A., Ellison, R., Simes, E., Ganguli, P., Allison, E., & Goodyer, I. M. (2020). Multisystemic therapy versus management as usual in the treatment of adolescent antisocial behaviour (START): 5-year follow-	Duplicate

up of a pragmatic, randomised, controlled, superiority trial. *The Lancet Psychiatry 7*(5), 420-430.	
Fonagy, P., Butler, S., Cottrell, D., Scott, S., Pilling, S., Eisler, I., Fuggle, P., Kraam, A., Byford, S., Wason, J., Smith, J. A., Anokhina, A., Ellison, R., Simes, E., Ganguli, P., Allison, E., & Goodyer, I. M. (2020). Multisystemic therapy compared with management as usual for adolescents at risk of offending: The START II RCT. *Health Services and Delivery Research, 8*(23), DOI: 10.3310/hsdr08230	Duplicate
Fonagy, P., Twemlow, S. W., Vernberg, E. M., Nelson, J. M., Dill, E. J., Little, T. D., & Sargent, J. A. (2009). A cluster randomized controlled trial of child-focused psychiatric consultation and a school systems-focused intervention to reduce aggression. *Journal of Child Psychology and Psychiatry, 50*(5), 607-616.	Children (0-11)
Fongaro, E., Picot, M. C., Stringaris, A., Belloc, C., Verissimo, A. S., Franc, N. & Purper-Ouakil, D. (2022). Parent training for the treatment of irritability in children and adolescents: A multisite randomized controlled, 3-parallel-group, evaluator-blinded, superiority trial. *BMC Psychology, 10*(1), 273.	Ongoing study
Fossum, S. Handegard, B. H., Adolfsen, F. Vis, S. A., & Wynn, R. (2016). A meta-analysis of long-term outpatient treatment effects for children and adolescents with conduct problems. *Journal of Child and Family Studies, 25*(1), 15-29.	Review
Fossum, S., Handegard, B. H., Martinussen, M., & Morch, W. T (2008). Psychosocial interventions for disruptive and aggressive behaviour in children and adolescents: A meta-analysis. *European Child & Adolescent Psychiatry, 17*(7), 438-451.	Review
Fragkaki, I. (2020). Emotion recognition training combined with oxytocin in residential youth. *World Health Organization International Clinical Trials Registry Platform.* http://www.who.int/trialsearch/Trial2.aspx?TrialID=EUCTR2019-001910-40-NL	Wrong outcomes
Fraser, M. W., Day, S. H., Galinsky, M. J., Hodges, V. G., & Smokowski, P. R. (2004). Conduct problems and peer rejection in childhood: A randomized trial of the Making Choices and Strong Families programs. *Research on Social Work Practice, 14*(5), 313-324.	Children (0-11)
Fraser, M. W., Day, S. H., Galinsky, M. J., Hodges, V. G., & Smokowski, P. R. (2004). Conduct problems and peer rejection in childhood: A randomized trial of the Making Choices and Strong Families programs. *Research on Social Work Practice, 14*(5), 313-324.	Children (0-11)
Frederickson, N. Jones, A. P., Warren, L., Deakes, T., & Allen, G. (2013). Can developmental cognitive neuroscience inform intervention for social, emotional and behavioural difficulties (SEBD)? *Emotional & Behavioural Difficulties, 18*(2), 135-154.	Not an RCT or quasi study
Frey, K. S., Nolen, S. B., Van Schoiack Edstrom, L., & Hirschstein, M. K. (2005). Effects of a school-based social-emotional competence program: Linking children's goals, attributions, and behavior. *Journal of Applied Developmental Psychology, 26*(2), 171-200.	Children (0-11)
Frick, P. J. & Kemp, E. C. (2021). Conduct disorders and empathy development. *Annual Review og Clinical Psychology, 17*, 391-416.	Review
Frick, P. J., Ray, J. V., Thornton, L. C., & Kahn, R. E. (2014). Can callous-unemotional traits enhance the understanding, diagnosis, and treatment of serious	Review

conduct problems in children and adolescents? A comprehensive review. *Psychological Bulletin, 140*(1), 1-57.	
Froehlich, J. Doepfner, M. & Lehmkuhl, G. (2002). Effects of combined cognitive behavioural treatment with parent management training in ADHD. *Behavioural and Cognitive Psychotherapy, 30*(1), 111-115.	Children (0-11)
Frolich, J., Dopfner, M., Berner, W., & Lehmkuhl, G. (2002). Combined cognitive behavioral treatment with parent management training in ADHD. *Praxis der Kinderpsychologie und Kinderpsychiatrie, 51*(6), 476-493.	Duplicate
Galbraith, K., Tarbo, J. & Huey, S. J. (2022). Assessing the feasibility of peer coach training for disruptive middle school youth: A mixed methods pilot study. *Journal of Child and Family Studies.* DOI: 10.1007/s10826-022-02504-w	Not an RCT or quasi study
Garland, A F., Accurso, E. C., Haine-Schlagel, R., Brookman-Frazee, L., Roesch, S., & Zhang, J. J. (2014). Searching for elements of evidence-based practices in children's usual care and examining their impact. *Journal of Clinical Child and Adolescent Psychology, 43*(2), 201-215.	Review
Garland, A.F., Hawley, K. M., Brookman-Frazee, L., & Hurlburt, M. S. (2008). Identifying common elements of evidence-based psychosocial treatments for children's disruptive behavior problems. *Journal of the American Academy of Child & Adolescent Psychiatry, 47*(5), 505-514.	Review
Gavita, O. A., David, D., Bujoreanu, S., Tiba, A., & Ionutiu, D. R. (2012). The efficacy of a short cognitive-behavioral parent program in the treatment of externalizing behavior disorders in Romanian foster care children: Building parental emotion-regulation through unconditional self- and child-acceptance strategies. *Children and Youth Services Review, 34*(7), 1290-1297.	Children (0-11)
Gavita, O. A., David, D., Bujoreanu, S., Tiba, A., & Ionutiu, D. R. (2012). The efficacy of a short cognitive-behavioral parent program in the treatment of externalizing behavior disorders in Romanian foster care children: Building parental emotion-regulation through unconditional self- and child-acceptance strategies. *Children and Youth Services Review, 34*(7), 1290-1297.	Duplicate
Geissler, J., Jans, T., Banaschewski, T., Becker, K., Renner, T., Brandeis, D., Dopfner, M., Dose, C., Hautmann, C., Holtmann, M., Jenkner, C., Millenet, S., & Romanos, M. (2018). Individualized short-term therapy for adolescents impaired by ADHD despite previous routine care treatment (ESCAdol) - a randomized controlled trial within the consortium 'ESCAlife'. *Trials, 19*¸ https://doi.org/10.1186/s13063-018-2635-2	Wrong population
Gewirtz, A. H., Lee, S. S., August, G. J., & He, Y. (2019). Does giving parents their choice of interventions for child behavior problems improve child outcomes? *Prevention Science, 20*(1), 78-88.	Children (0-11)
Ghaderi, A., Kadesjo, C., Bjornsdotter, A., & Enebrink, P. (2018). Randomized effectiveness Trial of the Family Check-Up versus Internet-delivered Parent Training (iComet) for Families of Children with Conduct Problems. *Scientific Reports, 8*(1), doi:10.1038/s41598-018-29550-z	Wrong outcomes
Gillen, C. T. A. (2018). *Psychopathic traits, substance use, and motivation to change: The effectiveness of motivational interviewing with at-risk adolescents* [Unpublished Doctoral Dissertation]. The University of Southern Mississippi.	CU traits and/or antisocial behavior not measured at post

Glover-Orr, L. (1999). *The efficacy of an anger management group for children with disruptive behavior disorders. (cognitive behavioral therapy)* [Unpublished Doctoral Dissertation]. University of Iowa.	Not an RCT or quasi study
Goldbeck, L., Muche, R., Sachser, C., Tutus, D., & Rosner, R. (2016). Effectiveness of trauma-focused cognitive behavioral therapy for children and adolescents: A randomized controlled trial in eight German mental health clinics. *Psychotherapy and Psychosomatics, 85*(3), 159-170.	Wrong outcomes
Goldstein, N. E. S., Dovidio, A., Kalbeitzer, R., Weil, J., & Strachan, M. (2007). Anger management for female juvenile offenders: Results of a pilot study. *Journal of Forensic Psychology Practice, 7*(2), 1-28.	Wrong outcomes
Golubchik, P., Mozes, T., Maayan, R., & Weizman, A. (2009). Neurosteroid blood levels in delinquent adolescent boys with conduct disorder. *European Neuropsychopharmacology, 19*(1), 49-52.	No treatment tested/given
Gomez, M. J., Luciano, C., Paez-Blarrina, M., Ruiz, F. J., Valdivia-Salas, S., & Gil-Luciano, B. (2014). Brief ACT protocol in at-risk adolescents with conduct disorder and impulsivity. *International Journal of Psychology & Psychological Therapy, 14*(3), 307-332.	Not an RCT or quasi study
Gonzalez, J. E., Nelson, J., Gutkin, T. B., Saunders, A., Galloway, A., & Shwery, C. S. (2004). Rational Emotive Therapy with children and adolescents: A meta-analysis. *Journal of Emotional and Behavioral Disorders, 12*(4), 222-235.	Review
Gordon, J. A. (1997). *An evaluation of Paint Creek Youth Center* [Unpublished doctoral dissertation]. University of Cincinnati.	Not an RCT or quasi study
Gortz-Dorten, A. & Dopfner, M. (2017). Systematic and empirical results on conduct disorders in childhood and adolescence. *Forensische Psychiatrie, Psychologie, Kriminologie, 11*(2), 103-110.	Review
Goteborg University. (2021). Two-year Follow-up of a Mindfulness-based Intervention in Children. *U.S National Library of Medicine Clinical Trials.* https://clinicaltrials.gov/ct2/show/NCT04806542	Wrong outcomes
Granski, M., Javdani, S., Anderson, V. R., & Caires, R. (2020). A meta-analysis of program characteristics for youth with disruptive behavior problems: The moderating role of program format and youth gender. *American Journal of Community Psychology*, *65*(1-2), 201-222.	Review
Grasmann, D. & Stadler, C. (2011). VIA-An intensive therapeutic treatment program for conduct disorders. *Zeitschrift fur Kinder- und Jugendpsychiatrie und Psychotherapie, 39*(1), 23-31.	Children (0-11)
Green, J., Biehal, N., Roberts, C., Dixon, J., Kay, C., Parry, E., Rothwell, J., Roby, A., Kapadia, D., Scott, S., & Sinclair, I. (2014). Multidimensional treatment foster care for adolescents in English care: Randomised trial and observational cohort evaluation. *The British Journal of Psychiatry, 204*(3), 204-214.	Wrong outcomes
Green, J., Biehal, N., Roberts, C., Dixon, J., Kay, C., Parry, E., Rothwell, J., Roby, A., Kapadia, D., Scott, S., & Sinclair, I. (2014). Multidimensional treatment foster care for adolescents in English care: Randomised trial and observational cohort evaluation. *The British Journal of Psychiatry, 204*(3), 204-214.	Duplicate
Greene, R., & Winkler, J. (2019). Collaborative & Proactive Solutions (CPS): A review of research findings in families, schools, and treatment facilities. *Clinical child and family psychology review, 22*(4), 549-561.	Review

Grella, C. E., Hser, Y. I., Joshi, V., & Rounds-Bryant, J. (2001). Drug treatment outcomes for adolescents with comorbid mental and substance use disorders. *Journal of Nervous and Mental Disease, 189*(6), 384-392.	Not an RCT or quasi study
Griffith, M. L. (2010). *Experiential therapy and empathy in oppositional defiant disorder adolescents* [Unpublished doctoral dissertation]. The Alder School of Professional Psychology.	Wrong outcomes
Grist, R., Porter, J., & Stallard, P. (2017). Mental health mobile apps for preadolescents and adolescents: A systematic review. *Journal of Medical Internet Research, 19*(5), 153-166.	Review
Groenman, A. P., Hornstra, R., Hoekstra, P. J., Steenhuis, L., Aghebati, A., Boyer, B. E., Buitelaar, J. K., Chronis-Tuscano, A., Daley, D., Dehkordian, P., Dvorsky, M., Franke, N., DuPaul, G. J., Gershy, N., Harvey, E., Hennig, T., Herbert, S., Langberg, J., Mautone, J. A., Mikami, A. Y., Pfiffner, L. J., Power, T. J., Reijneveld, S. A., Schramm, S. A., Schweitzer, J. B., Sibley, M. H., Sonuga-Barke, E., Thompson, C., Thompson, M., Webster-Stratton, C., Xie, Y., Luman, M., van der Oord, S. & van den Hoofdakker, B. J. (2022). An individual participant data meta-analysis: Behavioral treatments for children and adolescents with attention-deficit/hyperactivity disorder. *Journal of the American Acadamy of Child & Adolescent Psychiatry, 61*(2), 144-158.	Review
Grondhuis, S. N., Farmer, C. A., Arnold, L. E., Gadow, K. D., Findling, R. L., Molina, B. S. G., Kolko, D. J., Buchan-Page, K. A., Rice, R. R., Butter, E. M., & Aman, M. G. (2020). Standardized observation analogue procedure in the treatment of severe childhood aggression study. *Journal of Child and Adolescent Psychopharmacology, 30*(1), 48-54.	Children (0-11)
Groves, S., Backer, H. S., van den Bosch, W., & Miller, A. (2012). Review: Dialectical behaviour therapy with adolescents. *Child and Adolescent Mental Health, 17*(2), 65-75.	Review
Groves, S., Backer, H. S., van den Bosch, W., & Miller, A. (2012). Review: Dialectical behaviour therapy with adolescents. *Child and Adolescent Mental Health, 17*(2), 65-75.	Duplicate
Gustis, C. M. (2007). *Behavioral intervention and parent training within the CASSP system: The effectiveness of using direct commands to manage ADHD/ODD in the home environment* [Unpublished doctoral dissertation]. Capella University.	Children (0-11)
Haack, L. M., Araujo, E. A., Meza, J., Friedman, L. M., Spiess, M., Alcaraz Beltran, D. K., Delucchi, K., Mojardin Herladez, A., & Pfiffner, L. (2020). Can school mental health providers deliver psychosocial treatment improving youth attention and behavior in Mexico? A pilot randomized controlled trial of CLS-FUERTE. *Journal of Attention Disorders,* doi:10.1177/1087054720959698	Children (0-11)
Hair, H. J. (2005). Outcomes for children and adolescents after residential treatment: A review of research from 1993 to 2003. *Journal of Child and Family Studies, 14*(4), 551-575.	Review
Haller, S. P., Stoddard, J., Botz-Zapp, C., Clayton, M., MacGillivray, C., Perhamus, G., Stiles, K., Kircanski, K., Penton-Voak, I. S., Bar-Haim, Y., Munafo, M., Towbin, K. E., & Brotman, M. A. (2021). A randomized controlled trial of computerized interpretation bias training for disruptive mood dysregulation disorder: A fast-fail study. *Journal of the American Academy of Child & Adolescent Psychiatry, 61*(1), 37-45.	Children (0-11)

Haller, S. P., Stoddard, J., MacGillivray, C., Stiles, K., Perhamus, G., Penton-Voak, I. S., Bar-Haim, Y., Munafo, M. R., & Brotman, M. A. (2018). A double-blind, randomized, placebo-controlled trial of a computer-based Interpretation Bias Training for youth with severe irritability: A study protocol. *Trials*, *19*(1), https://doi.org/10.1186/s13063-018-2960-5	Wrong population
Hammer, J. A. (2011). *The effect of cognitive-behavioral therapy on juvenile criminal thinking* [Unpublished doctoral dissertation]. Capella University	Wrong outcomes
Hansson, K., & Olsson, M. (2012). Effects of multidimensional treatment foster care (MTFC): Results from a RCT study in Sweden. *Children and Youth Services Review, 34*(9), 1929-1936.	Wrong outcomes
Hartnett, D., Carr, A., Hamilton, E., & O'Reilly, G. (2017). The effectiveness of functional family therapy for adolescent behavioral and substance misuse problems: A meta-analysis. *Family Process, 56*(3), 607-619.	Review
Hartnett, D., Carr, A., & Sexton, T. (2016). The effectiveness of functional family therapy in reducing adolescent mental health risk and family adjustment difficulties in an Irish context. *Family Process, 55*(2), 287-304.	Wrong outcomes
Hasani, S. M., Askary, P., Heidari, A., & Zadeh, P. E. (2020). The comparative effectiveness of parental behavior management training and schema therapy on aggression and oppositional defiant in adolescents. *Journal of Nursing & Midwifery Sciences, 7*(3), 146-152.	Wrong outcomes
Hautmann, C., Stein, P., Hanisch, C., Eichelberger, I., Pluck, J., Walter, D., & Dopfner, M. (2009). Does parent management training for children with externalizing problem behavior in routine care result in clinically significant changes? *Psychotherapy Research, 19*(2), 224-33.	Children (0-11)
Hawes, D. J., Price, M. J., & Dadds, M. R. (2014). Callous-unemotional traits and the treatment of conduct problems in childhood and adolescence: A comprehensive review. *Clinical Child and Family Psychology Review, 17*(3), 248-267.	Review
Hawkins, J., Catalano, R. F., Arthur, M. W., Egan, E., Brown, E. C., Abbott, R. D., & Murray, D. M. (2008). Testing communities that care: The rationale, design and behavioral baseline equivalence of the community youth development study. *Prevention Science, 9*(3), 178-190.	Wrong intervention
Hawkins, R. O., Collins, T. A., Haas Ramirez, L., Murphy, J., & Ritter, C. (2020). Examining the generalization of a combined independent and interdependent group contingency for students with emotional and behavioral disorders. *Behavioral Disorders, 45*(4), 238-251.	Wrong outcomes
Haynes, J. (2003). *The impact of group therapy, cooperative learning, and skills training on foster care children in a community-based program* [Unpublished doctoral dissertation].	Not retrievable
Hebert, D. A. (2002). *Evaluating cognitive-behavioral group treatment for disruptive adolescents in a special school* [Unpublished doctoral dissertation]. Antioch Univeristy	Wrong outcomes
Hekmat, M. R., & Islamic Azad University (2021). Comparing the effectiveness of positivist psychology education and cognitive-behavioral therapy through emotion regulation on cognitive flexibility, cognitive strategies of emotion regulation, impulsivity, and social well-being of male adolescents with conduct disorder. *World*	Wrong outcomes

Health Organization International Clinical Trials Registry Platform. https://trialsearch.who.int/Trial2.aspx?TrialID=IRCT20201221049781N1	
Helander, M., Lochman, J., Högström, J., Ljótsson, B., Hellner, C., & Enebrink, P. (2018). The effect of adding Coping Power Program-Sweden to Parent Management Training-effects and moderators in a randomized controlled trial. *Behaviour Research and Therapy, 103,* 43–52. https://doi.org/10.1016/j.brat.2018.02.001	Children (0-11)
Helender, M., Enebrink, P., Hellner, C. & Ahlen, J. (2022). Parent management training combined with group-cbt compared to parent management training only for oppositional defiant disorder symptoms: 2-year follow-up of a randomized controlled trial. *Child Psychiatry and Human Development*, DOI: 10.1007/s10578-021-01306-3	Children (0-11)
Hendriks, V., van der Schee, E., & Blanken, P. (2012). Matching adolescents with a cannabis use disorder to multidimensional family therapy or cognitive behavioral therapy: Treatment effect moderators in a randomized controlled trial. *Drug and Alcohol Dependence, 125*(1-2), 119-126-	Wrong outcomes
Henggeler, S. W., Clingempeel, W. G., Brondino, M. J., & Pickrel, S. G. (2002). Four-year follow-up of multisystemic therapy with substance-abusing and substance-dependent juvenile offenders. *Journal of the American Academy of Child and Adolescent Psychiatry, 41*(7), 868-874.	Wrong outcomes
Henggeler, S. W., Melton, G. B., & Smith, L. A. (1992). Family preservation using multisystemic therapy: an effective alternative to incarcerating serious juvenile offenders. *Journal of Consulting and Clinical Psychology*, *60*(6), 953-961.	Wrong outcomes
Henggeler, S. W., Letourneau, E. J., Chapman, J. E., Borduin, C. M., Schewe, P. A., & McCart, M. R. (2009). Mediators of change for multisystemic therapy with juvenile sexual offenders. *Journal of Consulting and Clinical Psychology*, *77*(3), 451-462.	Wrong outcomes
Henggeler, S. W., Rowland, M. D., Randall, J., Ward, D. M., Pickrel, S. G., Cunningham, P. B., Miller, S. L., Edwards, J., Zealberg, J. J., Hand, L. D., & Santos, A. B. (1999). Home-based multisystemic therapy as an alternative to the hospitalization of youths in psychiatric crisis: Clinical outcomes. *Journal of the American Academy of Child & Adolescent Psychiatry*, *38*(11), 1331-1339.	Wrong outcomes
Heywood, C. & Fergusson, D. (2016). A pilot study of functional family therapy in New Zealand. *New Zealand Journal of Psychology, 45*(3), 12-22.	Not an RCT or quasi study
Hiemstra, W., & Orobio De Castro, B. (2015). Reducing aggressive behaviour by targeting social information processing in referred boys: An implicit intervention. *European Child and Adolescent Psychiatry,* (Suppl.), S162-S163. 5) doi:10.1007/s00787-015-0714-4	Symposia/abstract for a conference
Hilton, R. C., Rengasamy, M., Mansoor, B., He, J., Mayes, T., Emslie, G. J., Porta, G., Clarke, G. N., Wagner, K. D., Birmaher, B., Keller, M. B., Ryan, N., Shamseddeen, W., Asarnow, J. R., & Brent, D. A. (2013). Impact of treatments for depression on comorbid anxiety, attentional, and behavioral symptoms in adolescents with selective serotonin reuptake inhibitor-resistant depression. J*ournal of the American Academy of Child & Adolescent Psychiatry*, *52*(5), 482-492.	Wrong outcomes
Hoag, M. J. & Burlingame, G. M. (1997). Evaluating the effectiveness of child and adolescent group treatment: A meta-analytic review. *Journal of Clinical Child Psychology*, *26*(3), 234-246.	Review

Hoagwood, K. E., Acri, M., Morrissey, M., & Peth-Pierce, R. (2017). Animal-assisted therapies for youth with or at risk for mental health problems: A systematic review. *Applied Developmental Science, 21*(1), 1-13.	Review
Hoffman, S., Cummings, A. L., & Leschied, A. W. (2004). Treating aggression in high-risk adolescent girls: A preliminary evaluation. *Canadian Journal of Counselling, 38*(2), 59-73.	Not an RCT or quasi study
Hogue, A., Henderson, C. E., Dauber, S., Barajas, P. C., Fried, A., & Liddle, H. A. (2008). Treatment adherence, competence, and outcome in individual and family therapy for adolescent behavior problems. *Journal of Consulting & Clinical Psychology*, *76*(4), 544-555.	Wrong outcomes
Hogue, A., Dauber, S., Henderson, C. E., Bobek, M., Johnson, C., Lichvar, E., & Morgenstern, J. (2015). Randomized trial of family therapy versus nonfamily treatment for adolescent behavior problems in usual care. *Journal of Clinical Child and Adolescent Psychology, 44*(6), 954-969.	Wrong outcomes
Hoogendijk, C., Tick, N. T., Holland, J. G., Hofman, W. H. A., Severiens, S. E., Vuijk, P., & van Veen, A. F. D. (2020). Effects of Key2Teach on students' externalising and social-emotional problem behaviours, mediated by the teacher-student relationship. *Emotional & Behavioural Difficulties, 25*(3/4), 304-320.	Children (0-11)
Hoogsteder, L. M., Oomen, P., Sweers, N., & Hendriks, J. (2022). A study on the effects of responsive outpatient aggression regulation therapy for juveniles. *Journal of Forensic Mental Health, 12,* DOI: 10.1080/14999013.2022.2129524	Wrong outcomes
Hoogsteder, L. M., Stams, G., Schippers, E. E., & Bonnes, D. (2018). Responsive aggression regulation therapy (Re-ART): An evaluation study in a Dutch juvenile justice institution in terms of recidivism. International *Journal of Offender Therapy and Comparative Criminology*, *62*(14), 4403-4424.	Wrong outcomes
Hoogsteder, L. M., van Horn, J. E., Stams, G. J. J. M., Wissink, I. B., & Hendriks, J. (2016). The relationship between the level of program integrity and pre- and post-Test changes of responsive-aggression regulation therapy (Re-ART) outpatient: A pilot study. *International Journal of Offender Therapy and Comparative Criminology, 60*(4), 435.	Adults (18+)
Horigian, V. E., Feaster, D. J., Robbins, M. S., Brincks, A. M., Ucha, J., Rohrbaugh, M. J., Shoham, V., Bachrach, K., Miller, M., Burlew, A., Hodgkins, C. C., Carrion, I. S., Silverstein, Me., Werstlein, R. & Szapocznik, J. (2015). A cross-sectional assessment of the long term effects of Brief Strategic Family Therapy for adolescent substance use. *The American Journal on Addictions, 24*(7), 637-645.	Wrong outcomes
Hsu, K. S., Eads, R., Lee, M. Y., & Wen, Z. (2021). Solution-focused brief therapy for behavior problems in children and adolescents: A meta-analysis of treatment effectiveness and family involvement. *Children & Youth Services Review, 120,* https://doi.org/10.1016/j.childyouth.2020.105620	Review
Hubble, K., Bowen, K. L., Moore, S. C., & van Goozen, S. H. M. (2015). Improving negative emotion recognition in young offenders reduces subsequent crime. *PLoS ONE 10*(6), doi:10.1371/journal.pone.0132035	CU and/or antisocial behavior not measured at post
Hukkelberg, S., Ogden, T. & Thøgersen, D. M. (2022). Youth level of service/case management inventory assessments as predictors of behavioral change in Multisystemic Therapy and Functional Family Therapy in Norway. *Research on Social Work Practice, 32*(7), 784-792.	Wrong outcomes

Humayun, S., Herlitz, L., Chesnokov, M., Doolan, M., Landau, S., & Scott, S. (2017). Randomized controlled trial of Functional Family Therapy for offending and antisocial behavior in UK youth. *Journal of Child Psychology and Psychiatry, 58*(9), 1023-1032.	Wrong outcomes
Humayun, S. (2010). Study of adolescents' family experiences. *World Health Organization International Clinical Trials Registry Platform.* http://www.who.int/trialsearch/Trial2.aspx?TrialID=ISRCTN27650478	Wrong outcomes
Hutchings, J., Appleton, P., Smith, M., Lane, E., & Nash, S. (2002). Evaluation of two treatments for children with severe behaviour problems: Child behaviour and maternal mental health outcomes. *Behavioural and Cognitive Psychotherapy, 30*(3), 279-295.	Children (0-11)
Icahn School of Medicine at Mount Sinai (2006). Multi-family group therapy for reducing behavioral difficulties in youth. *U.S National Library of Medicine Clinical Trials.* https://clinicaltrials.gov/ct2/show/NCT00404911	Children (0-11)
Irvine, A., Biglan, A., Smolkowski, K., Metzler, C. W., & Ary, D. V. (1999). The effectiveness of a parenting skills program for parents of middle school students in small communities. *Journal of Consulting and Clinical Psychology, 67*(6), 811-825.	Wrong outcomes
Islamic Azad University (2021). Comparing the effectiveness of positivist psychology education and Cognitive-Behavioral Therapy through emotion regulation on cognitive flexibility, cognitive strategies of emotion regulation, impulsivity, and social well-being of male adolescents with conduct disorder. *World Health Organization International Clinical Trials Registry Platform.* https://trialsearch.who.int/Trial2.aspx?TrialID=IRCT20201221049781N1	Protocol
Jalling, C. *Prevention and prediction of adolescent problem behavior* [Unpublished Doctoral Dissertation]. Department of Clinical Neuroscience Karolinska Institute. Stockholm, Sweden	Wrong outcomes
Jalling, C., Bodin, M., Romelsjo, A., Kallmen, H., Durbeej, N., & Tengstrom, A. (2016). Parent programs for reducing adolescent's antisocial behavior and substance use: A randomized controlled trial. *Journal of Child and Family Studies, 25*(3), 811-82.	Wrong outcomes
Jambroes, T., Jansen, L. M., Oostermeijer, S., Ven, Peter M., Doreleijers, T. A., Vermeiren, R. R., & Popma, A. (2019). CU-traits and HPA-axis reactivity conjointly relate to treatment effect in adolescents with severe antisocial behavior. *Journal of Criminal Justice, 65*(8), https://doi.org/10.1016/j.jcrimjus.2017.12.011	Not an RCT or quasi study
Janus, M. D. (1993). *Affective empathy training in the treatment of conduct disordered adolescents* [Unpublished doctoral dissertation]. University of Connecticut.	Wrong outcomes
Jarden, H. W. (1994). *A comparison of problem-solving interventions on the functioning of youth with disruptive behavior disorders* [Unpublished doctoral dissertation]. Lehigh University.	Wrong outcomes
Jarden, H. W. (1995). *A comparison of problem-solving interventions on the functioning of youth with disruptive behavior disorders* [Unpublished doctoral dissertation]. Lehigh University.	Duplicate
Jayalekshmi, N. B. & Raja, B. W. D. (2014). Institutional influence on behavioural disorders in early adolescents. *Journal on Educational Psychology, 8*(1), 42-46.	Not an RCT or quasi study

Jent, J. F. & Niec, L. N. (2009). Cognitive behavioral principles within group mentoring: A randomized pilot study. *Child & Family Behavior Therapy, 31*(3), 203-219.	Children (0-11)
Jeppesen, P., & Mental Health Services in the Capital Region, Denmark (2021). Randomized feasibility trial of Mind My Mind. *U.S National Library of Medicine Clinical Trials.* https://clinicaltrials.gov/ct2/show/NCT03448809	Wrong outcomes
Jeppesen, P., Wolf, R. T., Nielsen, S. M., Christensen, R., Plessen, K. J., Bilenberg, N., Thomsen, P. H., Thastum, M., Neumer, S. P., Puggaard, L. B., Agner Pedersen, M. M., Pagsberg, A. K., Silverman, W. K., & Correll, C. U. (2021). Effectiveness of transdiagnostic cognitive-behavioral psychotherapy compared with management as usual for youth with common mental health problems: A randomized clinical trial. *JAMA Psychiatry, 78*(3), 250-260.	Children (0-11)
Jerrott, S., Clark, S. E., & Fearon, I. (2010) Day treatment for disruptive behaviour disorders: Can a short-term program be effective? *Journal of the Canadian Academy of Child and Adolescent Psychiatry, 19*(2), 88-93.	Children (0-11)
Johns Hopkins Bloomberg School of Public Health (2017). An evaluation of an integrated approach to prevention and early intervention in the elementary school years. *U.S National Library of Medicine Clinical Trials.* https://clinicaltrials.gov/ct2/show/NCT03132805	Wrong population
Johnson, M., Ostlund, S., Fransson, G., Landgren, M., Nasic, S., Kadesjo, B., Gillberg, C., & Fernell, E. (2012). Attention-deficit/hyperactivity disorder with oppositional defiant disorder in Swedish children - An open study of collaborative problem solving. *Acta Paediatrica, 101*(6), 624-630.	Children (0-11)
Johnson, M. E. & Waller, R. J. (2006). A review of effective interventions for youth with aggressive behaviors who meet diagnostic criteria for conduct disorder or oppositional defiant disorder. *Journal of Family Psychotherapy, 17*(2), 67-80.	Review
Jolley, S., Kuipers, E., Stewart, C., Browning, S., Bracegirdle, K., Basit, N., Gin, K., Hirsch, C., Corrigall, R., Banerjea, P., Turley, G., Stahl, D., & Laurens, K. R. (2018). The coping with unusual experiences for children study (CUES): a pilot randomized controlled evaluation of the acceptability and potential clinical utility of a cognitive behavioural intervention package for young people aged 8–14 years with unusual ex. *British Journal of Clinical Psychology, 57*(3), 328-350.	Children (0-11)
Jones, D. R. (2015). *Examining the impact of a positive behavior support program and direct instruction of social and emotional learning skills on the externalizing behaviors of disruptive youth* [Unpublished doctoral dissertation]. Drexel University.	Wrong Outcomes
Joseph, M., & Dubey, S. (2012). Effect of family therapy on physical and psychological distress and personality of adolescents. *Indian Journal of Community Psychology, 8*(2), 232-247	Supplement with no publication
Kaminer, Y., & Burleson, J. A. (1999). Psychotherapies for adolescent substance abusers: 15-month follow-up of a pilot study. *American Journal on Addictions, 8*(2), 114-119.	Wrong outcomes
Kamps, D., Kravits, T., Stolze, J., & Swaggart, B. (1999). Prevention strategies for at-risk students and students with EBD in urban elementary schools. *Journal of Emotional and Behavioral Disorders, 7*(3), 178-188.	Wrong intervention

Kaur, M., Floyd, A. & Balta, A. M. (2022). Oppositional defiant disorder: Evidence-based review of behavioral treatment programs. *Annals of Clinical Psychiatry, 34*(1), 44-58.	Review
Kaya, F., & Buzlu, S. (2016). Effects of Aggression Replacement Training on problem solving, anger and aggressive behaviour among adolescents with criminal attempts in Turkey: A quasi-experimental study. *Archives of Psychiatric Nursing, 30*(6), 729-735.	Wrong outcomes
Kazdin, A. E. (2018). Implementation and evaluation of treatments for children and adolescents with conduct problems: Findings, challenges, and future directions. *Psychotherapy Research, 28*(1), 3-17.	Not an RCT or quasi study
Kazdin, A. E., & Crowley, M. J. (1997). Moderators of treatment outcome in cognitively based treatment of antisocial children. *Cognitive Therapy and Research, 21*(2), 185-207.	Children (0-11)
Kazdin, A. E., Glick, A., Pope, J., Kaptchuk, T. J., Lecza, B., Carrubba, E., McWhinney, E., & Hamilton, N. (2018). Parent management training for conduct problems in children: Enhancing treatment to improve therapeutic change. *International Journal of Clinical and Health Psychology, 18*(2), 91-101.	Children (0-11)
Kazdin, A. E., Siegel, T. C., & Bass, D. (1992). Cognitive problem-solving skills training and parent management training in the treatment of antisocial behavior in children. *Journal of Consulting and Clinical Psychology, 60*(5), 733-747.	Children (0-11)
Kazdin, A. E. & Wassell, G. (2000). Therapeutic changes in children, parents, and families resulting from treatment of children with conduct problems. *Journal of the American Academy of Child & Adolescent Psychiatry, 39*(4), 414-420.	Children (0-11)
Kazdin, A. E., & Whitley, M. K. (2003). Treatment of parental stress to enhance therapeutic change among children referred for aggressive and antisocial behavior. *Journal of Consulting and Clinical Psychology, 71*(3), 504-515.	Children (0-11)
Kearney, B. W. (2001). *Empathy development in adolescents with conduct disorders using relationship enhancement therapy* [Unpublished doctoral dissertation]. Cleveland State University.	Wrong outcomes
Kellner, M. H., Bry, B. H., & Salvador, D. D. (2008). Anger management effects on middle school students with emotional/behavioral disorders: Anger log use, aggressive and prosocial behavior. *Child & Family Behavior Therapy, 30*(3), 215-230.	Wrong outcomes
Kelsberg, G., & St. Anna, L. (2006). What are effective treatments for oppositional defiant behaviors in adolescents? *Journal of Family Practice, 55*(10), 911-913.	Review
Kelsberg, G., & St. Anna, L. (2006). What are effective treatments for oppositional defiant behaviors in adolescents? *The Journal of Family Practice, 55*(10), 911-913.	Duplicate
Kemmis-Riggs, J., Dickes, A., & McAloon, J. (2018). Program components of psychosocial interventions in foster and kinship care: A systematic review. *Clinical Child and Family Psychology Review, 21*(1), 13-40.	Review
Kendall, P. C., Reber, M., McLeer, S., Epps, J., & Ronan, K. R. (1990) .Cognitive-behavioral treatment of conduct-disordered children. *Cognitive Therapy and Research, 14*(3), 279-297.	Children (0-11)
Kendrick, C. P. (1995). *A quasi-experimental study of the effect of feedback on the social behavior of school children with attention deficit hyperactivity disorder* [Unpublished doctoral dissertation]. University of Arkansas.	Wrong outcomes

Kersten, L., Pratzlich, M., Mannstadt, S., Ackermann, K., Kohls, G., Oldenhof, H., Saure, D., Krieger, K., Herpertz-Dahlmann, B., Popma, A., Freitag, C. M., Trestman, R. L., & Stadler, C. (2016). START NOW - a comprehensive skills training programme for female adolescents with oppositional defiant and conduct disorders: study protocol for a cluster-randomised controlled trial. *Trials, 17*(1), 568.	Wrong outcomes
Kersten, L., Pratzlich, M., Mannstadt, S., Ackermann, K., Kohls, G., Oldenhof, H., Saure, D., Krieger, K., Herpertz-Dahlmann, B., Popma, A., Freitag, C. M., Trestman, R. L., & Stadler, C. (2017). Erratum to: START NOW - a comprehensive skills training programme for female adolescents with oppositional defiant and conduct disorders: study protocol for a clusterrandomised controlled trial. *Trials, 18*(1), no pagination.	Supplement (no publication)
Kersten, L., Pratzlich, M., Mannstadt, S., Ackermann, K., Kohls, G., Oldenhof, H., Saure, D., Krieger, K., Herpertz-Dahlmann, B., Popma, A., Freitag, C. M., Trestman, R. L., & Stadler, C. (2016). Erratum to: START NOW - a comprehensive skills training programme for female adolescents with oppositional defiant and conduct disorders: study protocol for a clusterrandomised controlled trial. *Trials, 18*(1), no pagination.	Duplicate
Kjobli, J., Hukkelberg, S., & Ogden, T. (2013). A randomized trial of group parent training: Reducing child conduct problems in real-world settings. *Behaviour Research and Therapy, 51*(3), 113-121.	Children (0-11)
Klasen, H., & Crombag, A. C. (2013). What works where? A systematic review of child and adolescent mental health interventions for low and middle income countries. *Social Psychiatry and Psychiatric Epidemiology, 48*(4), 595-611.	Review
Kliem, S., Aurin, S. S., & Kroger, C. (2014). Efficacy of a positive parenting program for parents of teenagers: A randomized controlled trial. *Kindheit Und Entwicklung, 23*(3), 184-193.	Wrong outcomes
Kloft, L., Hawes, D., Moul, C., Sultan, S., & Dadds, M. (2017). Family drawings before and after treatment for child conduct problems: Fluidity of family dysfunction. *Journal of Child & Family Studies, 26*(12), 3476-3489.	Children (0-11)
Klug, W. (2000). *Nonassertive mothers, aggressive teens: Toughlove as a community intervention* (Report No. ED 488 395). Berkshire Community College.	Not an RCT or quasi study
Knopes, D. R. (2004). *Motivating change in high-risk adolescents: An intervention focus on the deviant friendship process* [Unpublished doctoral dissertation]. University of Oregon.	Wrong outcomes
Kolko, D. J., Campo, J. V., Kelleher, K., & Cheng, Y. (2010). Improving access to care and clinical outcome for pediatric behavioral problems: A randomized trial of a nurse-administered intervention in primary care. *Journal of Developmental and Behavioral Pediatrics, 31*(5), 393-404.	Children (0-11)
Kolko, D. J., Cheng, Y., Campo, J. V., & Kelleher, K. (2011). Moderators and predictors of clinical outcome in a randomized trial for behavior problems in pediatric primary care. *Journal of Pediatric Psychology, 36*(7), 753-765.	Children (0-11)
Kolko, D. J., Baumann, B. L., Bukstein, O. G., & Brown, E. J. (2007). Internalizing symptoms and affective reactivity in relation to the severity of aggression in clinically referred, behavior-disordered children. *Journal of Child and Family Studies, 16*(6), 745-759.	Children (0-11)

Kolko, D. J., Bukstein, O. G., & Barron, J. (1999). Methylphenidate and behavior modification in children with ADHD and comorbid ODD or CD: Main and incremental effects across settings. *Journal of the American Academy of Child & Adolescent Psychiatry, 38*(5), 578-586.	Children (0-11)
Krischer, M., Smolka, B., Voigt, B., Lehmkuhl, G., Flechtner, H. H., Franke, S., Hellmich, M., & Trautmann-Voigt, S. (2020). Effects of long-term psychodynamic psychotherapy on life quality in mentally disturbed children. *Psychotherapy Research, 30*(8), 1039-1047.	Wrong outcomes
Krishnamoorthy, G., Hessing, P., Middeldorp, C., & Branjerdporn, M. (2020). Effects of the 'Circle of Security' group parenting program (COS-P) with foster carers: An observational study. *Children & Youth Services Review, 115*, https://doi.org/10.1016/j.childyouth.2020.105082	Not an RCT or quasi study
Kumar, G. (2009). Impact of rational-emotive behaviour therapy (REBT) on adolescents with conduct disorder (CD). *Journal of the Indian Academy of Applied Psychology, 35*, 103-111.	Wrong outcomes
Ladley, C. A. (1997). *Attributional training for anger and aggression in response to peer provocations among socially/emotionally disturbed children* [Unpublished doctoral dissertation]. Lehigh University.	Wrong outcomes
Laezer, K. L. (2015). Effectiveness of psychoanalytic psychotherapy and behavioral therapy treatment in children with attention deficit hyperactivity disorder and oppositional defiant disorder. *Journal of Infant, Child & Adolescent Psychotherapy, 14*(2), 111-128.	Children (0-11)
Lanctot, N., Hauth-Charlier, S., & Lemieux, A. (2015). Do depressive symptoms in justice-involved girls reduce the effects of a cognitive-behavioral program on their disruptive and delinquent behaviors? *International Journal of Social Welfare, 24*(2), 193-203.	Wrong outcomes
Landsverk, J. A., Burns, B. J., Stambaugh, L. F., & Reutz, J. A. R. (2009). Psychosocial interventions for children and adolescents in foster care: Review of research literature. *Child Welfare, 88*(1), 49-69.	Review
Langeveld, J. H., Gundersen, K. K., & Svartdal, F. (2012). Social competence as a mediating factor in reduction of behavioral problems. *Scandinavian Journal of Educational Research, 56*(4), 381-399.	Wrong outcomes
Larden, M., Hogstrom, J. & Langstrom, N. (2021). Effectiveness of an individual Cognitive-Behavioral intervention for serious, young male violent fffenders: Randomized controlled study with twenty-four-month follow-up. *Frontiers in Psychiatry, 12,* DOI: 10.3389/fpsyt.2021.670957	CU traits and/or antisocial behavior not measured at post
Larner, G. (2016). Family therapy with children and adolescents. *Australian and New Zealand Journal of Family Therapy, 37*(4), 439-442.	Review
Larson, J. D. (1992). Anger and aggression management techniques through the Think First curriculum. *Journal of Offender Rehabilitation, 18*(1-2), 101-117.	Wrong outcomes
Larson, J. D., Calamari, J. E., West, J. G., & Frevert, T. A. (1998). Aggression management with disruptive adolescents in the residential setting: Integration of a cognitive-behavioral component. *Residential Treatment for Children & Youth, 15(*4), 1-9.	Not an RCT or quasi study

Lau, A. S., Fung, J. J., Ho, L. Y., Liu, L. L., & Gudino, O. G. (2011). Parent training with high-risk immigrant Chinese families: a pilot group randomized trial yielding practice-based evidence. *Behavior Therapy, 42*(3), 413-426.	Children (0-11)
Lay, B., Blanz, B., & Schmidt, M. (2001). Effectiveness of home treatment in children and adolescents with externalizing psychiatric disorders. *European Child & Adolescent Psychiatry, 10*(5), I80-I90.	Children (0-11)
Läzer, K. L. (2012). Psychoanalytic Treatment with ADHD and ODD children - Frankfurt ADHD and ODD Effectiveness Study. *World Health Organization International Clinical Trials Registry Platform.* http://www.who.int/trialsearch/Trial2.aspx?TrialID=DRKS00003356	Protocol
Lee, M. Y., Greene, G. J., Hsu, K. S., Solovey, A., Grove, D., Fraser, J. S., Washburn, P., & Teater, B. (2009). Utilizing family strengths and resilience: integrative family and systems treatment with children and adolescents with severe emotional and behavioral problems. *Family Process, 48*(3), 395-416.	Children (0-11)
Leeman, L. W., Gibbs, J. C., & Fuller, D. (1993) Evaluation of a multicomponent group treatment program for juvenile delinquents. *Aggressive Behavior, 19*(4), 281-292.	Wrong outcomes
Leijten, P., Oyerbeek, G., & Janssens, Jmam (2012). Effectiveness of a parent training program in (pre)adolescence: Evidence from a randomized controlled trial. *Journal of Adolescence, 35*(4) 833-842.	Wrong outcomes
Lesage, A., Amor, L. B., Conrod, P., Geoffroy, M. C., Kackzorowski, J., Moga, C., Mombo, N., Renaud, J., Vasiliadis, H. M., Mongodin, A., & Gheorghiu, I. (2017). A review of best practices in five mental disorders in youth. *International Journal of Technology Assessment in Health Care, 33* (Suppl.), S237. https://doi.org/10.1017/S0266462317004184	Review
Letourneau, E. J., Henggeler, S. W., Borduin, C. M., Schewe, P. A., McCart, M. r., Chapman, J. E., & Saldana, L. (2009). Multisystemic therapy for juvenile sexual offenders: 1-year results from a randomized effectiveness trial. *Journal of Family Psychology, 23*(1) 89-102.	Wrong outcomes
Leyton, F., Olhaberry, M., Alvarado, R., Rojas, G., Duenas, L. A., Downing, G., & Steele, H. (2019). Video feedback intervention to enhance parental reflective functioning in primary caregivers of inpatient psychiatric children: Protocol for a randomized feasibility trial. *Trials, 20*(268), https://doi.org/10.1186/s13063-019-3310-y	Protocol
Li, K. K., Washburn, I., DuBois, D. L., Vuchinich, S., Ji, P., Brechling, V., Day, J., Beets, M. W., Acock, A. C., Berbaum, M., Snyder, F., & Flay, B. R. (2011). Effects of the positive action programme on problem behaviours in elementary school students: A matched-pair randomized control trial in Chicago. *Psychology and Health, 26*(2), 187-204.	Wrong population
Liau, A. K. (1999). *Evaluation of the peer helping component of a group treatment program for antisocial youth* [Unpublished doctoral dissertation]. Ohio State University.	Wrong outcomes
Liber, J. M., De Boo, G. M., Huizenga, H. & Prins, P. J. (2013). School-based intervention for childhood disruptive behavior in disadvantaged settings: A randomized controlled trial with and without active teacher support. *Journal of Consulting and Clinical Psychology, 81*(6), 975-987.	Children (0-11)

Liddle, H. A (2016).Multidimensional family therapy: Evidence base for transdiagnostic treatment outcomes, change mechanisms, and implementation in community settings. *Family Process, 55*(3), 558-576.	Review
Lieneman, C. C., Girard, E. I., Quetsch, L. B., & McNeil, C. B. (2020). Emotion regulation and attrition in parent-child interaction therapy. *Journal of Child and Family Studies, 29*(4), 978-996.	Children (0-11)
Lindsey, M. A., Romanelli, M., Ellis, M. L., Barker, E. d., Boxmeyer, C. L., & Lochman, J. E. (2019). The influence of treatment engagement on positive outcomes in the context of a school-based intervention for students with externalizing behavior problems. *Journal of Abnormal Child Psychology, 47*(9), 1437-1454.	Children (0-11)
Litschge, C. M., Vaughn, M. g., & McCrea, C. (2010). The empirical status of treatments for children and youth with conduct problems: An overview of meta-analytic studies. *Research on Social Work Practice, 20*(1), 21-35.	Review
Littell, J. H., Pigott, T. D., Nilsen, K. H., Green, S. J. & Montgomery, O. L. K. (2021). Multisystemic Therapy (R) for social, emotional, and behavioural problems in youth age 10 to 17: An updated systematic review and meta-analysis. *Campbell Systematic Reviews, 17*(4). 192	Review
Littell, J. H.; Winsvold, A.; Bjorndal, A.; Hammerstrom, K. T. (2007). Functional Family Therapy for families of youth (age 11-18) with behaviour problems. *Cochrane Database of systematic Reviews*, withdrawn.	Not retrievable
Liu, C., Wang, Y., Xuan, X., Xu, T., Wang, X. J., Tang, G. Z., Zhao, Y. M., & Wang, Y. F. (2009). A multi-center randomized controlled trial of social skills training among children with behavior problems. *Zhonghua Yi Xue Za Zhi, 89*(35), 2468-2471.	Children (0-11)
Livheim, F., Tengstrom, A., Andersson, G., Dahl, J., Bjorck, C., & Rosendahl, I. (2020). A quasi-experimental, multicenter study of acceptance and commitment therapy for antisocial youth in residential care. *Journal of Contextual Behavioral Science, 16*, 119-127.	Wrong outcomes
Lloyd, A., Brett, D., & Wesnes, K. (2010). Coherence training in children with attention-deficit hyperactivity disorder: Cognitive functions and behavioral changes. *Alternative Therapies in Health and Medicine, 16*(4), 34-42.	Wrong outcomes
Lochman, J. E., & Wells, K. C. (2004). The coping power program for preadolescent aggressive boys and their parents: outcome effects at the 1-year follow-up. *Journal of Consulting & Clinical Psychology, 72*(4), 571-578.	Children (0-11)
Lofholm, C. A., Olsson, T., Sundell, K., & Hansson, K. (2009). Multisystemic therapy with conduct-disordered young people: stability of treatment outcomes two years after intake. *Evidence and Policy, 5*(4), 373-397.	Wrong outcomes
Loucas, C., Pennant, M., Whittington, C., Naqvi, S., Sealey, C., Stockton, S., Kelvin, R., Fonagy, P., & Kendall, T. (2014). E-therapies for mental health problems in children and young people: A systematic review and focus group investigation. *Archives of Disease in Childhood, 99* (Suppl.), A58.	Review
Love, A. R., Mueller, C. W., Tolman, R. T., & Ka Powell, A. (2014). Frequency, level, and rate of improvement for treatment targets in a children's mental health community-based intensive in-home therapeutic setting. *Administration and Policy in Mental Health and Mental Health Services Research, 41*(4), 421-433.	Not an RCT or quasi study

Ludmer, J. A., Sanches, M., Propp, L., & Andrade, B. F. (2018). Comparing the multicomponent coping power program to individualized parent-child treatment for improving the parenting efficacy and satisfaction of parents of children with conduct problems. *Child Psychiatry & Human Development, 49*(1), 100-108.	Children (0-11)
Lui, J. H., Barry, C. T., & Marcus, D. K. (2019). A short-term intervention for adolescents with callous-unemotional traits and emotion-processing deficits. *Journal of Social and Clinical Psychology, 38*(6), 475-500.	Duplicate
Luk, E. S., Staiger, P., Mathai, J., Wong, L., Birleson, P., & Adler, R. (2001). Evaluation of outcome in child and adolescent mental health services: Children with persistent conduct problems. *Clinical Child Psychology and Psychiatry, 6(*1), 109-124.	Children (0-11)
Lundahl, B. W. (2007). Review: effects of behavioural parent training and cognitive-behavioural therapy on antisocial behaviour in children remain unclear. *Evidence Based Mental Health, 10*(2), 54.	Review
Madaus, M. R., & Ruberto, L. M. (2012). Application of self-modeling to externalizing and internalizing disorders. *Psychology in the Schools, 49*(1), 42-51.	Review
Mager, W., Milich, R., Harris, M. J., & Howard, A. (2005). Intervention groups for adolescents with conduct problems: Is aggregation harmful or helpful? *Journal of Abnormal Child Psychology, 33*(3), 349-362.	Wrong outcomes
Malone, R. P., Luebbert, J. F., Delaney, M. A., & Biesecker, K. A. (1997). Nonpharmacological response in hospitalized children with conduct disorder. *Journal of the American Academy of Child & Adolescent Psychiatry, 36*(2), 242-247.	Wrong intervention
Mann-Feder, V. R. (1996). Adolescents in therapeutic communities. *Adolescence, 31*(121), 17-28.	Wrong outcomes
Martinez, C. R., Jr. & Eddy, J. M. (2005). Effects of culturally adapted parent management training on Latino youth behavioral health outcomes. *Journal of Consulting & Clinical Psychology, 73*(5), 841-851.	Wrong outcomes
Martsch, M. D. (2005). A comparison of two group interventions for adolescent aggression: High process versus low process. *Research on Social Work Practice, 15*(1), 8-18.	Wrong outcomes
Masi, G., Milone, A., Paciello, M., Lenzi, F., Muratori, P., Manfredi, A., Polidori, L., Ruglioni, L., Lochman, J. E., & Muratori, F. (2014). Efficacy of a multimodal treatment for disruptive behavior disorders in children and adolescents: focus on internalizing problems. *Psychiatry Research, 219*(3), 617-624.	Children (0-11)
Masi, G., Manfredi, A., Milone, A., Muratori, P., Polidori, L., Ruglioni, L., & Muratori, F. (2011). Predictors of nonresponse to psychosocial treatment in children and adolescents with disruptive behavior disorders. *Journal of Child and Adolescent Psychopharmacology, 21*(1), 51-55.	Not an RCT or quasi study
Masi, G., Milone, A., Manfredi, A., Pari, C., Paziente, A., & Millepiedi, S. (2008). Conduct disorder in referred children and adolescents: Clinical and therapeutic issues. *Comprehensive Psychiatry, 49*(2), 146-153.	Not an RCT or quasi study
Mathys, C., Hyde, L. W., Shaw, D. S., & Born, M. (2013). Deviancy and normative training processes in experimental groups of delinquent and nondelinquent male adolescents. *Aggressive Behavior, 39*(1), 30-44.	Wrong outcomes

Mattos, L. A., Schmidt, A. T., Henderson, C. E., & Hogue, A. (2017). Therapeutic alliance and treatment outcome in the outpatient treatment of urban adolescents: The role of callous-unemotional traits. *Psychotherapy: Theory, Research, Practice, Training, 54*(2), 136-147.	CU and/or antisocial behavior not measured at post
Maughan, B., & Gardner, F. (2018). Multisystemic therapy not superior to management as usual for adolescent antisocial behaviour in an English trial. *The Lancet Psychiatry, 5*(2), 94-95.	Review
Maughan, D. R., Christiansen, E., Jenson, W. R., Olympia, D., & Clark, E. (2005). Behavioral parent training as a treatment for externalizing behaviors and disruptive behavior disorders: A meta-analysis. *School Psychology, 34*(3), 267-286.	Review
Maya, J., Hidalgo, V., Jiménez, L., & Lorence, B. (2020). Effectiveness of Scene-Based Psychodramatic Family Therapy (SB-PFT) in adolescents with behavioural problems. *Health & Social Care in the Community, 28*(2), 555-567.	Wrong outcomes
McCart, M. R., Sheidow, A. J. & Jaramillo, J. (2022). Evidence base update of psychosocial treatments for adolescents with disruptive behavior. *Journal of Clinical Child and Adolescent Psychology, 28,* DOI: 10.1080/15374416.2022.2145566	Review
McCart, M. R. (2007). *Reducing violence/victimization among assaulted urban youth* [Unpublished doctoral dissertation]. University of Wisconsin-Milwaukee.	Wrong outcomes
McCart, M. R., & Sheidow, A. J. (2016). Evidence-based psychosocial treatments for adolescents with disruptive behavior. *Journal of Clinical Child and Adolescent Psychology, 45*(5), 529-563.	Review
McClellan, J. M., & Werry, J. S. (2003). Evidence-based treatments in child and adolescent psychiatry: An inventory. *Journal of the American Academy of Child and Adolescent Psychiatry, 42*(12), 1388-1400.	Review
McClendon, J., Pollio, D. E., North, C. S., Reid, D., & Jonson-Reid, M. (2007). School-based groups for parents of children with emotional and behavioral disorders: Pilot results. *Families in Society, 88*(1), 124-129.	Children (0-11)
McQuillin, S. D., & McDaniel, H. L. (2021). Pilot randomized trial of brief school-based mentoring for middle school students with elevated disruptive behavior. *Annals of the New York Academy of Sciences, 1483*(1), 127-141.	Wrong outcomes
Merritts, A. (2016). A review of family therapy in residential settings. *Contemporary Family Therapy: An International Journal, 38*(1), 75-85.	Review
Midgley, N., O'Keeffe, S., French, L., & Kennedy, E. (2017). Psychodynamic psychotherapy for children and adolescents: An updated narrative review of the evidence base. *Journal of Child Psychotherapy, 43*(3), 307-329.	Review
Milani, A., Nikmanesh, Z., & Farnam, A. (2013). Effectiveness of Mindfulness-Based Cognitive Therapy (MBCT) in reducing aggression of individuals at the juvenile correction and rehabilitation center. *International Journal of High Risk Behaviors & Addiction, 2*(3), 126-131.	Wrong outcomes
Miller, L., Hlastala, S. A., Mufson, L., Leibenluft, E., Yenokyan, G., & Riddle, M. (2018). Interpersonal psychotherapy for mood and behavior dysregulation: Pilot randomized trial. *Depression and Anxiety, 35,* 574-582.	Wrong outcomes
Miller, L., Hlastala, S., Mufson, L., Leibenluft, E., Yenokyan, G., & Riddle, M. (2016). Interpersonal psychotherapy for mood and behavior dysregulation. *Journal of the American Academy of Child and Adolescent Psychiatry, 55*(10), S189-S190.	Wrong outcomes

Mitchell, P., Smedley, K., Kenning, C., McKee, A., Woods, D., Rennie, C. E., Bell, R. V., Aryamanesh, M., & Dolan, M. (2011). Cognitive behaviour therapy for adolescent offenders with mental health problems in custody. *Journal of Adolescence, 34*(3), 433-443.	Wrong outcomes
Mohamed, S. M., Marzouk, S. A., Ahmed, F. A., Nashaat, N. A. M., & Omar, R. A. E. A. T (2022). Cognitive behavioral program on aggression and self-concept among institutionalized children with conduct disorder. *Archives of Psychiatric Nursing, 39,* 84-90.	Wrong outcomes
Molleda, L., Estrada, Y., Lee, T. K., Poma, S., Teran, A. M., Tamayo, C. C., Bahamon, M., Tapia, M. I., Velazquez, M. R., Pantin, H., & Prado, G. (2017). Short-term effects on family communication and adolescent conduct problems: Familias unidas in Ecuador. *Prevention Science, 18*(7), 783-792.	Wrong outcomes
Moretti, M. M., Holland, R., & Peterson, S. (1994). Long term outcome of an attachment-based program for conduct disorder. *The Canadian Journal of Psychiatry / La Revue canadienne de psychiatrie, 39*(6), 360-370.	Not an RCT or quasi study
Moretti, M. M., & Obsuth, I. (2009). Effectiveness of an attachment-focused manualized intervention for parents of teens at risk for aggressive behaviour: The Connect Program. *Journal of Adolescence, 32*(6), 1347-1357.	Not an RCT or quasi study
Morris, E., Le Huray, C., Skagerberg, E., Gomes, R., & Ninteman, A. (2014). Families changing families: the protective function of multi-family therapy for children in education. *Clinical Child Psychology & Psychiatry, 19*(4), 617-32.	Children (0-11)
Moses, A. (2013). *Child parent relationship therapy for parents of children with disruptive behavior* [Unpublished doctoral dissertation]. Western Michigan University.	Children (0-11)
Moy, G. E., & Hazen, Amy (2018). A systematic review of the second step program. *Journal of School Psychology, 71*, 18-41.	Review
Muratori, P., Conversano, C., Levantini, V., Masi, G., Milone, A., Villani, S., Bogels, S., & Gemignani, A. (2021). Exploring the efficacy of a mindfulness program for boys with attention-deficit hyperactivity disorder and oppositional defiant disorder. *Journal of Attention Disorders, 25*(11), 1544-1553.	Children (0-11)
Muratori, P., Milone, A., Manfredi, A., Polidori, L., Ruglioni, L., Lambruschi, F., Masi, G., & Lochman, J. E. (2017). Evaluation of improvement in externalizing behaviors and callous-unemotional traits in children with disruptive behavior disorder: A 1-year follow up clinic-based study. *Administration and Policy in Mental Health and Mental Health Services Research, 44*(4), 452-462.	Children (0-11)
Murrihy, R. C., Drysdale, S. A., Dedousis-Wallace, A., Remond, L., McAloon, J., Ellis, D. M., Halldorsdottir, T., Greene, R. W. & Ollendick, T. H. (2022). Community-delivered collaborative and proactive solutions and parent management training for oppositional youth: A randomized trial. *Behavior Therapy,* DOI: 10.1016/j.beth.2022.10.005	Children (0-11)
Myeroff, R., Mertlich, G., & Gross, J. (1999). Comparative effectiveness of holding therapy with aggressive children. *Child Psychiatry & Human Development, 29*(4), 303-313.	Children (0-11)
Myers, W. C., Burton, P. R., Sanders, P. D., Donat, K. M., Cheney, J., Fitzpatrick, T. M., & Monaco, L. (2000). Project Back-on-Track at 1 year: A delinquency treatment	Wrong outcomes

program for early-career juvenile offenders. *Journal of the American Academy of Child & Adolescent Psychiatry, 39*(9), 1127-1134.	
Nayeri, M. F., Soltanifar, A., Moharreri, F., & Akbarzadeh, F. (2021). A randomized controlled trial of group reality therapy in attention deficit hyperactivity disorder and oppositional defiant disorder in adolescents. *Iranian Journal of Psychiatry and Behavioral Sciences, 15*(1), doi:10.5812/ijpbs.68643	Wrong outcomes
Neamtu, G. M., & David, O. A. (2016). Coaching emotional abilities in fostered adolescents through Rational Emotive and Cognitive-Behavioral Education: Efficacy and mechanisms of change of using therapeutic stories. *Journal of Evidence-Based Psychotherapies, 16*(1), 33-56.	Not an RCT or quasi study
Nelson-Gray, R. O., Keane, S. P., Hurst, R. M., Mitchell, J. t., Warburton, J. B., Chok, J. T., & Cobb, A. R. (2006). A modified DBT skills training program for oppositional defiant adolescents: Promising preliminary findings. *Behaviour Research and Therapy, 44*(12) 1811-1820.	Not an RCT or quasi study
Newcorn, J. H. (2013). Can children with callous and unemotional traits be treated successfully? *Journal of the American Academy of Child & Adolescent Psychiatry, 52*(12), 1257-1259.	Review
Newman, M., Fagan, C., & Webb, R. (2014). Innovations in practice: The efficacy of nonviolent resistance groups in treating aggressive and controlling children and young people: A preliminary analysis of pilot NVR groups in Kent. *Child and Adolescent Mental Health, 19*(2), 138-141.	Not an RCT or quasi study
Nickel, M. K., Krawczyk, J., Nickel, C., Forthuber, P., Kettler, C., Leiberich, P., Muehlbacher, M., Tritt, K., Mitterlehner, F. O., Lahmann, C., Rother, W. K., & Loew, T. H. (2005). Anger, interpersonal relationships, and health-related quality of life in bullying boys who are treated with outpatient family therapy: a randomized, prospective, controlled trial with 1 year of follow-up. *Pediatrics, 116*(2), 247-254.	Wrong outcomes
Nitsch, E., Hannon, G., Rickard, E., Houghton, S., & Sharry, J. (2015). Positive parenting: A randomised controlled trial evaluation of the Parents Plus Adolescent Programme in schools. *Child and Adolescent Psychiatry and Mental Health, 9*(43), doi:10.1186/s13034-015-0077-0	Wrong population
Nobel, E., Hoekstra, P. J., Agnes Brunnekreef, J., Messink-de Vries, D. E. H., Fischer, B., Emmelkamp, P. M. G., & van den Hoofdakker, B. J. (2019). Home-based parent training for school-aged children with attention-deficit/hyperactivity disorder and behavior problems with remaining impairing disruptive behaviors after routine treatment: a randomized controlled trial. *European Child & Adolescent Psychiatry, 29*(3), 395-408.	Children (0-11)
Nobel, E., Hoekstra, P. J., Agnes Brunnekreef, J., Messink-de Vries, D. E. H., Fischer, B., Emmelkamp, P. M. G., & van den Hoofdakker, B. J. (2020). Home-based parent training for school-aged children with attention-deficit/hyperactivity disorder and behavior problems with remaining impairing disruptive behaviors after routine treatment: a randomized controlled trial. *European Child & Adolescent Psychiatry, 29*(3), 395-408.	Duplicate
Nugent, W. R., Champlin, D., & Wiinimaki, L. (1997).The effects of anger control training on adolescent antisocial behavior. *Research on Social Work Practice, 7*(4), 446-462.	Wrong outcomes

Nuntavisit, L. & Porter, M. (2022). Mediating effects of discipline approaches on the relationship between parental mental health and adolescent antisocial behaviours: Retrospective study of a Multisystemic Therapy Intervention. *International Journal of Environmental Research and Public Health, 19*(20), DOI: 10.3390/ijerph192013418	Wrong outcomes
O'Keefe, N. K. (2019). *The implementation of Aggression Replacement Training (ART) with a high-risk, detained, pre-adjudicated juvenile offender population* [Unpublished doctoral dissertation]. Spalding University.	Wrong outcomes
O'Neill, M. L. (2001). *Adolescents with psychopathic characteristics in a substance abusing cohort: Predictors, correlates, and treatment process and outcome* [Unpublished doctoral dissertation]. MCP Hahnemann University.	CU and/or antisocial behavior not measured at post
O'Neill, M. L., Lidz, V., & Heilbrun, K. (2003). Adolescents with psychopathic characteristics in a substance abusing cohort: Treatment process and outcomes. *Law and Human Behavior*, *27*(3), 299-313.	Duplicate
Obsuth, I., Moretti, M. M., Holland, R., Braber, K., & Cross, Susan (2006). Conduct disorder: New directions in promoting effective parenting and strengthening parent-adolescent relationships. *Journal of the Canadian Academy of Child and Adolescent Psychiatry / Journal de l'Academie canadienne de psychiatrie de l'enfant et de l'adolescent*, *15*(1), 6-15.	Not an RCT or quasi study
Ogden, T. (2015). Evaluation of Functional Family Therapy (FFT) in Norway: one-year follow-up of a randomized trial investigating the effect of Functional Family Therapy (FFT) versus general family counseling by the family counseling service for externalizing disorders in adolescents. *World Health Organization International Clinical Trials Registry Platform.* https://trialsearch.who.int/Trial2.aspx?TrialID=ISRCTN58861782	Protocol
Ogden, T., & Hagen, K. A. (2006). Multisystemic treatment of serious behaviour problems in youth: Sustainability of effectiveness two years after intake. *Child and Adolescent Mental Health, 11*(3), 142-149.	Wrong outcomes
Ogden, T., & Hagen, K. A. (2008). Treatment effectiveness of Parent Management Training in Norway: A randomized controlled trial of children with conduct problems. *Journal of Consulting and Clinical Psychology*, *76*(4), 607-621.	Children (0-11)
Ogden, T., & Halliday-Boykins, C. A. (2004). Multisystemic treatment of antisocial adolescents in Norway: Replication of clinical outcomes outside of the US. *Child and Adolescent Mental Health*, *9*(2), 77-83.	Wrong outcomes
Ogwuche, C. H., Igbashal, V., & Chiahemba, M. D. (2018). Influence of parental support and monitoring on antisocial behaviour among secondary school students. *Journal of Education & Entrepreneurship*, *5*(1), 1-10. https://doi.org/10.26762/jee.2018.40000007	Not an RCT or quasi study
Olaghere, A., Wilson, D. B., & Kimbrell, C. S. (2021). Trauma-informed interventions for at-risk and justice-involved youth: A meta-analysis. *Criminal Justice and Behavior, 48*(9), 1261-1277.	Review
Ollefs, B., Von Schlippe, A., Omer, H., & Kriz, J. (2009). Adolescents with external behavior problems: Effects of parental coaching. *Familiendynamik: Systemische Praxis und Forschung, 34*(3), 256-265.	Wrong outcomes
Ollendick, T. H., Booker, J. A., Ryan, S., & Greene, R. W. (2018). Testing multiple conceptualizations of oppositional defiant disorder in youth. *Journal of Clinical Child and Adolescent Psychology, 47*(4), 620-633.	Wrong outcomes

Ollendick, T. H., Greene, R. W., Austin, K. E., Fraire, M. G., Halldorsdottir, T., Allen, K. B., Jarrett, M. A., Lewis, K. M., Whitmore Smith, M., Cunningham, N. R., Noguchi, R. J., Canavera, K., & Wolff, J. C. (2016). Parent management training and collaborative & proactive solutions: A randomized control trial for oppositional youth. *Journal of Clinical Child and Adolescent Psychology, 45*(5), 591-604.	Children (0-11)
Oparaduru, J. O. (2023). *Effectiveness of cognitive restructuring and self-control on proneness to maladaptive behaviour among selected senior secondary school students in IMO State, Nigeria* [Unpublished Doctoral Dissertation]. The University of Lagos.	Wrong outcomes
Osman, F. (2017). A Support Program for Somali-born Parents on Children's Behavioral Problems. *Pediatrics, 139*(3), 1-9.	Wrong outcomes
Osman, F., Flacking, R., Schon, U. K., & Klingberg-Allvin, M. (2017). A Support Program for Somali-born Parents on Children's Behavioral Problems. *Pediatrics, 139*(3), 1-9.	Duplicate
Ostberg, M., & Rydell, A. M. (2012). An efficacy study of a combined parent and teacher management training programme for children with ADHD. *Nordic Journal of Psychiatry, 66*(2), 123-130.	Children (0-11)
Owens, J. S., Johannes, L. M., & Karpenko, V. (2009). The relation between change in symptoms and functioning in children with ADHD receiving school-based mental health services. *School Mental Health: A Multidisciplinary Research and Practice Journal, 1*(4), 183-195.	Not an RCT or quasi study
Owens, J. S., Richerson, L., Beilstein, E. A., Crane, A., Murphy, C. E., & Vancouver, J. B. (2005). School-based mental health programming for children with inattentive and disruptive behavior problems: first-year treatment outcome. *Journal of Attention Disorders, 9*(1), 261-274.	Children (0-11)
Painter, K. R. (2008). *A quasi-experimental design: Multisystemic therapy as an alternative community-based treatment for youth with severe emotional disturbance* [Unpublished doctoral dissertation]. University of Texas at Arlington.	Children (0-11)
Painter, K. (2009). Multisystemic therapy as community-based treatment for youth with severe emotional disturbance. *Research on Social Work Practice, 19*(3), 314-324.	Wrong outcomes
Pandey, A., Hale, D., Das, S., Goddings, A. L., Blakemore, S. J., & Viner, R. M. (2018). Effectiveness of universal self-regulation-based interventions in children and adolescents: A systematic review and meta-analysis. *JAMA Pediatrics, 172*(6), 566-575.	Review
Pantin, H., Prado, G., Lopez, B., Huang, S., Tapia, M. I., Schwartz, S. J., Sabillon, E., Brown, C. H., & Branchini, J. (2009). A randomized controlled trial of familias unidas for Hispanic adolescents with behavior problems. *Psychosomatic Medicine, 71*(9), 987-995.	Wrong outcomes
Paquette, J., & Vitaro, F. (2014). Wilderness therapy, interpersonal skills and accomplishment motivation: Impact analysis on antisocial behavior and socio-professional status. *Residential Treatment for Children & Youth, 31*(3), 230-252.	Adults (18+)
Peckham, A. D., & Johnson, S. L. (2018). Cognitive control training for emotion-related impulsivity. *Behaviour Research and Therapy, 105*, 17-26.	Adults (18+)
Perez-Garcia, M., Sempere-Perez, J., Rodado-Martinez, J. V., Pina Lopez, D., Llor-Esteban, B., & Jimenez-Barbero, J. A. (2020). Effectiveness of multifamily therapy for adolescent disruptive behavior in a public institution: A randomized clinical trial.	Wrong outcomes

Children and Youth Services Review, 117, https://doi.org/10.1016/j.childyouth.2020.105289	
Pfiffner, L. J & McBurnett, K. (1997). Social skills training with parent generalization: Treatment effects for children with attention deficit disorder. *Journal of Consulting and Clinical Psychology, 65*(5), 749-757.	Children (0-11)
Piquero, A. R., Jennings, W. G., Farrington, D. P., Diamond, B., & Gonzalez, J. M. R. (2016). A meta-analysis update on the effectiveness of early self-control improvement programs to improve self-control and reduce delinquency. *Journal of Experimental Criminology, 12*(2), 249-264.	Review
Pol, T. M., Hendriks, V., Rigter, H., Cohn, M. D., Doreleijers, T. A., Domburgh, L., & Vermeiren, R. R. (2018). Multidimensional family therapy in adolescents with a cannabis use disorder: Long-term effects on delinquency in a randomized controlled trial. *Child and Adolescent Psychiatry and Mental Health, 12*(44), https://doi.org/10.1186/s13034-018-0248-x	Wrong outcomes
Porter, M., & Nuntavisit, L. (2016). An evaluation of multisystemic therapy with Australian families. *Australian and New Zealand Journal of Family Therapy, 37*(4), 443-462.	Not an RCT or quasi study
Prinz, R. J., Dumas, J. E., Smith, E. P., & Laughlin, J. E. (2000). The EARLY ALLIANCE prevention trial: A dual design to test reduction of risk for conduct problems, substance abuse, and school failure in childhood. *Controlled Clinical Trials, 21*(3), 286-302.	Children (0-11)
Prout, T. A., Rice, T., Chung, H., Gorokhovsky, Y., Murphy, S. & Hoffman, L. (2022). Randomized controlled trial of Regulation Focused Psychotherapy for children: A manualized psychodynamic treatment for externalizing behaviors. *Psychotherapy Research, 32*(5), 555-570.	Children (0-11)
Quinn, M. M. (2002). Changing antisocial behavior patterns in young boys: A structured cooperative learning approach. *Education and Treatment of Children, 25*(4), 380-395.	Children (0-11)
Reddy, S. D., Negi, L. T., Dodson-Lavelle, B., Ozawa-de Silva, B., Pace, T. W. W., Cole, S. P., Raison, C. L., & Craighead, L. W. (2013). Cognitive-based compassion training: A promising prevention strategy for at-risk adolescents. *Journal of Child and Family Studies, 22*(2), 219-230.	CU and/or antisocial behavior not measured at post
Rey, J., Denshire, E., Wever, C., & Apollonov, I. (1998). Three-year outcome of disruptive adolescents treated in a day program. *European Child & Adolescent Psychiatry, 7*(1), 42-48.	Wrong outcomes
Ribeiro da Silva, D., Rijo, D., Salekin, R. T., Paulo, M., Miguel, R., & Gilbert, P. (2020). Clinical change in psychopathic traits after the PSYCHOPATHY.COMP program: Preliminary findings of a controlled trial with male detained youth. *Journal of Experimental Criminology*, https://doi.org/10.1007/s11292-020-09418-x	CU and/or antisocial behavior not measured at post
Rickson, D. J., & Watkins, W. G. (2003). Music therapy to promote prosocial behaviors in aggressive adolescent boys-A pilot study. *Journal of Music Therapy, 40*(4), 283-301.	Wrong outcomes
Riedinger, V., Pinquart, M., & Teubert, D. (2017). Effects of systemic therapy on mental health of children and adolescents: A meta-analysis. *Journal of Clinical Child and Adolescent Psychology, 46*(6), 880-894.	Review

Riise, Eili N., Wergeland, G. J. H., Njardvik, U., & Ost, L. G. (2021). Cognitive behavior therapy for externalizing disorders in children and adolescents in routine clinical care: A systematic review and meta-analysis. *Clinical Psychology Review, 83,* https://doi.org/10.1016/j.cpr.2020.101954	Review
Rijo, D., Ribeiro da Silva, D., Brazao, N., Paulo, M., Ramos, M. R., Castilho, P., Vagos, P. & Gilbert, P. (2022). Promoting a compassionate motivation in detained youth: A secondary analysis of a controlled trial with the PSYCHOPATHY.COMP program. *Personality Disorders: Theory, Research & Treatment, 28*. DOI: 10.1037/per0000594	CU traits and/or antisocial behavior not measured at post
Rijo, D., Brazao, N., Barroso, R., da Silva, D. R., Vagos, P., Vieira, A., Lavado, A., & Macedo, A. M. (2016). Mental health problems in male young offenders in custodial versus community based-programs: Implications for juvenile justice interventions. *Child and Adolescent Psychiatry and Mental Health, 10*(40), doi:10.1186/s13034-016-0131-6	No treatment tested/given
Ritchie, M. B., Neufeld, R. W., Yoon, M., Ashley, M. & Derek, G. (2022). Predicting youth aggression with empathy and callous unemotional traits: A meta-analytic review. *Clinical Psychology Review, 98,* 1-14.	Review
Roberts, B. C. (2014). *A description and initial assessment of a behavior management intervention technique used in a program for behaviorally and emotionally troubled students* [Unpublished doctoral dissertation]. University of Massachusetts Amherst.	Not an RCT or quasi study
Roberts, H., & Honzel, N. (2020). The effectiveness of equine-facilitated psychotherapy in adolescents with serious emotional disturbances. *Anthrozoos, 33*(1), 133-144.	Wrong outcomes
Robinson, T., Smith, Stephen W., & Miller, M. (2002). Effect of a cognitive-behavioral intervention on responses to anger by middle school students with chronic behavior problems. *Behavioral Disorders, 27*(3), 256-271.	Wrong outcomes
Rogers, J. C. & Broome, M. R. (2020). Effectiveness of multisystemic therapy for adolescent antisocial behaviour: Follow-up findings from the START trial. *The Lancet Psychiatry, 7*(5), 375-376.	Review
Rohde, P., Clarke, G. N., Mace, D. E., Jorgensen, J. S., & Seeley, J. R. (2004). An efficacy/effectiveness study of cognitive-behavioral treatment for adolescents with comorbid major depression and conduct disorder. *Journal of the American Academy of Child & Adolescent Psychiatry, 43*(6), 660-668.	Wrong outcomes
Romaine, C. L. R., Kemp, K., Giallella, C. L., Goldstein, N. E. S., Serico, J., & Kelley, S. (2018). Can we hasten development? Effects of treatment on psychosocial maturity. *International Journal of Offender Therapy and Comparative Criminology, 62*(9), 2857-2876.	Wrong outcomes
Rothwell, E., Piatt, J., & Mattingly, K. (2006). Social competence: Evaluation of an outpatient recreation therapy treatment program for children with behavioral disorders. *Therapeutic Recreation Journal, 40*(4), 241-254.	Not an RCT or quasi study
Roux, B., Perez-Pena, M. & Philippot, P. (2021). A mindfulness-based intervention for adolescents with behavior disorders: Controlled trial with partial randomization. *Mindfulness, 12*(11), 2794-2809.	Wrong outcomes
Roux, B., & Philippot, P. (2020). A mindfulness-based program among adolescent boys with behavior disorders: A quasi-experimental study. *Journal of Child and Family Studies, 29*(8), 2186-2200.	Wrong outcomes

Rowlands, A., Fisher, M., Mishra, J., Nahum, M., Brandrett, B., Reinke, M., Caldwell, M., Kiehl, K. A., & Vinogradov, S. (2020). Cognitive training for very high risk incarcerated adolescent males. *Frontiers in Psychiatry, 11*(225), doi: 10.3389/fpsyt.2020.00225	Not an RCT or quasi study
Rush, K. S., Golden, M. E., Mortenson, B. P., Albohn, D., & Horger, M. (2017). The effects of a mindfulness and biofeedback program on the on- and off-task behaviors of students with emotional behavioral disorders. *Contemporary School Psychology, 21*(4), 347-357.	Children (0-11)
Ryan, E. P., & Redding, R. E. (2004). A review of mood disorders among juvenile offenders. *Psychiatric Services, 55*(12), 1397-1407.	Review
Sachdeva, S., Goldman, G., Mustata, G., Deranja, E., & Gregory, R. J. (2013). Naturalistic outcomes of evidence-based therapies for borderline personality disorder at a university clinic: A quasi-randomized trial. *Journal of the American Psychoanalytic Association, 61*(3), 578-584.	Adults (18+)
Sachs, J. J., & Miller, S. R. (1992). The impact of a wilderness experience on the social interactions and social expectations of behaviorally disordered adolescents. *Behavioral Disorders, 17*(2), 89-98.	Wrong intervention
Salari, M. (2018). Effectiveness of an interventional program on aggressive behaviors in adolescents aged 13 to 15. *World Health Organization International Clinical Trials Registry Platform.* http://www.who.int/trialsearch/Trial2.aspx?TrialID=IRCT20180813040780N1	Wrong outcomes
Salari, R., Ralph, A., & Sanders, M. R. (2014). An efficacy trial: Positive parenting program for parents of teenagers. *Behaviour Change, 31*(1), 34-52.	Wrong outcomes
Salekin, R. T. & Andershed, H. (2022). Psychopathic personality, and its dimensions in the prediction of negative outcomes: Do they offer incremental value above and beyond common risk factors? Introduction to the special section. *Journal of Criminal Justice, 80,* DOI: 10.1016/j.jcrimjus.2022.101914	Review
Salekin, R. T., Tippey, J. g., & Allen, A. D. (2012). Treatment of conduct problem youth with interpersonal callous traits using mental models: Measurement of risk and change. *Behavioral Sciences & the Law, 30*(4), 470-486.	Not an RCT or quasi study
Salmanian, M., & Tehran Univeristy of Medical Sciences, Psychiatry and Psychology Research Center. (2013). A clinical trial for comparison of reducing conduct disorder symptoms by spiritual psychotherapy package in adolescents with conduct disorder. *Iranian Registry of Clinical Trials.* https://en.irct.ir/trial/9735	Wrong outcomes
Salmon, K., Dadds, M. R., Allen, J., & Hawes, D. J. (2009). Can emotional language skills be taught during parent training for conduct problem children? *Child Psychiatry & Human Development, 40*(4), 485-498.	Children (0-11)
Salzer, S., Cropp, C., Jaeger, U., Masuhr, O., & Streeck-Fischer, A. (2014). Psychodynamic therapy for adolescents suffering from co-morbid disorders of conduct and emotions in an in-patient setting: A randomized controlled trial. *Psychological Medicine, 44*(10), 2213-2222.	Wrong outcomes
Sancassiani, F., Lecca, M. E., Pintus, E., Moro, M. F., Caria, R., Minerba, L., Mela, Q., Nardi, A. E., Machado, S., d'Aloja, E., Preti, A., & Carta, M. G. (2019). Could an innovative training program including contact sports and counseling help young people with traits of psychopathy and a history of school dropout? *Clinical Practice and Epidemiology in Mental Health, 15,* 49-57.	Adults (18+)

Santisteban, D. A., Coatsworth, J. D., Perez-Vidal, A., Kurtines, W. M., Schwartz, S. J., LaPerriere, A., & Szapocznik, J. (2003). Efficacy of brief strategic family therapy in modifying Hispanic adolescent behavior problems and substance use. *Journal of Family Psychology, 17*(1), 121-133.	Wrong outcomes
Sasaki, Y., Usami, M., Sasaki, S., Sunakawa, H., Toguchi, Y., Tanese, S., Saito, K., Shinohara, R., Kurokouchi, T., Sugimoto, K., Hakoshima, Y., Inazaki, K., Yoshimura, Y., Mizumoto, Y. & Okada, T. (2021). Case-control study on clinical characteristics of child and adolescent psychiatric outpatients with child-to-parent violence. *BMJ Open,* DOI: 10.1136/bmjopen-2020-048222	Children (0-11)
Sawrikar, V., Hawes, D. J. & Dadds, M. R. (2022). Family therapy interventions for psychopathy. In J. E. Vitale (Ed.) *The Complexity of Psychopathy* (519-544). Springer. DOI: 10.1007/978-3-030-83156-1_19	Book/chapter
Sawyer, A. M., Borduin, C. M., & Dopp, Alex R. (2015). Long-term effects of prevention and treatment on youth antisocial behavior: A meta-analysis. *Clinical Psychology Review, 42*, 130-144.	Review
Sayers, J. (2021). *Factors associated with reward and punishment responsivity in children and young people with callous unemotional traits* [Unpublished doctoral dissertation]. University of Liverpool.	Review
Scahill, L., McDougle, C. J., Aman, M. G., Johnson, C., Handen, B., Bearss, K., Dziura, J., Butter, E., Swiezy, N. G., Arnold, L. E., Stigler, K. A., Sukhodolsky, D. D., Lecavalier, L., Pozdol, S. L., Nikolov, R., Hollway, J. A., Korzekwa, P., Gavaletz, A., Kohn, A. E., Koenig, K., Grinnon, S., Mulick, J. A., Yu, S., Vitiello, B., & Research Units on Pediatric Psychopharmacology Autism, Network. Effects of risperidone and parent training on adaptive functioning in children with pervasive developmental disorders and serious behavioral problems. *Journal of the American Academy of Child & Adolescent Psychiatry, 51*(2):136-146.	Somatic or developmental disorder
Schippers, E. E., Hoogsteder, L. M., & Stams, Gjjm. (2020). Responsive Aggression Regulation Therapy (Re-ART) improves executive functioning in adolescents and young adults with severe aggression problems: A pilot study. *Journal of Forensic Sciences, 65*(6), 2058-2064.	Wrong outcomes
Schuppert, H., Timmerman, M. E., Bloo, J., van Gemert, T. G., Wiersema, H. M., Minderaa, R. B., Emmelkamp, P. M., & Nauta, M. H. (2012). Emotion regulation training for adolescents with borderline personality disorder traits: A randomized controlled trial. *Journal of the American Academy of Child & Adolescent Psychiatry, 51*(12), 1314-1323.	Wrong outcomes
Schwartzberg, A. Z. (1999). Risk factors for adolescent violence: Implications for prevention and treatment. *International Journal of Adolescent Medicine and Health, 11*(3-4), 429-437.	No treatment tested/given
Schwenck, C., Schneider, W., & Reichert, A. (2016). Universal parent training as a supplement to inpatient psychiatric treatment for children and adolescents. *European Child and Adolescent Psychiatry, 25*(8), 879-889.	Wrong outcomes
Seagram, B. M. C. (1998). *The efficacy of solution-focused therapy with young offenders* [Unpublished doctoral dissertation]. York University.	Not an RCT or quasi study
Sexton, T., & Turner, C. W. (2010). The effectiveness of functional family therapy for youth with behavioral problems in a community practice setting. *Journal of Family Psychology, 24*(3), 339-348.	Wrong outcomes

Shahidullah, J. D. (2016). *Integrated behavioral health treatment for ADHD referrals in pediatric primary care: Clinical improvement, acceptability, adherence, and cost of care* [Unpublished doctoral dissertation]. Michigan State University.	Children (0-11)
Shechtman, Z. (2006). The contribution of bibliotherapy to the counseling of aggressive boys. *Psychotherapy Research, 16*(5), 645-651.	Wrong outcomes
Sheidow, A. J., McCart, M. R. & Drazdowski, T. K. (2022). Family-based treatments for disruptive behavior problems in children and adolescents: An updated review of rigorous studies (2014-April 2020). *Journal of Marital & Family Therapy, 48*(1), 56-82.	Review
Sheidow, A. J., Zajac, K., Chapman, J. E., McCart, M. R., & Drazdowski, T. K. (2021). Randomized controlled trial of an integrated family-based treatment for adolescents presenting to community mental health centers. *Community Mental Health Journal, 57*(6), 1094-1110.	Wrong outcomes
Sheidow, A. J., McCart, M. R., & Davis, M. (2016). Multisystemic therapy for emerging adults with serious mental illness and justice involvement. *Cognitive and Behavioral Practice, 23*(3), 356-367.	Not an RCT or quasi study
Shenk, C. E., Dorn, L. D., Kolko, D. J., Rausch, J. R., & Insana, S. P. (2014). Prior exposure to interpersonal violence and long-term treatment response for boys with a disruptive behavior disorder. *Journal of Traumatic Stress, 27*(5), 585-592.	Children (0-11)
Sibley, M. H., Graziano, P. A., Kuriyan, A. B., Coxe, S., Pelham, W. E., Rodriguez, L., Sanchez, F., Derefinko, K., Helseth, S., & Ward, A. (2016). Parent-teen behavior therapy + motivational interviewing for adolescents with ADHD. *Journal of Consulting and Clinical Psychology, 84*(8), 699-712.	Wrong outcomes
Sibley, M. H. (2013). *Supporting Teens' Academic Needs Daily (STAND): A parent-adolescent collaborative intervention for ADHD* [Unpublished doctoral dissertation]. State University of New York at Buffalo.	Wrong outcomes
Sibley, M. H., & Pelham, William E. (2013). Supporting teens' academic needs daily (STAND): A parent-adolescent collaborative intervention for ADHD. *Journal of Psychopathology and Behavioral Assessment, 35,* 436-449.	Wrong outcomes
Simons Morton, B., Haynie, D., Saylor, K., Crump, A. D., & Chen, R. (2005). The effect of the Going Places Program on early adolescent substance use and antisocial behavior. *Prevention Science, 6*(3), 187-197.	Wrong population
Simpson, S., Mercer, S., Simpson, R., Lawrence, M., & Wyke, S. (2018). Mindfulness-based interventions for young offenders: A scoping review. *Mindfulness, 9*(5), 1330-1343.	Review
Simpson, T. P., Frick, P. J., Kahn, R. E., & Evans, L. J. (2013). Therapeutic alliance in justice-involved adolescents undergoing mental health treatment: The role of callous-unemotional traits. *The International Journal of Forensic Mental Health, 12*(2), 83-92.	Not an RCT or quasi study
Sinclair, I., Parry, E., Biehal, N., Fresen, J., Kay, C., Scott, S., & Green, J. (2016). Multi-dimensional treatment foster care in England: Differential effects by level of initial antisocial behaviour. *European Child & Adolescent Psychiatry, 25*(8), 843-852.	Wrong outcomes
Singal, S. (2009). *The efficacy of psychodrama in the treatment of oppositional and defiant adolescents* [Unpublished doctoral dissertation]. McMaster University.	Wrong outcomes

Slot, N., Jagers, H. D., & Dangel, R. F. (1992). Cross-cultural replication and evaluation of the Teaching Family Model of community-based residential treatment. *Behavioral Residential Treatment, 7*(5), 341-354.	Not an RCT or quasi study
Smith, J. D., Knoble, N. B., Zerr, A. A., Dishion, T. J., & Stormshak, E. A. (2014). Family check-up effects across diverse ethnic groups: Reducing early-adolescence antisocial behavior by reducing family conflict. *Journal of Clinical Child and Adolescent Psychology, 43*(3), 400-414.	Wrong outcomes
Smith, K. (2018). *Take the lead: An evaluation of Monterey county SPCA's dog-training program with at-risk youth* [Unpublished doctoral dissertation]. Palo Alto Univeristy.	Wrong intervention
Snyder, K. V., Kymissis, P., & Kessler, K. (1999). Anger management for adolescents: Efficacy of brief group therapy. *Journal of the American Academy of Child and Adolescent Psychiatry, 38*(11), 1409-1416.	Wrong outcomes
Snyder, K. V. (1999). *Anger management for adolescents: Efficacy of a short-term cognitive-behavioral intervention* [Unpublished doctoral dissertation]. Fordham University.	Wrong outcomes
Solan, M., Klomek, A. B., Ankori, G., Bloch, A., Apter, A., & Plishty, S. (2020). Impact of a new parent behavioral-schema training on children with ADHD: A pragmatic control trial. *Journal of Attention Disorders,* doi:10.1177/1087054720959711	Children (0-11)
Spielman, D. A., & Staub, E. (2000). Reducing boys' aggression: Learning to fulfill basic needs constructively. *Journal of Applied Developmental Psychology, 21*(2), 165-181.	Wrong outcomes
Spijkers, W., Jansen, D. E., de Meer, G., & Reijneveld, S. A. (2010). Effectiveness of a parenting programme in a public health setting: a randomised controlled trial of the positive parenting programme (Triple P) level 3 versus care as usual provided by the preventive child healthcare (PCH). *BMC Public Health, 10*(131), http://www.biomedcentral.com/1471-2458/10/131	Protocol
Spijkers, W., Jansen, D. E. M. C., & Reijneveld, S. A. (2013). Effectiveness of Primary Care Triple P on child psychosocial problems in preventive child healthcare: A randomized controlled trial. *BMC Medicine, 11*(240), http://www.biomedcentral.com/1741-7015/11/240	Children (0-11)
Spoth, R. L., Redmond, C., & Shin, C. (2000). Reducing adolescents' aggressive and hostile behaviors - Randomized trial effects of a brief family intervention 4 years past baseline. *Archives of Pediatrics & Adolescent Medicine, 154*(12), 1248-1257.	Wrong population
Sprague, A., & Thyer, B. A. (2003). Psychosocial treatment of oppositional defiant disorder: A review of empirical outcome studies. *Social Work in Mental Health, 1*(1), 63-72.	Review
Sprenkle, D. H. (2012). Intervention research in couple and family therapy: A methodological and substantive review and an introduction to the special issue. *Journal of Marital and Family Therapy, 38*(1), 3-29.	Review
Squires, G., & Caddick, K. (2012). Using group cognitive behavioural therapy intervention in school settings with pupils who have externalizing behavioural difficulties: An unexpected result. *Emotional and Behavioural Difficulties, 17*(1), 25-45.	Wrong outcomes

Sroka, I. M., Isemann, S. D., & Walther, E. (2017). With or without them: Improving self-control in juvenile offenders. *Basic and Applied Social Psychology, 39*(5), 277-286.	Adults (18+)
Stadler, C., Konrad, K., Popma, A., Kirchner, M. & Freitag, C. (2019). S.19.04 Start now: a program to improve emotion regulation in female patients with disruptive behaviour disorder. *European Neuropsychopharmacology, 29,* DOI: 10.1016/j.euroneuro.2019.09.045	Duplicate
Stadler, C., Konrad, K., Popma, A., Kirchner, M. & Freitag, C. (2019). S.19.04 Start now: a program to improve emotion regulation in female patients with disruptive behaviour disorder. *European Neuropsychopharmacology, 29,* DOI: 10.1016/j.euroneuro.2019.09.045	Wrong outcomes
Stage, S. A., Jackson, H. G., Jensen, M. J., Moscovitz, K. K., Bush, J. W., Violette, H. D., Thurman, S. O., Olson, E., Bain, N. & Pious, C. (2008). A validity study of functionally-based behavioral consultation with students with emotional/behavioral disabilities. *School Psychology Quarterly, 23*(3), 327-353.	Children (0-11)
Steeger, C. M. (2014). *Combined cognitive and parent training interventions for adolescents with adhd and their mothers: A randomized, controlled trial* [Unpublished Doctoral Dissertation].	Wrong outcomes
Stefanini, M. C., Martino, A., Bacci, B. & Tani, F. (2016). The effect of animal-assisted therapy on emotional and behavioral symptoms in children and adolescents hospitalized for acute mental disorders. *European Journal of Integrative Medicine, 8*(2), 81-88.	Wrong outcomes
Stephanik, C. E. (1997). *Effects of a parent/adolescent training program on adolescent conduct disorders: A comparison across four intervention conditions* [Unpublished Doctoral Dissertation].	Wrong outcomes
Streeck-Fischer, A. (2012). Borderline and complex traumatised adolescents. *Neuropsychiatrie de l'Enfance et de l'Adolescence,* DOI: 10.1016/j.neurenf.2012.05.338	Supplement with no publication
Streeck-Fischer, A. & Cropp, C. (2012). Evaluation of inpatient psychodynamic psychotherapy for adolescents who suffer from mixed disorders of conduct and emotions (F92, ICD-10) with OPD-CA axis structure. *Neuropsychiatrie de l'Enfance et de l'Adolescence*, DOI: 10.1016/j.neurenf.2012.05.210	Duplicate
Sukhodolsky, D. G., Vander Wyk, B. C., Eilbott, J. A., McCauley, S. A., Ibrahim, K., Crowley, M. J., Pelphrey, K. A. (2016). Neural mechanisms of Cognitive-Behavioral Therapy for aggression in children and adolescents: Design of a randomized controlled trial within the National Institute for Mental Health Research Domain Criteria Construct of frustrative non-reward. *Journal of Child & Adolescent Psychopharmacology, 26*(1), 38-48.	Could not contact author
Sukhodolsky, D. G., Gorman, B. S., Scahill, L., Findley, D. & McGuire, J. (2013). Exposure and response prevention with or without parent management training for children with obsessive-compulsive disorder complicated by disruptive behavior: A multiple-baseline across-responses design study. *Journal of Anxiety Disorders, 27*(3), 298-305.	Wrong outcomes
Sundell, K., Hansson, K., Lofholm, C. A., Olsson, T., Gustle, L. H. & Kadesjo, C. (2008). The transportability of multisystemic therapy to Sweden: Short-term results	Wrong outcomes

from a randomized trial of conduct-disordered youths. *Journal of Family Psychology, 22*(4), 550-560.	
Swart, J. & Apsche, J. (2014). Mode deactivation therapy meta-analysis: Reanalysis and interpretation. *International Journal of Behavioral Consultation and Therapy, 9*(2), 16-21.	Review
Swart, J. & Apsche, J. (2014). Family mode deactivation therapy (FMDT): A randomized controlled trial for adolescents with complex issues. *International Journal of Behavioral Consultation and Therapy, 9*(1), 14-22.	Wrong outcomes
Swart, J. & Apsche, J. (2014). A comparative treatment efficacy study of conventional therapy and mode deactivation therapy (MDT) for adolescents with conduct disorders, mixed personality disorders, and experiences of childhood trauma. *International Journal of Behavioral Consultation and Therapy, 9*(1), 23-29.	Wrong outcomes
Swart, J. & Apsche, J. (2014). A comparative study of mode deactivation therapy (MDT) as an effective treatment of adolescents with suicidal and non-suicidal self-injury behaviors. *International Journal of Behavioral Consultation and Therapy, 9*(3), 47-52.	Wrong outcomes
Swogger, M. T., Conner, K. R., Caine, E. D., Trabold, N., Parkhurst, M. N., Prothero, L. M. & Maisto, S. A. (2016). A test of core psychopathic traits as a moderator of the efficacy of a brief motivational intervention for substance-using offenders. *Journal of Consulting & Clinical Psychology, 84*(3), 248-258.	Adults (19+)
Taiwo, A. O. & Osinowo, H. O. (2011). Treating anti-social behavior in adolescents: A comparison of multi-modal and psychodynamic oriented group interventions. *Journal of Psychology in Africa, 21*(4), 627-632.	Wrong outcomes
Tan. J. X. & Fajardo, M. L. R. (2017). Efficacy of multisystemic therapy in youths aged 10-17 with severe antisocial behaviour and emotional disorders: Systematic review. *London Journal of Primary Care, 9*(6), 95-103.	Review
Tarver, J., Daley, D., Lockwood, J. & Sayal, K. (2014). Are self-directed parenting interventions sufficient for externalising behaviour problems in childhood? A systematic review and meta-analysis. *European Child & Adolescent Psychiatry, 23*(12), 1123-1137.	Review
Taubner, S., Volkert, J., Gablonski, T. C. & Rossouw, T. (2017). Mentalization-based treatment for adolescents with borderline personality disorder-Concept and efficacy. *Praxis der Kinderpsychologie und Kinderpsychiatrie, 66*(6), 423-434.	Review
Taubner, S., & Heidelberg University (2016). Mentalization-based training for adolescents with conduct disorder (MBT-CD). *U.S National Library of Medicine Clinical Trials*. https://clinicaltrials.gov/ct2/show/NCT02988453	CU and/or antisocial behavior not measured at post
Taylor, L., Leary, K., Boyle, A., Bigelow, K., Henry, T. & DeRosier, M. (2015). Parent training and adolescent social functioning: A brief report. *Journal of Child & Family Studies, 24*(10), 3030-3037.	Wrong outcomes
Taylor, T. K., Eddy, J. & Biglan, A. (1999). Interpersonal skills training to reduce aggressive and delinquent behavior: Limited evidence and the need for an evidence-based system of care. *Clinical Child and Family Psychology Review*, *2*(3), 169-182.	Review
Teeter, P. A., Rumsey, R., Natoli, L., Naylor, D. & Smith, R. (2000). Therapeutic interventions to increase social competence in teens with impulse control deficits. *Journal of Psychotherapy in Independence Practice, 1*(4), 49-70.	Wrong outcomes

Tengstrom, A. (2012). Study of prevention programs for parents of adolescents with antisocial behavior. *World Health Organization International Clinical Trials Registry Platform.* http://www.who.int/trialsearch/Trial2.aspx?TrialID=ISRCTN76141538	Wrong outcomes
Thastum, M., Johnsen, D. B., Silverman, W. K., Jeppesen, P., Heyne, D. A. & Lomholt, J. J. (2019). The Back2School modular cognitive behavioral intervention for youths with problematic school absenteeism: study protocol for a randomized controlled trial. *Trials, 20*(1), 29. DOI: 10.1186/s13063-018-3124-3	Protocol
Thøgersen, D. M., Bjørnebekk, G., Scavenius, C. & Elmose, M. (2021). Callous-unemotional traits do not predict functional family therapy outcomes for adolescents with behavior problems. *Frontiers in Psychology, 11,* DOI: 10.3389/fpsyg.2020.537706	Not an RCT or quasi study
Thongseiratch, T., Leijten, P. & Melendez-Torres, G. J. (2020). Online parent programs for children's behavioral problems: a meta-analytic review. *European Child & Adolescent Psychiatry*, *29*(11), 1555-1568.	Review
Thorell, L. B. (2009). The Community Parent Education Program (COPE): Treatment effects in a clinical and a community-based sample. *Clinical Child Psychology and Psychiatry, 14*(3), 373-387.	Children (0-11)
Thorne, I. (2020). *The efficacy of animal-assisted therapy on reducing aggression and non-compliance in children with behavioral and psychiatric disorders: A school-based intervention* [Unpublished Doctoral Dissertation]. Widener University.	Children (0-11)
Thurston, A., Miller, S., Dunne, L., Lazenbatt, A., Gildea, A., Stepien, D. & Tapsell, D. (2015). Research protocol: A randomized controlled trial of functional family therapy: An Early Intervention Foundation (EIF) partnership between Croydon Council and Queen's University Belfast. *International Journal of Educational Research, 70,* 47-56.	Protocol
Tolan, P. H., Gorman-Smith, D., Henry, D. & Schoeny, M. (2009). The benefits of booster interventions: evidence from a family-focused prevention program. *Prevention Science, 10*(4), 287-297.	Children (0-11)
Tuente, S. K., Bogaerts, S., Bulten, E., Keulen-de Vos, M., Vos, M., Bokern, H., van Ijzendoorn, S., Geraets, C. N. W. & Veling, W. (2020). Virtual Reality Aggression Prevention Therapy (VRAPT) versus waiting list control for forensic psychiatric inpatients: A multicenter randomized controlled trial. *Journal of Clinical Medicine, 9*(7), DOI: 10.3390/jcm9072258	Adults (19+)
Uliaszek, A. A., Wilson, S., Mayberry, M., Cox, K. & Maslar, M. (2014). A pilot intervention of multifamily dialectical behavior group therapy in a treatment-seeking adolescent population: Effects on teens and their family members. *The Family Journal, 22*(2), 206-215.	Not an RCT or quasi study
Umbach, R., Raine, A. & Leonard, N. R. (2018). Cognitive decline as a result of incarceration and the effects of a CBT/MT Intervention: A cluster-randomized controlled trial. *Criminal Justice and Behavior*, 45(1), 31-55.	Wrong outcomes
University of Sheffield (2019). A trial of an online problem-solving intervention for aggression. *U.S National Library of Medicine Clinical Trials.* https://clinicaltrials.gov/ct2/show/NCT04130360	Wrong outcomes
Uzunoğlu, G., Arabacı, L.B. & Dusunen A. (2017). Davranım Bozukluğu Tanılı Ergenlere Verilen Psikoeğitimin Öfke Yönetimine Etkisi. *Journal of Psychiatry & Neurological Sciences, 30*(4), 344-353.	Wrong outcomes

van de Wiel, N. M., Matthys, W., Cohen-Kettenis, P. T., Maassen, G. H., Lochman, J. E. & van Engeland, H. (2007). The effectiveness of an experimental treatment when compared to care as usual depends on the type of care as usual. *Behavior Modification, 31*(3), 298-312.	Children (0-11)
van der Oord, S., Prins, P. J. M., Oosterlaan, J. & Emmelkamp, P. M. G. (2007). Does brief, clinically based, intensive multimodal behavior therapy enhance the effects of methylphenidate in children with ADHD? *European Child and Adolescent Psychiatry, 16*(1), 48-57.	Children (0-11)
van der Pol, T. M., Hoeve, M., Noom, M. J., Stams, G. J. J., Doreleijers, T. A., van Domburgh, L. & Vermeiren, R. R. (2017). Research review: The effectiveness of multidimensional family therapy in treating adolescents with multiple behavior problems-A meta-analysis. *Journal of Child Psychology and Psychiatry, 58*(5), 532-545.	Review
van der Velden, F., Brugman, D., Boom, J. & Koops, W. (2010). Effects of EQUIP for educators on students' self-serving cognitive distortions, moral judgment, and antisocial behavior. *Journal of Research in Character Education, 8*(1), 77-95.	Wrong population
Van Holen, F., Vanderfaeillie, J., Omer, H. & Vanschoonlandt, F. (2018). Training in nonviolent resistance for foster parents. *Research on Social Work Practice, 28*(8), 931-942.	Children (0-11)
van Manen, T. G., Prins, P. J. & Emmelkamp, P. M. (2004). Reducing aggressive behavior in boys with a social cognitive group treatment: results of a randomized, controlled trial. *Journal of the American Academy of Child & Adolescent Psychiatry, 43*(12), 1478-1487.	Children (0-11)
van Manen, T. G., Prins, P. J. & Emmelkamp, P. M. (1999). A social cognitive intervention program for children with a conduct disorder, a pilot study. *Gedragstherapie, 32*(1), 33-55.	Not an RCT or quasi study
Van Ryzin, M. J. & Leve, L. D. (2012). Affiliation with delinquent peers as a mediator of the effects of multidimensional treatment foster care for delinquent girls. *Journal of Consulting and Clinical Psychology, 80*(4), 588-596.	Wrong outcomes
Van Ryzin, Mark J. & Dishion, Thomas J. (2012). The impact of a family-centered intervention on the ecology of adolescent antisocial behavior: Modeling developmental sequelae and trajectories during adolescence. *Development and Psychopathology, 24(*3), 1139-1155.	Wrong population
an Vugt, E. S., Dekovic, M., Prinzie, P., Stams, G. & Asscher, J (2013). Evaluation of a group-based social skills training for children with problem behavior. *Children and Youth Services Review, 35*(1), 162-167.	Children (0-11)
Van Vugt, E., Lanctot, N. & Lemieux, A. (2016). Can institutionalized adolescent females with a substantiated history of sexual abuse benefit from cognitive behavioral treatment targeting disruptive and delinquent behaviors? *Criminal Justice and Behavior, 43*(7), 937-950.	Wrong outcomes
Vanega-Romero, S., Sosa-Correa, M. & Castillo-Ayuso, R. (2018). Bullying, anger and depression in Mexican adolescents: A preliminary study of the effectiveness of an intervention. *Revista de Psicologia Clinica con Ninos y Adolescentes, 5*(2), 49-54.	Not an RCT or quasi study
Vannatta, T. L. (1991). *A study of the effects of self-instruction and alternate response training on oppositional behaviors* [Unpublished Doctoral Dissertation]. Ball State University.	Wrong outcomes

Vanzin, L., Mauri, V., Valli, A., Pozzi, M., Presti, G., Oppo, A., Ristallo, A., Molteni, M. & Nobile, M. (2019). Clinical effects of an act-group training in children and adolescents with attention-deficit/hyperactivity disorder. *Journal of Child and Family Studies*, DOI: 10.1007/s10826-019-01546-x	Children (0-11)
Velo, S., Kereszteny, A., Ferenczi-Dallos, G. & Balazs, J. (2019). Long-term effects of multimodal treatment on psychopathology and health-related quality of life of children with attention deficit hyperactivity disorder. *Frontiers in Psychology, 10,* DOI: 10.3389/fpsyg.2019.02037	Children (0-11)
Verwaaijen, A. A. & Van Acker, J. C. (1993). Family treatment for adolescents at risk of placement: II. Treatment process and outcome. *Family Therapy*, *20*(2), 103-132.	Nor an RCT or quasi study
Vila, S., Gilar-Corbi, R. & Pozo-Rico, T. (2021). Effects of student training in social skills and emotional intelligence on the behaviour and coexistence of adolescents in the 21st century. *International Journal of Environmental Research & Public Health, 18*(10), DOI: 10.3390/ijerph18105498	Wrong population
Visdomine-Lozano, J. C. (2020). Contextualist perspective in the treatment of antisocial behaviors and offending: A comparative review of FAP, ACT, DBT, and MDT. *Trauma, Violence & Abuse, 14,* DOI: 10.1177/1524838020939509	Review
Von Sydow, K., Beher, S., Schweitzer-Rothers, J. & Retzlaff, R. (2006). Systemic family therapy with children and adolescents as index patients. A meta-content analysis of 47 randomized controlled outcome studies. *Psychotherapeut*, *51*(2), 107-143.	Review
von Sydow, K., Retzlaff, R., Beher, S., Haun, M. W. & Schweitzer, J. (2013). The efficacy of systemic therapy for childhood and adolescent externalizing disorders: A systematic review of 47 RCT. *Family Process, 52*(4), 576-618.	Review
Waller, R., Gardner, F. & Hyde, L. W. (2013). What are the associations between parenting, callous-unemotional traits, and antisocial behavior in youth? A systematic review of evidence. *Clinical Psychology Review*, *33*(4), 593-608.	Review
Wang, Y., Liu, C & Wang, Y. F. (2007). Effectiveness of social skills training among children with behavior problems: A randomized controlled trial. *Journal of Peking University Health Sciences, 39*(3), 315-318.	Wrong outcomes
Waxmonsky, J. G., Waschbusch, D. A., Belin, P., Li, T., Babocsai, L., Humphery, H., Pariseau, M. E., Babinski, D. E., Hoffman, M. T., Haak, J. L., Mazzant, J. R., Fabiano, G. A., Pettit, J. W., Fallahazad, N. & Pelham, W. E. (2016). A randomized clinical trial of an integrative group therapy for children with severe mood dysregulation. *Journal of the American Academy of Child & Adolescent Psychiatry*, *55*(3), 196-207.	Children (0-11)
Weichold, K. (2004). Evaluation of anti-aggressiveness training with antisocial adolescents. *Gruppendynamik und Organisationsberatung, 35*(1), 83-104.	Wrong outcomes
Weichold, K. (2004). Evaluation of anti-aggressiveness training with antisocial adolescents. *Gruppendynamik und Organisationsberatung, 35*(1), 83-105.	Duplicate
Weinblatt, U. & Omer, H. (2008). Nonviolent resistance: A treatment for parents of children with acute behavior problems. *Journal of Marital & Family Therapy, 34*(1), 75-92.	Wrong outcomes
Weis, R., Wilson, N. L. & Whitemarsh, S. M. (2005). Evaluation of a voluntary, military-style residential treatment program for adolescents with academic and	Wrong intervention

conduct problems. *Journal of Clinical Child and Adolescent Psychology, 34*(4), 692-705.	
Weiss, B., Han, S., Harris, V., Catron, T., Ngo, V. K., Caron, A., Gallop, R. & Guth, C. (2013). An independent randomized clinical trial of multisystemic therapy with non-court-referred adolescents with serious conduct problems. *Journal of Consulting and Clinical Psychology, 81*(6), 1027-1039.	Wrong outcomes
Weiss, B., Han, S. S., Tran, N. T., Gallop, R. & Ngo, V. K. (2015). Test of "facilitation" vs. "proximal process" moderator models for the effects of Multisystemic Therapy on adolescents with severe conduct problem. *Journal of Abnormal Child Psychology*, *43*(5), 971-983.	Wrong outcomes
Weiss, M., Schmucker, M. & Losel, F. (2015). Meta-analytic review of family-based prevention programs in Germany. *Zeitschrift fur Klinische Psychologie und Psychotherapie, 44*(1), 27-44.	Review
Weisz, J. R., Doss, A. J. & Hawley, K. M. (2005). Youth psychotherapy outcome research: A review and critique of the evidence base. *Annual Review of Psychology, 56*, 337-363.	Review
Weisz, J., & Harvard University (2014). Maine implementation study – Phase III. *U.S National Library of Medicine Clinical Trials.* https://clinicaltrials.gov/ct2/show/NCT02229305	Wrong outcomes
Weisz, J. R., Bearman, S. K., Ugueto, A.M., Herren, J. A., Evans, S. C., Cheron, D. M., Alleyne, A. R., Weissman, A. S., Tweed, J., Pollack, A. A., Langer, D. A., Southam-Gerow, M. A., Wells, K. C. & Jensen-Doss, A. (2020). Testing robustness of child STEPs effects with children and adolescents: A randomized controlled effectiveness trial. *Journal of Clinical Child and Adolescent Psychology, 49*(6), 883-896.	Children (0-11)
Weisz, J. R., Chorpita, B. F., Palinkas, L. A., Schoenwald, S. K., Miranda, J., Bearman, S. K., Daleiden, E. L., Ugueto, A. M., Ho, A., Martin, J., Gray, J., Alleyne, A., Langer, D. A., Southam-Gerow, M. A. & Gibbons, R. D. (2012). Testing standard and modular designs for psychotherapy treating depression, anxiety, and conduct problems in youth: A randomized effectiveness trial. *Archives of General Psychiatry, 69*(3), 274-282.	Children (0-11)
Weitkamp, K., Daniels, J. K., Romer, G. & Wiegand-Grefe, S. (2017). Psychoanalytic psychotherapy for children and adolescents with severe externalising psychopathology: An effectiveness trial. *Zeitschrift fur Psychosomatische Medizin und Psychotherapie*, *63*(3), 251-266.	Children (0-11)
Weitkamp, K., Daniels, J. K., Romer, G. & Wiegand-Grefe, S. (2017). Psychoanalytic psychotherapy for children and adolescents with severe externalising psychopathology: An effectiveness trial. *Zeitschrift fur Psychosomatische Medizin und Psychotherapie*, *63*(3), 251-266.	Duplicate
Welch, M. G., Northrup, R. S., Welch-Horan, T. B., Ludwig, R. J., Austin, C. L. & Jacobson, J. S. (2006). Outcomes of prolonged parent-child embrace therapy among 102 children with behavioral disorders. *Complementary Therapies in Clinical Practice*, *12*(1), 3-12.	Children (0-11)
Westermark, P K., Hansson, K. & Olsson, M. (2011), Multidimensional treatment foster care (MTFC): Results from an independent replication. *Journal of Family Therapy*, *33*(1), 20-41.	Wrong outcomes

Wettach, R. & Aebi, M. (2016). Pilot study on the effectiveness of a multimodal group treatment for children with oppositional defiant disorder in clinical primary care. *Zeitschrift fur Kinder-und Jugendpsychiatrie und Psychotherapie, 44*(3), 220-230.	Children (0-11)
White, S. F. (2011). *Examining the influence of callous-unemotional traits on outcomes in an evidence-based treatment program for delinquent adolescents* [Unpublished Doctoral Dissertation]. University of New Orleans.	Not an RCT or quasi study
White, S. F. & Frick, P. J. (2011). *Examining the influence of callous-unemotional traits on outcomes in an evidence-based treatment program for delinquent adolescents* [Unpublished Doctoral Dissertation]. University of New Orleans.	Duplicate
White, S. F., Frick, P. J., Lawing, K. & Bauer, D. (2013). Callous-unemotional traits and response to Functional Family Therapy in adolescent offenders. *Behavioral Sciences & The Law, 31*(2), 271-285.	Not an RCT or quasi study
Whitfield, G. W. (1996). *An evaluation of anger control training with male adolescents in a day treatment program (boys, conduct disordered)* [Unpublished Doctoral Dissertation]. The University of Tennessee, Knoxville	Wrong outcomes
Whitfield, G. W. (1999). Validating school social work: An evaluation of a cognitive-behavioral approach to reduce school violence. *Research on Social Work Practice, 9*(4), 399-426.	Wrong outcomes
Whitmore, E. A., Mikulich, S. K., Ehlers, K. M. & Crowley, T. J. (2000). One-year outcome of adolescent females referred for conduct disorder and substance abuse/dependence. *Drug and Alcohol Dependence, 59*(2), 131-141.	Wrong outcomes
Whybra, L., Warner, G., Bjornstad, G., Hobbs, T., Brook, L., Wrigley, Z., Berry, V., Ukoumunne, O. C., Matthews, J., Taylor, R., Eames, T., Kallitsoglou, A., Blower, S. & Axford, N. (2018). The effectiveness of Chance UK's mentoring programme in improving behavioural and emotional outcomes in primary school children with behavioural difficulties: study protocol for a randomised controlled trial. *BMC Psychology, 6*(1), DOI: 10.1186/s40359-018-0220-9	Protocol
Wilhite, S. & Bullock, L. M. (2012). Effects of the WhyTry social skills program on students with emotional and/or behavioral problems in an alternative school. *Emotional & Behavioral Difficulties, 17*(2), 175-194.	Nor an RCT or quasi study
Wilkinson, S., Waller, R. & Viding, E. (2016). Practitioner review: Involving young people with callous unemotional traits in treatment - does it work? A systematic review. *Journal of Child Psychology and Psychiatry, 57*(5), 552-565.	Review
Wilmshurst, L. A. (2002). Treatment programs for youth with emotional and behavioral disorders: An outcome study of two alternate approaches. *Mental Health Services Research, 4*(2), 85-96.	Children (0-11)
Wilson, B. C. (2017). *The effectiveness of Promoting Alternative Thinking Strategies (PATHS) when used one time per week in therapeutic day treatment* [Unpublished Doctoral Dissertation]. Walden University.	Children (0-11)
Wilson, H. L. (2000). *The impact of social skills intervention upon severely emotionally disturbed/behaviorally disordered children* [Unpublished Doctoral Dissertation].	Wrong outcomes
Winkelmann, K., Stefini, A., Hartmann, M., Geiser-Elze, A., Kronmuller, A., Schenkenbach, C., Hildegard, H. & Kronmuller, K. T. (2005). Efficacy of psychodynamic short-term psychotherapy for children and adolescents with	Children (0-11)

behavioral disorders. *Praxis der Kinderpsychologie und Kinderpsychiatrie, 54*(7), 598-614.	
Winkelmann, K., Stefini, A., Hartmann, M., Geiser-Elze, A., Kronmuller, A., Schenkenbach, C., Hildegard, H. & Kronmuller, K. T. (2005). Efficacy of psychodynamic short-term psychotherapy for children and adolescents with behavioral disorders. *Praxis der Kinderpsychologie und Kinderpsychiatrie, 54*(7), 598-614.	Duplicate
Wolff, J. C., Greene, R. W. & Ollendick, T. H. (2008). Differential responses of children with varying degrees of reactive and proactive aggression to two forms of psychosocial treatment. *Child & Family Behavior Therapy, 30*(1), 37-50.	Children (0-11)
Wolff, J. C., Garcia, A., Kelly, L. M., Frazier, E. A., Jones, R. N. & Spirito, A. (2020). Feasibility of decision rule-based treatment of comorbid youth: A pilot randomized control trial. *Behaviour Research and Therapy, 131,* DOI: 10.1016/j.brat.2020.103625	Children (0-11)
Wong, D. F. K., Ip, P. S. Y., Chan, M. H., & Zhuang, X. Y. (2018). A preliminary study on cognitive-behavior interventions for Hong Kong Chinese delinquent youth. *Research on Social Work Practice, 28*(7), 838-847.	Wrong outcomes
Wood, A., Harrington, R. & Moore, A. (1996). Controlled trial of a brief cognitive-behavioural intervention in adolescent patients with depressive disorders. *Journal of Child Psychology and Psychiatry, 37*(6), 737-746.	Wrong outcomes
Wood, A., Trainor, G., Rothwell, J., Moore, A. & Harrington, R. (2001). Randomized trial of group therapy for repeated deliberate self-harm in adolescents. *Journal of the American Academy of Child and Adolescent Psychiatry, 40*(11), 1246-1253.	Wrong outcomes
Woolfenden, S. R., Williams, K. & Peat, J. (2001). Family and parenting interventions in children and adolescents with conduct disorder and delinquency aged 10-17. *Cochrane Database of Systematic Reviews,* Issue 2. Art. No. CD003015. DOI: 10.1002/14651858.CD003015	Review
Woolfenden, S. R., Williams, K. & Peat, J. (2002). Family and parenting interventions for conduct disorder and delinquency: A meta-analysis of randomised controlled trials. *Archives of Disease in Childhood, 86*(4), 251-256.	Review
Wright, R., John, L., Livingstone, A. M., Shepherd, N. & Duku, E. (2007). Effects of school-based interventions on secondary school students with high and low risks for antisocial behaviour. *Canadian Journal of School Psychology, 22*(1), 32-49.	Wrong population
Wynne, C., Doyle, C., Kenny, R., Brosnan, E. & Sharry, J. (2016). A first-level evaluation of a family intervention for adolescent social, emotional and behavioural difficulties in Child and Adolescent Mental Health Services. *Journal of Child &Adolescent Mental Health, 28*(1), 33-46.	Wrong outcomes
Yeheskel, A., Jekielek, A. & Sandor, P. (2020). Taking up residence: A review of outcome studies examining residential treatment for youth with serious emotional and behavioural disorders. *Children and Youth Services Review, 111,* DOI: 10.1016/j.childyouth.2020.104842	Review
Zazzali, J. L., Sherbourne, C., Hoagwood, K. E., Greene, D., Bigley, M. F. & Sexton, T. L. (2008). The adoption and implementation of an evidence based practice in child and family mental health services organizations: A pilot study of functional family therapy in New York State. *Administration and Policy in Mental Health and Mental Health Services Research, 35*(1-2), 38-49.	Not an RCT or quasi study

Zeanah, C. H., Humphreys, K., McGoron, L. K., Fox, N. A., Nelson, C. A. (2016). High-quality foster care mitigates callous-unemotional traits following early deprivation in boys: a randomized controlled trial. *Journal of the American Academy of Child and Adolescent Psychiatry, 55*(10), DOI: 10.1016/j.jaac.2016.09.485	Wrong intervention
Zeanah, C. H., Humphreys, K., McGoron, L. K., Fox, N. A., Nelson, C. A. (2016). High-quality foster care mitigates callous-unemotional traits following early deprivation in boys: a randomized controlled trial. *Journal of the American Academy of Child and Adolescent Psychiatry, 55*(10), DOI: 10.1016/j.jaac.2016.09.485	Duplicate
