# Supplementary material for: Identification of treatment elements for adolescents with callous unemotional traits: a systematic narrative review
Source: Child Adolesc Psychiatry Ment Health. 2024 Sep 3;18:110. doi: 10.1186/s13034-024-00792-2 (PMC11373131; doi:10.1186/s13034-024-00792-2)
Supplement: Supplementary file 5 — Supplementary Material 5 [file 13034_2024_792_MOESM5_ESM.pdf]

Title: Identification of Treatment Elements for Adolescents with Callous Unemotional Traits: A Systematic Narrative Review
Journal: Child and Adolescent Psychiatry and Mental Health
Authors: Pamela M. Waaler, Josefine Bergseth, Linda Vaskinn, Kristin Espenes, Thale Holtan, John Kjøbli, and Gunnar Bjørnebekk
Correspondence author: Pamela M. Waaler, Ph.D. candidate Department of Special Needs Education, University of Oslo; E-mail: p.m.waaler@isp.uio.no

Supplementary Material E

Electronic Search Strategy June 2021 & February 2023

Common callous unemotional (CU) trait elements: Systematic Review
Search strategies

Date of searches: 28th-30th of June 2021
Date of updated searches: 14th of February 2023 and 28th of March (Cochrane Central)
Total number of hits from all databases: 25790
Total number of hits after removing duplicates: 7033
Total number of hits in grey literature and trials databases: 2167
Search strategies developed by: Brynhildur Axelsdottir and Sølvi Biedilæ, information specialists.
biblioteket@r-bup.no

Databases	Number of hits primary search 2021	Number of hits updated search 2023
Medline	3595	297
PsycINFO	8749	521
Embase	2492	No access to Embase in 2023
Cochrane Central	1694	196
Cinahl	1741	186
ERIC	559	18
Sociological Abstracts	440	241
Social Care Online	89	5
Web of Science	4364	603
OpenGrey	110	Not available for search

ClinicalTrials.gov	1728	321
WHO International Clinical Trials Registry Platform	8	0

Ovid MEDLINE(R) and Epub Ahead of Print, In-Process, In-Data-Review & Other Non-Indexed Citations, Daily and Versions(R) <1946 to June 17, 2021>

#	Searches
1	Conduct Disorder/ or Child Behavior Disorders/ or "Attention Deficit and Disruptive Behavior Disorders"/ or Narcissism/
2	((callous* adj3 (unemotion* or trait*)) or ((conduct or opposition* or defian* or devian*) adj3 disorder*) or ((disrupt* or impulsiv*) adj3 (behavi* or disorder*)) or psychopath or psychopathy or psychopathic or sociopath* or narcissis* or antisocial* or anti-social* or dissocial* or unempath* or fearless*).tw.
3	((norm* adj break* adj behavi*) or (severe* adj (violen* or aggress*)) or (child* adj behavi* adj disorder*)).tw.
4	or/1-3
5	exp Adolescent/
6	(adoles* or youth* or youngster* or teen* or preteen* or minors* or juvenil* or school*).ti,ab,hw,kf.
7	(adoles* or youth* or juvenil*).jw.
8	or/5-7
9	exp Therapeutics/ or exp Psychotherapy/ or exp Pharmacologic Actions/
10	(intervention* or therap* or psychotherap* or treatment* or training* or approach* or technique* or program* or drug* or pharma*).tw.
11	or/9-10
12	randomized controlled trial.pt.

13	controlled clinical trial.pt.
14	randomized.ab.
15	placebo.ab.
16	drug therapy.fs.
17	randomly.ab.
18	trial.ab.
19	groups.ab.
20	or/12-19
21	exp animals/ not humans.sh.
22	20 not 21
23	4 and 8 and 11 and 22
24	limit 23 to yr="1990 -Current"

APA PsycInfo <1987 to June Week 2 2021>

#	Searches
1	callous-unemotional traits/ or behavior disorders/ or disruptive behavior disorders/ or conduct disorder/ or oppositional defiant disorder/ or exp impulse control disorders/ or narcissism/ or narcissistic personality disorder/
2	((callous* adj3 (unemotion* or trait*)) or ((conduct or opposition* or defian* or devian*) adj3 disorder*) or ((disrupt* or impulsiv*) adj3 (behavi* or disorder*)) or psychopath or psychopathy or psychopathic or sociopath* or narcissis* or antisocial* or anti-social* or dissocial* or unempath* or fearless*).ti,ab,id.

3	((norm* adj break* adj behavi*) or (severe* adj (violen* or aggress*)) or (child* adj behavi* adj disorder*)).ti,ab,id.
4	or/1-3
5	adolescence 13 17 yrs.ag.
6	(adoles* or youth* or youngster* or teen* or preteen* or minors* or juvenil* or school*).ti,ab,id,hw.
7	(adoles* or youth* or juvenil*).jw.
8	or/5-7
9	exp "Intervention"/ or exp treatment/ or psychosocial rehabilitation/ or psychosocial readjustment/ or exp drug therapy/
10	(intervention* or therap* or psychotherap* or treatment* or training* or approach* or technique* or program* or drug* or pharma*).ti,ab,id.
11	or/9-10
12	exp treatment/
13	(control* or random* or placebo* or double-blind*).ti,ab.
14	or/12-13
15	4 and 8 and 11 and 14
16	limit 15 to yr="1990 -Current"

Embase <1988 to 2021 Week 24>

#	Searches
1	conduct disorder/ or behavior disorder/ or oppositional defiant disorder/ or disruptive behavior/ or impulse control disorder/ or narcissism/
2	((callous* adj3 (unemotion* or trait*)) or ((conduct or opposition* or defian* or devian*) adj3 disorder*) or ((disrupt* or impulsiv*) adj3 (behavi* or disorder*)) or psychopath or

	psychopathy or psychopathic or sociopath* or narcissis* or antisocial* or anti-social* or dissocial* or unempath* or fearless*).ti,ab,kw.
3	((norm* adj break* adj behavi*) or (severe* adj (violen* or aggress*)) or (child* adj behavi* adj disorder*)).ti,ab,kw.
4	or/1-3
5	exp adolescent/ or exp adolescence/
6	(adoles* or youth* or youngster* or teen* or preteen* or minors* or juvenil* or school*).ti,ab,kw,hw.
7	(adoles* or youth* or juvenil*).jx.
8	or/5-7
9	exp therapy/ or exp psychotherapy/ or psychosocial care/ or exp drug therapy/
10	(intervention* or therap* or psychotherap* or treatment* or training* or approach* or technique* or program* or drug* or pharma*).ti,ab,kw.
11	or/9-10
12	randomized controlled trial/ or crossover procedure/ or double blind procedure/ or single blind procedure/
13	(crossover* or cross over* or placebo* or allocat* or random*).ti,ab.
14	(doubl* adj blind*).ti,ab.
15	trial*.ti.
16	or/12-15
17	4 and 8 and 11 and 16
18	limit 17 to yr="1990 -Current"

Cochrane Central Register of Controlled Trials

ID Search Hits

#1 MeSH descriptor: [Conduct Disorder] this term only

#2 MeSH descriptor: [Child Behavior Disorders] this term only

#3 ["Attention Deficit and Disruptive Behavior Disorders"] this term only

#4 MeSH descriptor: [Narcissism] this term only

#5 ((callous* NEAR/3 (unemotion* or trait*)) or ((conduct or opposition* or defian* or devian*) NEAR/3 disorder*) or ((disrupt* or impulsiv*) NEAR/3 (behavi* or disorder*)) or psychopath or psychopathy or psychopathic or sociopath* or narcissis* or antisocial* or anti-social* or dissocial* or unempath* or fearless*):ti,ab,kw

#6 ((norm* NEAR/1 break* NEAR/1 behavi*) or (severe* NEAR/1 (violen* or aggress*)) or (child* adj behavi* NEAR/ disorder*)):ti,ab,kw

#7 #1 or #2 or #3 or #4 or #5 or #6

#8 MeSH descriptor: [Adolescent] explode all trees

#9 (adoles* or youth* or youngster* or teen* or preteen* or minors* or juvenil* or school*):ti,ab,kw

#10 #8 or #9

#11 MeSH descriptor: [Therapeutics] explode all trees

#12 MeSH descriptor: [Psychotherapy] explode all trees

#13 MeSH descriptor: [Pharmacologic Actions] explode all trees

#14 (intervention* or therap* or psychotherap* or treatment* or training* or approach* or technique* or program* or drug* or pharma*):ti,ab,kw

#15 #11 or #12 or #13 or #14

#16 #7 and #10 and #15 with Publication Year from 1990 to 2021, in Trials

CINAHL (Cumulative Index to Nursing and Allied Health Literature) (EBSCO)

#	Query	Limiters/Expanders
S1	(MH "Child Behavior Disorders+")	

S2	(MH "Disruptive Behavior")
S3	(MH "Impulse Control Disorders+")
S4	(MH "Narcissism")
S5	TI (((callous* N3 (unemotion* or trait*)) or ((conduct or opposition* or defian* or devian*) N3 disorder*) or ((disrupt* or impulsiv*) N3 (behavi* or disorder*)) or psychopath or psychopathy or psychopathic or sociopath* or narcissis* or antisocial* or anti-social* or dissocial* or unempath* or fearless*)) OR AB (((callous* N3 (unemotion* or trait*)) or ((conduct or opposition* or defian* or devian*) N3 disorder*) or ((disrupt* or impulsiv*) N3 (behavi* or disorder*)) or psychopath or psychopathy or psychopathic or sociopath* or narcissis* or antisocial* or anti-social* or dissocial* or unempath* or fearless*))
S6	TI (((norm* N1 break* N1 behavi*) or (severe* N1 (violen* or aggress*)) or (child* N1 behavi* N1 disorder*))) OR AB (((norm* N1 break* N1 behavi*) or (severe* N1 (violen* or aggress*)) or (child* N1 behavi* N1 disorder*)))
S7	S1 OR S2 OR S3 OR S4 OR S5 OR S6
S8	(MH "Adolescence+") OR (MH "Minors (Legal)")
S9	TI ((adoles* or youth* or youngster* or teen* or preteen* or minors* or juvenil* or school*)) OR AB ((adoles* or youth* or youngster* or teen* or preteen* or minors* or juvenil* or school*))
S10	S8 OR S9
S11	(MH "Therapeutics+")
S12	(MH "Psychotherapy+")
S13	TI ((intervention* or therap* or psychotherap* or treatment* or training* or approach* or technique* or program* or drug* or pharma*)) OR AB ((intervention* or therap* or psychotherap* or treatment* or training* or approach* or technique* or program* or drug* or pharma*))

S14	S11 OR S12 OR S13	
S15	(MH "Double-Blind Studies") OR (MH "Randomized Controlled Trials+") OR (MH "Single-Blind Studies")	
S16	(MH "Random Assignment") OR (MH "Simple Random Sample") OR (MH "Stratified Random Sample") OR (MH "Systematic Random Sample")	
S17	(MH "Pretest-Posttest Design+") OR (MH "Pretest-Posttest Control Group Design")	
S18	TI ((control* or random* or placebo* or double-blind*)) OR AB ((control* or random* or placebo* or double-blind*))	
S19	S15 OR S16 OR S17 or S18	
S21	S7 AND S10 AND S14 AND S19	Limiters - Published Date: 19900101-20211231

ERIC (Education Resources Information Center) (EBSCO)

#	Query	Limiters/Expanders
S1	DE "Behavior Disorders"	
S2	DE "Antisocial Behavior" OR DE "Self Destructive Behavior"	
S3	TI (((callous* NEAR/3 (unemotion* or trait*)) or ((conduct or opposition* or defian* or devian*) NEAR/3 disorder*) or ((disrupt* or impulsiv*) NEAR/3 (behavi* or disorder*)) or psychopath or psychopathy or psychopathic or sociopath* or narcissis* or antisocial* or anti-social* or dissocial* or unempath* or fearless*)) OR AB (((callous* NEAR/3 (unemotion* or trait*)) or ((conduct or opposition* or defian* or devian*) adj3 disorder*) or ((disrupt* or impulsiv*) NEAR/3 (behavi* or disorder*)) or psychopath or	

psychopathy or psychopathic or sociopath* or narcissis* or antisocial* or anti-social* or dissocial* or unempath* or fearless*))

S4	TI (((norm* NEAR/1 break* NEAR/1 behavi*) or (severe* NEAR/1 (violen* or aggress*)) or (child* NEAR/1 behavi* adj disorder*))) OR AB (((norm* adj break* NEAR/1 behavi*) or (severe* NEAR/1 (violen* or aggress*)) or (child* NEAR/1 behavi* NEAR/1 disorder*)))
S5	S1 OR S2 OR S3 OR S4
S6	DE "Late Adolescents" OR DE "Adolescents"
S7	TI ((adoles* or youth* or youngster* or teen* or preteen* or minors* or juvenil* or school*)) OR AB ((adoles* or youth* or youngster* or teen* or preteen* or minors* or juvenil* or school*))
S8	SO (adoles* or youth* or juvenil*)
S9	S6 OR S7 OR S8
S10	DE "Psychotherapy" OR DE "Milieu Therapy" OR DE "Relaxation Training"
S11	DE "Intervention"
S12	DE "Therapy" OR DE "Drug Therapy"
S13	TI ((intervention* or therap* or psychotherap* or treatment* or training* or approach* or technique* or program* or drug* or pharma*)) OR AB ((intervention* or therap* or psychotherap* or treatment* or training* or approach* or technique* or program* or drug* or pharma*))
S14	S10 OR S11 OR S12 OR S13
S15	DE "Randomized Controlled Trials" OR DE "Control Groups" OR DE "Experimental Groups"

S16	TI ((control* or random* or placebo* or double-blind*)) OR AB ((control* or random* or placebo* or double-blind*))	
S17	S15 OR S16	
S18	S5 AND S9 AND S14 AND S17	Limiters - Date Published: 19900101-20211231

Web of Science (Clarivate Analytics)

# 1 TS=((callous* NEAR/3 (unemotion* or trait*)) or ((conduct or opposition* or defian* or devian*) NEAR/3 disorder*) or ((disrupt* or impulsiv*) NEAR/3 (behavi* or disorder*)) or psychopath or psychopathy or psychopathic or sociopath* or narcissis* or antisocial* or anti-social* or dissocial* or unempath* or fearless*)
Indexes=SSCI, A&HCI Timespan=1990-2021

# 2 TS=((norm* NEAR break* NEAR behavi*) or (severe* NEAR (violen* or aggress*)) or (child* NEAR behavi* NEAR disorder*))
Indexes=SSCI, A&HCI Timespan=1990-2021

# 3 #2 OR #1
Indexes=SSCI, A&HCI Timespan=1990-2021

# 4 TS=(adoles* or youth* or youngster* or teen* or preteen* or minors* or juvenil* or school*)
Indexes=SSCI, A&HCI Timespan=1990-2021

# 5 TS=(intervention* or therap* or psychotherap* or treatment* or training* or approach* or technique* or program* or drug* or pharma*)
Indexes=SSCI, A&HCI Timespan=1990-2021

# 6 TS=(control* or random* or placebo* or double-blind*)
Indexes=SSCI, A&HCI Timespan=1990-2021

# 7 #6 AND #5 AND #4 AND #3
Indexes=SSCI, A&HCI Timespan=1990-2021

Sociological Abstracts (ProQuest)

MAINSUBJECT.EXACT.EXPLODE("Deviant Behavior") OR MAINSUBJECT.EXACT("narcissism") OR noft(("callous unemotional" OR "callous trait" OR "unemotional traits" OR "conduct disorder" OR "conduct disorders" OR "oppositional disorder" OR "oppositional disorders " OR "defiant disorder" OR "defiant disorders" OR "deviant disorder" OR "deviant disorders" OR "disrupt disorder" OR "disrupt disorders" OR "disrupt behavior" OR "disrupt behaviour" OR "impulsive behavior" OR "impulsive behaviour" OR psychopath OR psychopathy OR psychopathic OR sociopath OR narcissist OR narcissism OR antisocial OR "anti-social" OR dissocial OR unempathetic OR fearless OR "norm breaking behavior" OR "norm breaking behaviour" OR "severe violent" OR "severe violence" OR "severe aggressive" OR "severe aggression" OR "child behavior disorder" OR "child behaviour disorder" OR "child behavior disorders" OR "child behaviour disorders")) AND mainsubject.Exact("adolescents") OR noft((adolescent OR adolescence OR youth OR youngster OR teen OR teens OR preteen OR preteens OR minor OR minors OR juvenile OR school)) AND MAINSUBJECT.EXACT("intervention") OR MAINSUBJECT.EXACT("treatment") OR MAINSUBJECT.EXACT("behavior modification") OR noft((intervention OR interventions OR therapy OR therapies OR psychotherapy OR treatment OR treatments OR training OR approach OR technique OR techniques OR program OR programs OR drug OR drugs OR pharma)) AND MAINSUBJECT.EXACT("treatment outcomes") OR noft((control OR controlled OR random OR randomly OR randomized OR randomized OR placebo OR "double blind" OR "double-blind" OR "double-blinded" OR "double blinded"))

Social Care Online
https://www.scie-socialcareonline.org.uk/

[- SubjectTerms:'"conduct disorders"' including this term only
- OR AllFields:'"callous unemotion*"'
- OR AllFields:'"conduct disorder*"'
- OR AllFields:'disrupt*'
- OR AllFields:'impulsiv*'
- OR AllFields:'psychopath'
- OR AllFields:'psychopathy'
- OR AllFields:'sociopath*'
- OR AllFields:'narcissis*'
- OR AllFields:'antisocial*'
- OR AllFields:'anti-social*'
- OR AllFields:'dissocial*'

- OR AllFields:'unempath*'
- OR AllFields:'fearless*']

AND

[- SubjectTerms:'"young people"' including this term only
- OR SubjectTerms:'"adolescence"' including narrower terms
- OR AllFields:'adoles*'
- OR AllFields:'youth*'
- OR AllFields:'youngster*'
- OR AllFields:'teen*'
- OR AllFields:'preteen*'
- OR AllFields:'minors*'
- OR AllFields:'juvenil*'
- OR AllFields:'school*']

AND

[- SubjectTerms:'"intervention"' including this term only
- OR SubjectTerms:'"therapy and treatment"' including narrower terms
- OR SubjectTerms:'"psychotherapy"' including this term only
- OR AllFields:'intervention*'
- OR AllFields:'therap*'
- OR AllFields:'psychotherap*'
- OR AllFields:'treatment*'
- OR AllFields:'training*'
- OR AllFields:'approach*'
- OR AllFields:'technique*'
- OR AllFields:'program*'
- OR AllFields:'drug*'
- OR AllFields:'pharma*']

AND

[- SubjectTerms:'"randomised controlled trials"' including this term only
- OR AllFields:'control*'
- OR AllFields:'random*'
- OR AllFields:'placebo*'
- OR AllFields:'double-blind*']

Grey literature, ongoing and unpublished trials:

ClinicalTrials.gov:
2 Studies found for: Interventional Studies | callous* unemotional* | Child
Applied Filters: Interventional Child (birth–17)
https://clinicaltrials.gov/ct2/results?cond=callous*+unemotional*&age_v=&age=0&gndr=&type=Intr&rslt=&Search=Apply

3 Studies found for: callous unemotional trait | Child
Applied Filters: Child (birth–17)
https://clinicaltrials.gov/ct2/results?cond=callous+unemotional+trait&age_v=&age=0&gndr=&type=&rslt=&Search=Apply

1723 Studies found for: Interventional Studies | Conduct Disorder | Child
Also searched for Behavior, Behavioral, and Diseases. See Search Details
Applied Filters: Interventional Child (birth–17)
https://clinicaltrials.gov/ct2/results?cond=Conduct+Disorder&age_v=&age=0&gndr=&type=Intr&rslt=&Search=Apply

WHO International Clinical Trials Registry Platform:
8 records for 8 trials found for: callous* unemotional*

Main ID	Public Title	Date of Registration	Results available
NCT04324099	Investigating the Specificity of Neural Correlates for Emotion Processing Deficits in Conduct Disorder and Autism Spectrum Disorders	2020-03-24	
NCT04159168	Facial Affect Sensitivity Training for Young Children With Callous-unemotional Traits	2019-10-28	
ACTRN12619000967189	Improving mental health outcomes through parent engagement in school-based early intervention	2019-07-09	
ACTRN12619000239167	Testing an Early Intervention Programme for Very Young Children with Emerging Disruptive Behaviour	2019-02-18	
ACTRN12616000280404	Parent-Child Interaction Therapy for Treating Preschool Conduct Problems	2016-03-03	
ACTRN12612000155897	Emotional Engagement Treatment for Disruptive Children with Callous-Unemotional Traits	2012-02-03	

**ISRCTN62822052**	**An innovative early intervention for antisocial children with callous-unemotional traits**	**2011-08-22**	
**NCT01362946**	**Behavioral Treatment for Children With Conduct Problems and Callous-Unemotional Traits**	**2011-01-07**	**Yes**

Opengrey:

callous* unemotional*
No hits

conduct* disorder* AND child*
82 hits
http://www.opengrey.eu/search/request?q=conduct*+disorder*+AND+child*

conduct* disorder* AND adolescen*
28 hits
http://www.opengrey.eu/search/request?q=conduct*+disorder*+AND+adolescen*

Search strategies of the updated searches in 2023

Ovid MEDLINE(R) ALL 1946 to February 13, 2023

#	Searches
1	Conduct Disorder/ or Child Behavior Disorders/ or "Attention Deficit and Disruptive Behavior Disorders"/ or Narcissism/
2	((callous* adj3 (unemotion* or trait*)) or ((conduct or opposition* or defian* or devian*) adj3 disorder*) or ((disrupt* or impulsiv*) adj3 (behavi* or disorder*)) or psychopath or psychopathy or psychopathic or sociopath* or narcissis* or antisocial* or anti-social* or dissocial* or unempath* or fearless*).tw.
3	((norm* adj break* adj behavi*) or (severe* adj (violen* or aggress*)) or (child* adj behavi* adj disorder*)).tw.
4	or/1-3
5	exp Adolescent/
6	(adoles* or youth* or youngster* or teen* or preteen* or minors* or juvenil* or school*).ti,ab,hw,kf.
7	(adoles* or youth* or juvenil*).jw.
8	or/5-7
9	exp Therapeutics/ or exp Psychotherapy/ or exp Pharmacologic Actions/
10	(intervention* or therap* or psychotherap* or treatment* or training* or approach* or technique* or program* or drug* or pharma*).tw.
11	or/9-10
12	randomized controlled trial.pt.
13	controlled clinical trial.pt.
14	randomized.ab.
15	placebo.ab.

16 drug therapy.fs.

17 randomly.ab.

18 trial.ab.

19 groups.ab.

20 or/12-19

21 exp animals/ not humans.sh.

22 20 not 21

23 4 and 8 and 11 and 22

24 limit 23 to yr="1990 -Current"

25 (202106* or 202107* or 202108* or 202109* or 202110* or 202111* or 202112* or 2022* or 202301* or 202302*).ez,ed,up.

26 24 AND 25

APA PsycInfo <1806 to February Week 1 2023>

#	Searches
1	callous-unemotional traits/ or behavior disorders/ or disruptive behavior disorders/ or conduct disorder/ or oppositional defiant disorder/ or exp impulse control disorders/ or narcissism/ or narcissistic personality disorder/
2	((callous* adj3 (unemotion* or trait*)) or ((conduct or opposition* or defian* or devian*) adj3 disorder*) or ((disrupt* or impulsiv*) adj3 (behavi* or disorder*)) or psychopath or psychopathy or psychopathic or sociopath* or narcissis* or antisocial* or anti-social* or dissocial* or unempath* or fearless*).ti,ab,id.
3	((norm* adj break* adj behavi*) or (severe* adj (violen* or aggress*)) or (child* adj behavi* adj disorder*)).ti,ab,id.
4	or/1-3

5 adolescence 13 17 yrs.ag.

6 (adoles* or youth* or youngster* or teen* or preteen* or minors* or juvenil* or school*).ti,ab,id,hw.

7 (adoles* or youth* or juvenil*).jw.

8 or/5-7

9 exp "Intervention"/ or exp treatment/ or psychosocial rehabilitation/ or psychosocial readjustment/ or exp drug therapy/

10 (intervention* or therap* or psychotherap* or treatment* or training* or approach* or technique* or program* or drug* or pharma*).ti,ab,id.

11 or/9-10

12 exp treatment/

13 (control* or random* or placebo* or double-blind*).ti,ab.

14 or/12-13

15 4 and 8 and 11 and 14

16 limit 15 to yr="1990 -Current"

17 (202106* or 202107* or 202108* or 202109* or 202110* or 202111* or 202112* or 2022* or 202301* or 202302*).up.

18 16 AND 17

Web of Science (Clarivate Analytics)

# 1 TS=((callous* NEAR/3 (unemotion* or trait*)) or ((conduct or opposition* or defian* or devian*) NEAR/3 disorder*) or ((disrupt* or impulsiv*) NEAR/3 (behavi* or disorder*)) or psychopath or psychopathy or psychopathic or sociopath* or narcissis* or antisocial* or anti-social* or dissocial* or unempath* or fearless*)
Timespan=2021-05-01 to 2023-02-14

# 2 TS=((norm* NEAR break* NEAR behavi*) or (severe* NEAR (violen* or aggress*)) or (child* NEAR behavi* NEAR disorder*))
Timespan=2021-05-01 to 2023-02-14

# 3 #2 OR #1
Timespan=2021-05-01 to 2023-02-14

# 4 TS=(adoles* or youth* or youngster* or teen* or preteen* or minors* or juvenil* or school*)
Timespan=2021-05-01 to 2023-02-14

# 5 TS=(intervention* or therap* or psychotherap* or treatment* or training* or approach* or technique* or program* or drug* or pharma*)
Timespan=2021-05-01 to 2023-02-14

# 6 TS=(control* or random* or placebo* or double-blind*)
Timespan=2021-05-01 to 2023-02-14

# 7 #6 AND #5 AND #4 AND #3
Timespan=2021-05-01 to 2023-02-14

CINAHL (Cumulative Index to Nursing and Allied Health Literature) (EBSCO)

#	Query	Limiters/Expanders
S1	(MH "Child Behavior Disorders+")	
S2	(MH "Disruptive Behavior")	
S3	(MH "Impulse Control Disorders+")	
S4	(MH "Narcissism")	
S5	TI (((callous* N3 (unemotion* or trait*)) or ((conduct or opposition* or defian* or devian*) N3 disorder*) or ((disrupt* or impulsiv*) N3 (behavi* or disorder*)) or psychopath or psychopathy or psychopathic or sociopath* or narcissis* or antisocial* or anti-social* or dissocial* or unempath* or fearless*)) OR AB (((callous* N3 (unemotion* or trait*)) or ((conduct or opposition* or defian* or devian*) N3 disorder*) or ((disrupt* or impulsiv*) N3 (behavi* or disorder*)) or psychopath or psychopathy or psychopathic or	

	sociopath* or narcissis* or antisocial* or anti-social* or dissocial* or unempath* or fearless*))
S6	TI (((norm* N1 break* N1 behavi*) or (severe* N1 (violen* or aggress*)) or (child* N1 behavi* N1 disorder*))) OR AB (((norm* N1 break* N1 behavi*) or (severe* N1 (violen* or aggress*)) or (child* N1 behavi* N1 disorder*)))
S7	S1 OR S2 OR S3 OR S4 OR S5 OR S6
S8	(MH "Adolescence+") OR (MH "Minors (Legal)")
S9	TI ((adoles* or youth* or youngster* or teen* or preteen* or minors* or juvenil* or school*)) OR AB ((adoles* or youth* or youngster* or teen* or preteen* or minors* or juvenil* or school*))
S10	S8 OR S9
S11	(MH "Therapeutics+")
S12	(MH "Psychotherapy+")
S13	TI ((intervention* or therap* or psychotherap* or treatment* or training* or approach* or technique* or program* or drug* or pharma*)) OR AB ((intervention* or therap* or psychotherap* or treatment* or training* or approach* or technique* or program* or drug* or pharma*))
S14	S11 OR S12 OR S13
S15	(MH "Double-Blind Studies") OR (MH "Randomized Controlled Trials+") OR (MH "Single-Blind Studies")
S16	(MH "Random Assignment") OR (MH "Simple Random Sample") OR (MH "Stratified Random Sample") OR (MH "Systematic Random Sample")
S17	(MH "Pretest-Posttest Design+") OR (MH "Pretest-Posttest Control Group Design")

S18	TI ((control* or random* or placebo* or double-blind*)) OR AB ((control* or random* or placebo* or double-blind*))	
S19	S15 OR S16 OR S17 or S18	
S21	S7 AND S10 AND S14 AND S19	Limiters - Published Date: 20210501-20230231

ERIC (Education Resources Information Center) (EBSCO)

#	Query	Limiters/Expanders
S1	DE "Behavior Disorders"	
S2	DE "Antisocial Behavior" OR DE "Self Destructive Behavior"	
S3	TI (((callous* NEAR/3 (unemotion* or trait*)) or ((conduct or opposition* or defian* or devian*) NEAR/3 disorder*) or ((disrupt* or impulsiv*) NEAR/3 (behavi* or disorder*)) or psychopath or psychopathy or psychopathic or sociopath* or narcissis* or antisocial* or anti-social* or dissocial* or unempath* or fearless*)) OR AB (((callous* NEAR/3 (unemotion* or trait*)) or ((conduct or opposition* or defian* or devian*) adj3 disorder*) or ((disrupt* or impulsiv*) NEAR/3 (behavi* or disorder*)) or psychopath or psychopathy or psychopathic or sociopath* or narcissis* or antisocial* or anti-social* or dissocial* or unempath* or fearless*))	
S4	TI (((norm* NEAR/1 break* NEAR/1 behavi*) or (severe* NEAR/1 (violen* or aggress*)) or (child* NEAR/1 behavi* adj disorder*))) OR AB (((norm* adj break* NEAR/1 behavi*) or (severe* NEAR/1 (violen* or aggress*)) or (child* NEAR/1 behavi* NEAR/1 disorder*)))	
S5	S1 OR S2 OR S3 OR S4	
S6	DE "Late Adolescents" OR DE "Adolescents"	

S7	TI ((adoles* or youth* or youngster* or teen* or preteen* or minors* or juvenil* or school*)) OR AB ((adoles* or youth* or youngster* or teen* or preteen* or minors* or juvenil* or school*))	
S8	SO (adoles* or youth* or juvenil*)	
S9	S6 OR S7 OR S8	
S10	DE "Psychotherapy" OR DE "Milieu Therapy" OR DE "Relaxation Training"	
S11	DE "Intervention"	
S12	DE "Therapy" OR DE "Drug Therapy"	
S13	TI ((intervention* or therap* or psychotherap* or treatment* or training* or approach* or technique* or program* or drug* or pharma*)) OR AB ((intervention* or therap* or psychotherap* or treatment* or training* or approach* or technique* or program* or drug* or pharma*))	
S14	S10 OR S11 OR S12 OR S13	
S15	DE "Randomized Controlled Trials" OR DE "Control Groups" OR DE "Experimental Groups"	
S16	TI ((control* or random* or placebo* or double-blind*)) OR AB ((control* or random* or placebo* or double-blind*))	
S17	S15 OR S16	
S18	S5 AND S9 AND S14 AND S17	Limiters - Date Published: 20210501-20230231

Sociological Abstracts (ProQuest)

MAINSUBJECT.EXACT.EXPLODE("Deviant Behavior") OR MAINSUBJECT.EXACT("narcissism") OR noft(("callous unemotional" OR "callous trait" OR "unemotional traits" OR "conduct disorder" OR "conduct disorders" OR "oppositional disorder" OR "oppositional disorders " OR "defiant disorder" OR

"defiant disorders" OR "deviant disorder" OR "deviant disorders" OR "disrupt disorder" OR "disrupt disorders" OR "disrupt behavior" OR "disrupt behaviour" OR "impulsive behavior" OR "impulsive behaviour" OR psychopath OR psychopathy OR psychopathic OR sociopath OR narcissist OR narcissism OR antisocial OR "anti-social" OR dissocial OR unempathetic OR fearless OR "norm breaking behavior" OR "norm breaking behaviour" OR "severe violent" OR "severe violence" OR "severe aggressive" OR "severe aggression" OR "child behavior disorder" OR "child behaviour disorder" OR "child behavior disorders" OR "child behaviour disorders")) AND mainsubject.Exact("adolescents") OR noft((adolescent OR adolescence OR youth OR youngster OR teen OR teens OR preteen OR preteens OR minor OR minors OR juvenile OR school)) AND MAINSUBJECT.EXACT("intervention") OR MAINSUBJECT.EXACT("treatment") OR MAINSUBJECT.EXACT("behavior modification") OR noft((intervention OR interventions OR therapy OR therapies OR psychotherapy OR treatment OR treatments OR training OR approach OR technique OR techniques OR program OR programs OR drug OR drugs OR pharma)) AND MAINSUBJECT.EXACT("treatment outcomes") OR noft((control OR controlled OR random OR randomly OR randomized OR randomized OR placebo OR "double blind" OR "double-blind" OR "double-blinded" OR "double blinded")) Date: From 01 May 2021 to 14 February 2023

Social Care Online
https://www.scie-socialcareonline.org.uk/

[- SubjectTerms:'"conduct disorders"' including this term only
- OR AllFields:'"callous unemotion*"'
- OR AllFields:'"conduct disorder*"'
- OR AllFields:'disrupt*'
- OR AllFields:'impulsiv*'
- OR AllFields:'psychopath'
- OR AllFields:'psychopathy'
- OR AllFields:'sociopath*'
- OR AllFields:'narcissis*'
- OR AllFields:'antisocial*'
- OR AllFields:'anti-social*'
- OR AllFields:'dissocial*'
- OR AllFields:'unempath*'
- OR AllFields:'fearless*']

AND

[- SubjectTerms:'"young people"' including this term only
- OR SubjectTerms:'"adolescence"' including narrower terms
- OR AllFields:'adoles*'

- OR AllFields:'youth*'
- OR AllFields:'youngster*'
- OR AllFields:'teen*'
- OR AllFields:'preteen*'
- OR AllFields:'minors*'
- OR AllFields:'juvenil*'
- OR AllFields:'school*']

AND

[- SubjectTerms:'"intervention"' including this term only
- OR SubjectTerms:'"therapy and treatment"' including narrower terms
- OR SubjectTerms:'"psychotherapy"' including this term only
- OR AllFields:'intervention*'
- OR AllFields:'therap*'
- OR AllFields:'psychotherap*'
- OR AllFields:'treatment*'
- OR AllFields:'training*'
- OR AllFields:'approach*'
- OR AllFields:'technique*'
- OR AllFields:'program*'
- OR AllFields:'drug*'
- OR AllFields:'pharma*']

AND

[- SubjectTerms:'"randomised controlled trials"' including this term only
- OR AllFields:'control*'
- OR AllFields:'random*'
- OR AllFields:'placebo*'
- OR AllFields:'double-blind*']

Sorted: Publication year: 2021-2023

Cochrane Central Register of Controlled Trials

ID	Search	Hits
#1	MeSH descriptor: [Conduct Disorder] this term only	323
#2	MeSH descriptor: [Child Behavior Disorders] this term only	931
#3	"Attention Deficit and Disruptive Behavior Disorders"	387
#4	MeSH descriptor: [Narcissism] this term only	21
#5	((callous* NEAR/3 (unemotion* or trait*)) or ((conduct or opposition* or defian* or devian*) NEAR/3 disorder*) or ((disrupt* or impulsiv*) NEAR/3 (behavi* or disorder*)) or psychopath or	

psychopathy or psychopathic or sociopath* or narcissis* or antisocial* or anti-social* or dissocial* or unempath* or fearless*):ti,ab,kw 4088
#6 ((norm* NEAR/1 break* NEAR/1 behavi*) or (severe* NEAR/1 (violen* or aggress*)) or (child* NEAR/1 behavi*NEAR/1 disorder*)):ti,ab,kw 68
#7 #1 or #2 or #3 or #4 or #5 or #6 4875
#8 MeSH descriptor: [Adolescent] explode all trees 121545
#9 (adoles* or youth* or youngster* or teen* or preteen* or minors* or juvenil* or school*):ti,ab,kw 188455
#10 #8 or #9 188455
#11 MeSH descriptor: [Therapeutics] explode all trees 373622
#12 MeSH descriptor: [Psychotherapy] explode all trees 30898
#13 MeSH descriptor: [Pharmacologic Actions] explode all trees 279698
#14 (intervention* or therap* or psychotherap* or treatment* or training* or approach* or technique* or program* or drug* or pharma*):ti,ab,kw 1592782
#15 #11 or #12 or #13 or #14 1622724
#16 #7 and #10 and #15 with Publication Year from 2021 to 2023, in Trials 196

__Grey literature, ongoing and unpublished trials:__

ClinicalTrials.gov:
No new studies found for: Interventional Studies | callous* unemotional* | Child
Applied Filters: Interventional Child (birth–17)
https://clinicaltrials.gov/ct2/results?cond=callous*+unemotional*&age_v=&age=0&gndr=&type=Intr&rslt=&Search=Apply

No new studies found for: callous unemotional trait | Child
Applied Filters: Child (birth–17)
https://clinicaltrials.gov/ct2/results?cond=callous+unemotional+trait&age_v=&age=0&gndr=&type=&rslt=&Search=Apply

2044 (321 new studies) found for: Interventional Studies | Conduct Disorder | Child
Also searched for Behavior, Behavioral, and Diseases. See Search Details
Applied Filters: Interventional Child (birth–17)
https://clinicaltrials.gov/ct2/results?cond=Conduct+Disorder&age_v=&age=0&gndr=&type=Intr&rslt=&Search=Apply

WHO International Clinical Trials Registry Platform:
No new studies found for: callous* unemotional*

Opengrey:

Not available
