# Supplementary material for: Identification of treatment elements for adolescents with callous unemotional traits: a systematic narrative review
Source: Child Adolesc Psychiatry Ment Health. 2024 Sep 3;18:110. doi: 10.1186/s13034-024-00792-2 (PMC11373131; doi:10.1186/s13034-024-00792-2)
Supplement: Supplementary file 6 — Supplementary Material 6 [file 13034_2024_792_MOESM6_ESM.pdf]

Title: Identification of Treatment Elements for Adolescents with Callous Unemotional Traits: A Systematic Narrative Review
Journal: Child and Adolescent Psychiatry and Mental Health
Authors: Pamela M. Waaler, Josefine Bergseth, Linda Vaskinn, Kristin Espenes, Thale Holtan, John Kjøbli, and Gunnar Bjørnebekk
Correspondence author: Pamela M. Waaler, Ph.D. candidate Department of Special Needs Education, University of Oslo; E-mail: p.m.waaler@isp.uio.no

Supplementary Material F

Final Element Codebook

Variable name in SPSS	Practice elements:	A practice element is a distinct practice or action performed during an intervention (such as tutoring, positive reinforcement, or timeout)
	ORGANIZATION	
1	Set goals for treatment	E.g., review or discuss the aims of the interventions, review goal attainment or set goals for treatment
2	Review goals for treatment	E.g., expectations, nature of intervention
3	Assign tasks required to accomplish treatment goals	
4	Review progress and/or celebrate change	
5	Session review/integration of information	E.g., previous or current session

6	Prepare for termination of intervention	E.g., generalize learned skills; extend positive changes into new situations/contexts
7	Discussion of experiences during treatment/intervention/intervention element	E.g positive or negative experiences
8	Alliance with facilitator, group members, or caregivers	
71	Increase contact quality with the community	E.g., school, services, etc.
72	Identify risk/protective factors in the community	
	TRAINING IN EMOTIONAL RECOGNITION AND DIFFERENTIATION	
9	Learn to recognize basic emotions	I.e., happy, sad, anger, fear, disgust, and surprise
10	Learn to identify emotions from various modalities	I.e., facial, auditory, postural
11	Learn to identify triggers for different types of emotions	E.g., discussion of the relation between emotions and social contexts, providing real-life examples.

12	Learn to infer the emotional states of others through hypothetical situations	
13	Practice expressing/communicating emotions	
14	Learn how thoughts contribute to feelings	E.g., use feeling cards to explain the thought that would be needed to experience the listed emotion given a stated event
15	Practice emotion recognition/awareness in daily life	E.g., participants are asked to write down incidents that raises feelings of anger, sadness, joy or fear within the next two days
16	Practice to avoid assumptions about how others might feel/their intentions	E.g., "The fortune telling game", learn that people have different perceptions of similar situations
17	Learn to infer the emotional states of others through real life scenarios	
	PSYCHOEDUCATION	
18	Psychoeducation on emotion recognition	E.g., The importance of accurate emotion recognition for daily functioning, and cues (e.g., facial, auditory, postural) that youth can attend to in order to improve accuracy in emotion recognition.
19	Psychoeducation on emotion awareness	E.g., focusing on increasing awareness of emotions in self and others.

20	Psychoeducation on perspective taking	E.g., focusing on the importance and personal benefits of accurate perspective taking
24	Psychoeducation on anger	E.g., how the anger mercury can build up if we do not address our feelings
25	Psychoeducation on the connection between events, thoughts, and feelings	
26	About treatment/treatment techniques	
	INCREASE MOTIVATION	
27	Use of positive reinforcement	E.g., rewarding desirable outcomes
28	Enhancing motivation and engagement	E.g., motivation for change; motivational interviewing (adopting collaborative stance, evoking adolescents' own reasons for practicing and engaging in these skills, respecting adolescents' autonomy to make their own decisions)
	PROBLEM SOLVING SKILLS	

31	Practice problem solving skills	E.g., through role-play, discussions
70	Planning for the future	E.g., create prevention-relapse plan, who to contact in the future if there are more problems, plan for future extrafamilial stresses, etc.
	SELF-EXPLORATION/SELF-MONITORING OF THOUGHTS AND FEELINGS	
32	Objects like me exercise	I.e., members of the group were asked to tell the others how they were similar to pre-selected items (e.g., a comb, tape, a small figure)
33	Exploring the youth's perspectives and opinions	E.g., perspectives about SU behavior, emotion, empathy, and interpersonal relationships; doubt opinions that might be inconsistent with prevalence base-rates or not supported by empirical literature (e.g., beliefs that most adolescents drink and smoke marijuana)
34	Write a story or draw a picture of an event that was lifechanging	E.g., to better understand own values and how these have affected their life
35	Explore the feeling of anger	E.g., list a wide range of people and events that were likely to contribute to feelings of anger, consider how angry each situation made them feel

36	Explore/improve self-esteem	E.g., by assisting group members in recognizing what others perceive as their positive characteristics
37	Personal benefits/self-interests related to intervention elements	E.g., to capitalize on a reward-dominant orientation
	TRAINING IN PREVENTING MALADAPTIVE BEHAVIORAL RESPONSE TO EMOTIONAL DISTRESS	
39	Alternative actions to maladaptive behavior	E.g., alternatives to choose when presented with, or to avoid, triggers for maladaptive behavior
40	Anger/aggression management	E.g., modification of contextual cues of threat
42	Modify contextual cues of criminal opportunity	E.g., retraining emotional response and acceptance of responsibility for actions and reactions
68	Reduce negativitiy and blame	E.g., between family members, own actions, interrupt negative interactions between family members
69	Reduce substance use	

	PARENT SKILLS TRAINING	
43	Teach parents skills and strategies to effect change in relevant domains	E.g., address communication problems, encourage school attendance and achievement, reduce young person's association with delinquent peers
66	Increase parental supervision/monitoring	
67	Clarify and establish parental expectations	
44	Enhance interpersonal support	E.g., enhance family relationships, increase support from social networks
	COGNITIVE SKILLS	
45	Teach cognitive reframing and restructuring of cognitive distortions	E.g., self-attributions overvaluing the self at the expense of others
46	Evaluate consequences of behavior	E.g., the positive and negative consequences of utilizing physical aggression
48	Practice identifying thinking errors	E.g., "Name that thinking error"

49	Give personal examples of thinking errors	E.g., provide personal examples of thoughts that are consistent with introduced thinking errors
61	Focus on the present	
62	Practice validation	E.g., recognize feelings/situations/"truths"
63	Accepting responsibility	
64	Minimize hopelessness/increase hope	E.g., changing the meaning of family relationships to emphasize possible hopeful experiences; develop/generate themes that "explain" the family's situation (i.e., you guys really care about each other); problems can be solved; normalize
65	Change meaning	E.g., changing meaning, attributions and experiences, re-labeling; reframing; creating a narrative
	STRESS MANAGEMENT	
50	Stress inoculation training	Stress inoculation training or exposure to stress in a controlled way

	SOCIAL SKILLS TRAINING	
51	Review and discuss group format and group rules	E.g., collaborate to decide rules that should apply to the group process
52	Ice-breaking exercise	E.g., group members share answers on questions with the group ("What is the best movie you have ever seen?")
53	Encourage group cohesion	
54	Practice interpersonal/communication skills	
55	Resisting peer pressure	
56	Increase/decrease contact with peers	e.g., increase contact with positive peers/decrease contact with negative peers
57	Enhance involvement in prosocial activities	

58	Identify/describe relational functions	E.g., risk/protective factors in the family, dynamics, resources, limitations, find familial patterns
59	Conflict management and negotiation skills	E.g., between family members or peers
60	Improve family relationships	E.g. styrkebaserte relasjonelle utsagn
Variable name in SPSS	Characteristics and process elements	Describes or categorize the how, when, where, why, for whom, and by whom the practice elements were delivered
	TIME OF OUTCOME MEASURE	
Time_first_PM_weeks	Time of first post-measure	Note in weeks after intervention end, first measure after intervention
Measure_short_post	Short term post-measure	Within two months after intervention
Measure_mid_post	Mid-term post measure	Two to 12 months after intervention
Measure_long_post	Long term post-measure	More than 12 months after intervention
Time_last_FU_months	Time of last follow-up	Note in months after intervention end, last follow-up

	DELIVERY METHOD	
Formal therapy	E.g., cognitive therapy, structural family therapy, strategic family therapy	
Location of treatment	Home (family based), school, neighborhood (community-based)	
Psychoeducation	Intervention included psychoeducation/dyadic learn & teaching	
Role-play	Role-play youth, parent, or intervention deliverer	
Rotate role-play roles		
Homework	Homework	
Homework_reviewed	Group discussion/review about homework	
Group_discussion	Discussions in a group setting	
Modeling	Modeling by intervention deliverer, peers, parent	
Practice exercises		
Important others	Parents/primary caretaker receive intervention elements	
Non-judgemental approach		
	MATERIALS AND GAMES	
Games	Board or computer game	

Clips	Visual or auditory clips	
Static stimuli	E.g., photos of facial expressions, auditory clips, photos of static body postures	
Anger Thermometer		E.g., Thermometers that range from ten degrees, "mildly agitated" to "as angry as you have been in your life"
Index cards		E.g., with written scenarios, emotions, ambiguous situations
	INTERVENTION SUPPORT	
Support on demand		The subject of the intervention has the opportunity to request support if necessary
Feedback on performance		Subject receives feedback on performance and/or development
Peer feedback		The subject receives some sort of feedback from the other subjects in the group
Group performance		E.g., perform scenarios for the group
Reward based		E.g., group members receive some form of reinforcement for participation
External monitoring		Intervention deliverer, researchers, teachers, peers or others monitor (logging, registering, checking) the subjects performance on intervention elements, or usage/dosage or intervention elements
Regular support		The subject receives some sort of intervention relevant support multiple times during the intervention period from an intervention deliverer without having to request it (e.g., training sessions over time, booster sessions, follow-up calls, etc.).
Youth influence		Explicitly stating that personal opinions or preferences from the youth participating in the

		intervention influenced the delivery of the intervention
Family influence		Explicitly stating that that personal opinions or preferences from the family participating in the intervention influenced the delivery of the intervention
Culturally sensitive		Clear indications is porivded that the intervention is culturally sensitive. E.g., training deliverers in relevant cultural sensitivity, using trained translators, doing adaptions of the intervention to fit minority groups, etc.
Multicomponent		Explicitly stating that the intervention consists of different elements, themes, or core topics
Flexible/adaptive		Explicitly stating that flexibility or use of adaptions in delivering the intervention was allowed or encouraged
Individualized		Explicitly stating that the delivery of the intervention was tailored, adjusted or adapted to individual needs or preferences
Pedagogical principles		
Engagement		E.g., family/youth willing to engage
Social ecological		E.g., intervention based on social-ecological principles
Strengths-based		E.g., intervention is strength-based/focus on the positive

Refer to additional support and services		
Feedback from participants		E.g., participants give feedback on how they experience the intervention
Variable name in SPSS	Implementation elements	Strategies for ensuring the practice and process elements are set in motion according to the original program's expectations
	FIDELITY	
Therapist fidelity		e.g. specific measurements (TAM), audio recordings, questionnaires to measure therapist's fidelity to treatment/program
	ADHERENCE	
Boosters		Booster sessions after original training
Supervision		Supervision in the delivery of the intervention
Consultations		Consultations to discuss the implementation of the treatment protocol
Group training		Group training/coaching in the delivery of the intervention
	ACCEPTABILITY	
Participant satisfaction		Ratings of participant satisfaction (youth or parent/caregiver)
Participant involvement		Participant rates level of involvement, level of enjoyment and level of information learned

Participant acceptability		Participants rate their acceptability of the intervention
Participant appropriateness		Participants rate the quality of treatment, level of family involvement, change and effectiveness
